# Biomimetic synthesis of the natural product salviadione and its hybrids: discovery of tissue-specific anti-inflammatory agents for acute lung injury†
†Electronic supplementary information (ESI) available: Details of spectroscopic data and ^1^H and ^13^C NMR spectral copies of the products. CCDC 1520901. For ESI and crystallographic data in CIF or other electronic format see DOI: 10.1039/c9sc00086k


**DOI:** 10.1039/c9sc00086k

**Published:** 2019-03-21

**Authors:** Chunyong Ding, Hongjin Chen, Bin Liang, Mingkun Jiao, Guang Liang, Ao Zhang

**Affiliations:** a CAS Key Laboratory of Receptor Research , State Key Laboratory of Drug Research , Shanghai Institute of Materia Medica , Chinese Academy of Sciences , Shanghai 201203 , China . Email: aozhang@simm.ac.cn ; Fax: +86 (021) 50806035 ; Tel: +86 (021) 50806035; b Chemical Biology Research Center , School of Pharmaceutical Sciences , Wenzhou Medical University , Wenzhou , Zhejiang 325035 , China . Email: wzmcliangguang@163.com; c University of Chinese Academy of Sciences , Beijing 100049 , China

## Abstract

Acute lung injury (ALI) is an inflammatory disease with no effective pharmacological treatment. The therapeutic potential of the anti-inflammatory natural product tanshinone IIA (**2**) for ALI is seriously impaired by its poor pharmacokinetic (PK) properties. Inspired by the unique benzo[*def*]carbazole-3,5-dione (BCD) core of the natural product salviadione (**5**), a series of furan-fused BCD hybrids of **5** with **2** was rationally designed with the aim to improve both PK properties and the anti-inflammatory activity. A biomimetic synthetic approach featuring one-pot tandem N-heterocyclization was first developed for convenient assembly of salviadione (56% overall yield over 2 steps) and the designed hybrids (35–85% yields in one step). Compared to **2**, most of the resulting compounds exhibited a markedly enhanced inhibitory effect against LPS-induced release of pro-inflammatory cytokines in macrophages. Particularly, compound **15a** not only possessed the most potent activity *in vitro*, but also exhibited significantly improved metabolic stability (4- to 7-fold enhancement), pharmacokinetic properties (*T*_1/2_ = 4.05 h; *F* = 30.2%), and preferable lung tissue distribution (11- to 300-fold selectivity). An *in vivo* study in mice showed that pretreatment with **15a** at 5 mg kg^–1^ distinctly attenuated LPS-induced ALI *via* lung tissue-specific anti-inflammatory actions, indicating that the furan-fused BCD core presents a unique chemotype with promising therapeutic potential for ALI.

## Abstract 2

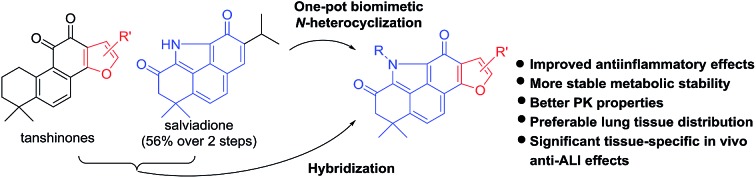
Biomimetic synthesis of the natural product salviadione and its hybrids was achieved leading to tissue-specific anti-inflammatory agents for acute lung injury.

## Introduction

Acute lung injury (ALI) is an unmet medical need and currently has no effective therapies.^1^ It is believed that the inflammatory storm is a critical contributor to the occurrence of ALI,^2^ and many released pro-inflammatory cytokines, such as tumor necrosis factor-α (TNF-α), interleukin-6 (IL-6) and IL-1β, are key players in the progression of ALI.^3^ As such, blocking the excessive production of these cytokines by anti-inflammatory agents including corticosteroids represents a promising strategy to prevent and treat this disease.^4^ However, most of these agents have no favorable benefits to ALI patients due to low efficacy and severe side effects.^5^ Therefore, novel anti-inflammatory agents with high efficacy and better safety are highly needed for ALI treatment.

**Fig. 1:**
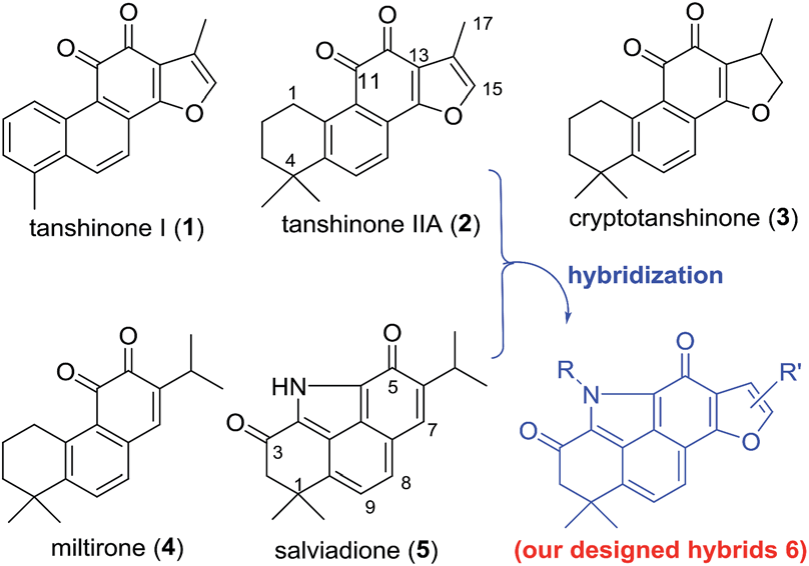
Tanshinones (**1–4**), salviadione (**5**), and our designed hybrids **6**.

Natural products are generally valuable starting points for drug discovery. Tanshinones, such as tanshinone I (**1**), tanshinone IIA (**2**), cryptotanshinone (**3**), and miltirone (**4**), represent a group of lipophilic quinoidal diterpenes from the traditional Chinese anti-inflammatory medicine *Salvia miltiorrhiza* (“Danshen”) (Fig. 1).^6^ The anti-inflammatory effect of **2** with therapeutic potential for ALI has been well documented.^7^ Nevertheless, further clinical investigation of **2** to treat ALI is seriously impaired by its modest potency and poor pharmacokinetic (PK) properties likely arising from the *ortho*-quinone moiety.^8^ Since *ortho*-quinone is a major structural determinant for the bioactivity of tanshinones, attempts to replace or mask this moiety often counteract its bioactivity.^9^ Therefore, development of novel chemical approaches to modify the *ortho*-quinone of **2** both to improve PK properties and to retain or improve the anti-inflammatory activity still remains an unsolved challenge.

**Fig. 2:**
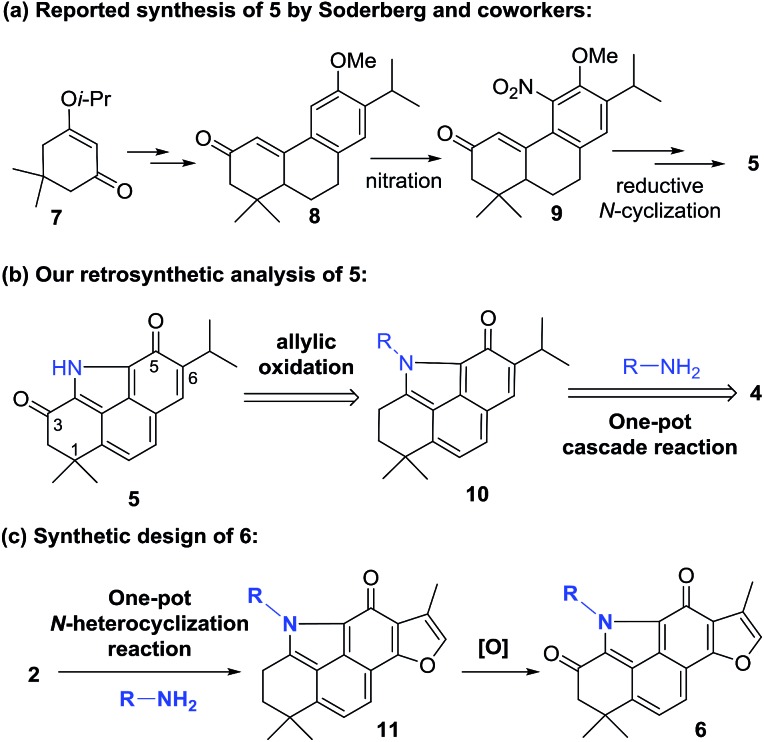
Reported synthesis of **5** and our synthetic strategy for **5** and **6**.

Salviadione (**5**) is another lipophilic ingredient also isolated from “Danshen” but lacking the *ortho*-quinone moiety (Fig. 1).^10^ Though **5** was barely studied due to its extremely low natural abundance, its unique structure bearing a benzo[*def*]carbazole-3,5-dione (BCD) core attracted our attention. Considering the same origin as that of **2**, we propose that hybridization of **5** with **2** would deliver a new compound series **6** bearing the furan-fused BCD core (Fig. 1) that may retain or improve the anti-inflammatory activity of **2**. Although the tetracyclic alkaloid **5** was first synthesized in 2011 by Söderberg and co-workers with a palladium-catalyzed CO-mediated reductive N-heterocyclization as the key step (Fig. 2a),^11^ long reaction steps and harsh reaction conditions make this approach unfeasible for the construction of **5** on a large scale. Since both compounds **5** and **4** were isolated from the same plant, it is likely that **5** is biogenetically derived from **4** under an ammonia atmosphere (Fig. 2b). As such, hybrid **6** might be accessed from **2** through a similar reaction pathway (Fig. 2c). In this report, we first describe our efforts to establish a biomimetic synthesis of **5** from **4**, and then apply this strategy to synthesize **6** from **2** (or its analogues). Subsequently, the anti-inflammatory activity of these compounds potentially for treating ALI is investigated as well.

## Results and discussion

### Biomimetic synthesis of natural product **5**

**Scheme 1:**
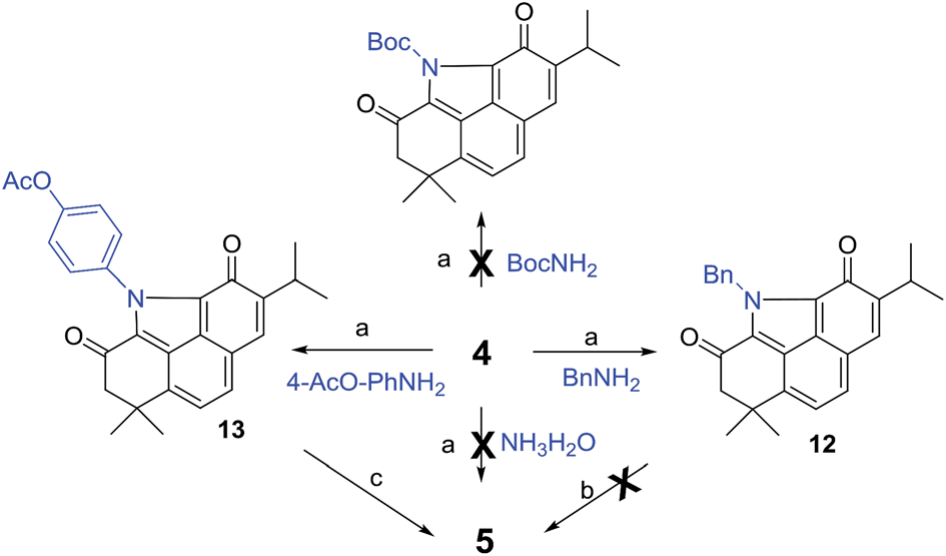
Biomimetic synthesis of **5**. Reagents and conditions: (a) TEMPO, toluene, 120 °C, 48 h, and 60–62%; (b) 10% Pd/C, H_2_, and EtOH; (c) (i) K_2_CO_3_, MeOH, and rt; (ii) PhI(OAc)_2_, CH_3_CN/H_2_O, 0 °C, and 90% (2 steps).

Inspired by our recent success in the C_sp^3^_–H acyloxylation or aryloxylation of **2**,^12^ we attempted to convert **4** to **5** through amination with various amines by using TEMPO as the oxidant (Scheme 1). Initially, with ammonia (7.0 M solution in MeOH) or BocNH_2_ as the amino source, the reaction failed to give the desired BCD products. Gratifyingly, when benzyl amine was used, the desired *N*-benzyl product **12** was obtained in 60% yield. After optimization of the reaction conditions (Table S1†), treating the mixture of **4**, benzyl amine, and TEMPO in toluene at 120 °C was identified as the optimal condition. Unfortunately, subsequent N-deprotection of the benzyl group catalyzed by Pd/C (10%) under a H_2_ atmosphere resulted in a complex mixture rather than the expected **5**. Alternatively, 4-aminophenyl acetate was selected as the amination reagent. Pleasingly, the corresponding benzo[*def*]carbazole **13** was achieved in 62% yield under the optimal conditions. Subsequent hydrolysis with K_2_CO_3_ followed by treatment with PhI(OAc)_2_ successfully delivered the natural product **5** in 90% yield. The spectroscopic data of the synthetic **5** were in complete agreement with those of the isolated alkaloid^10^,^11^ (Table S2†). Thus, by means of a one-pot N-heterocyclization reaction, the biomimetic synthesis of **5** was accomplished from **4** in 56% overall yield over 2 steps.

### Synthesis of hybrid derivatives of the natural product **2** with **5**

**Scheme 2:**
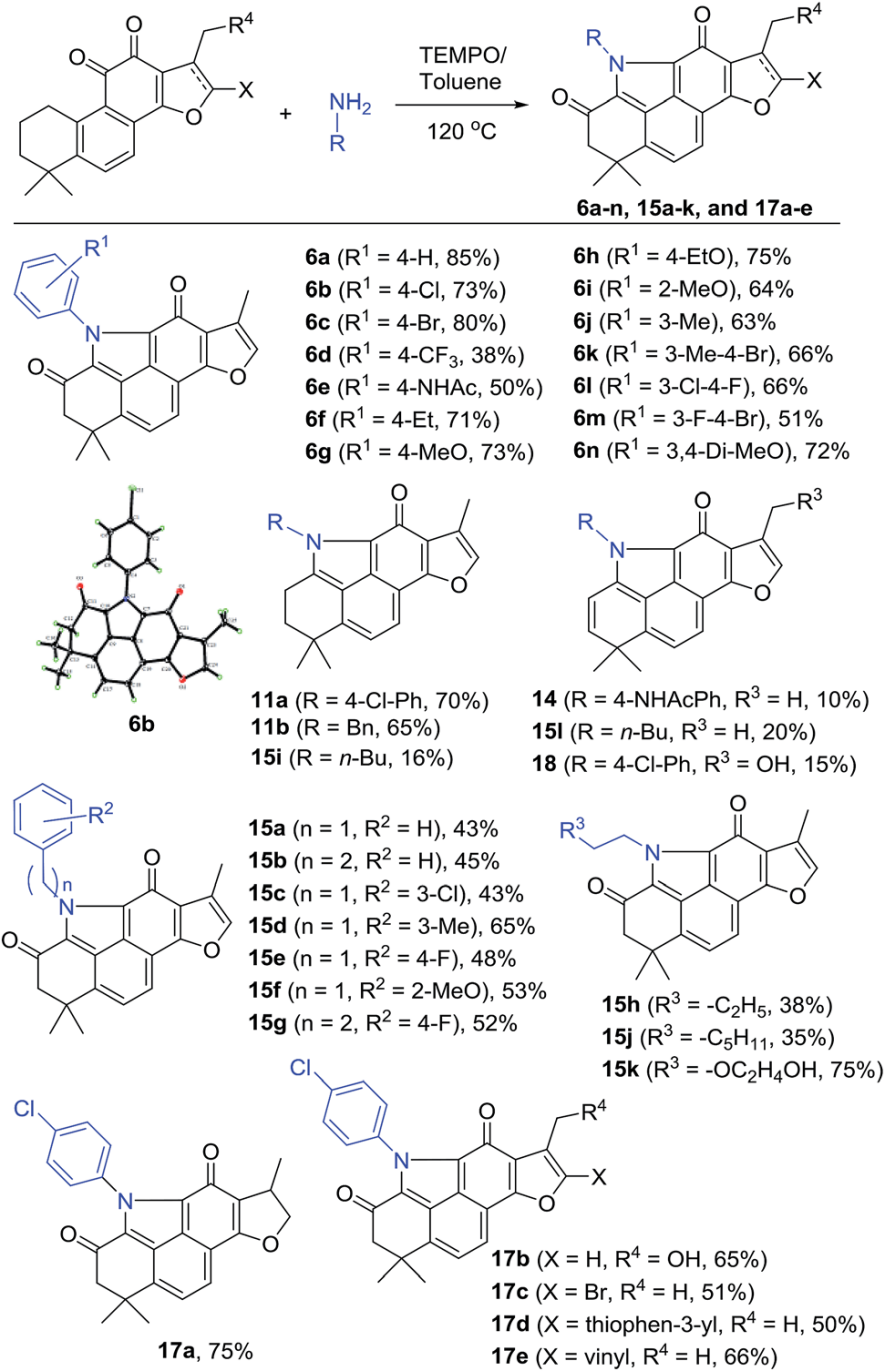
Synthesis of compounds **6a–n**, **15a–k**, and **17a–e**.

To incorporate the unique structural feature of alkaloid **5** into **2**, we designed compound series **6** bearing the benzo[*def*]carbazole core. As shown in Scheme 2, treating **2** with various anilines under the same reaction conditions as those for **12** provided the *N*-aryl products **6a–n** with furan-fused BCDs in 38–85% yields (Scheme 2). Among them, aniline exhibited the highest yield (85% for **6a**). *para*-Halogenated anilines were found to give high yields (73% for **6b** and 80% for **6c**). The structure of **6b** was unambiguously confirmed by single X-ray crystallographic analysis (Fig. S1†). Nevertheless, the aniline substrate with the *para*-trifluoromethyl group led to a dramatically decreased yield (38% for **6d**). Anilines with electron-donating groups at the *ortho*-, *meta*-, and *para*-positions were well tolerated to provide **6e–j** in 50–75% yields. In the case of *N*-(4-aminophenyl)acetamide, in addition to the major product **6e** (50%), a side product **14** with the 2-ene functionality was obtained in 10% yield. 3,4-Di-substituted anilines were also compatible with the reaction and the corresponding products **6k–n** were obtained in 51–72% yields.

In addition to anilines, various alkyl amines were found to take part in the reaction as well providing the *N*-alkyl products **15a–h** and **15j–k** in relatively lower yields (mostly 35–53% except **15d** and **15k**), likely due to the high nucleophilic properties of alkyl amines that might undergo other side reactions (Scheme 2). For example, in the case of butan-1-amine, byproducts **15i** and **15l** were isolated in 16% and 20% yields, respectively, together with the major product **15h** (38%). Other tanshinone analogues including **3** were also suitable as the substrate to generate the corresponding products **17a–e** in 50–75% yields under the standard reaction conditions. In the case of the 17-hydroxyl substrate, the expected product **17b** was obtained in 65% yield, together with the 2-ene byproduct **18** in 15% yield.

The isolated byproducts **11a–b**, **14**, **15i**, **15l**, and **18** indicated that the reaction may occur through a unique tandem N-heterocyclization process including condensation of 11-ketone of **2** with amines followed by successive imine–enamine tautomerism, intramolecular 1,6-aza-conjugate addition, and dehydrogenative aromatization to generate a key intermediate **11**, such as **11a–b** and **15i**. Further allylic oxidation of **11** with TEMPO provides the final products. Alternatively, allylic hydroxylation of **11** followed by elimination led to side products 2-enes, such as **14**, **15l** and **18** (Fig. S2 in the ESI†). Indeed, in our control experiments, treatment of **2** with 4-chloroaniline or benzylamine in an argon atmosphere without oxidants yielded **11a–b** in 70% and 65% yields, respectively, which were further transferred to corresponding **6b** and **15a** by TEMPO, respectively (Scheme S1†).

### *In vitro* bioactivity of selected hybrid derivatives

Since TNF-α and IL-6 are two well-known pro-inflammatory cytokines that mainly contribute to inflammation-induced ALI, we first tested the inhibitory effects of compounds **2**, **5**, **6a–n**, **15a–k** and **17a–e** at 10 μM against their release induced by LPS in mouse primary peritoneal macrophages. L6H21 (**19**)4*b* was used as the positive control. Most of the synthesized hybrids show markedly improved inhibitory effects (inhibition rates >50%) than the parent compounds **2** and **5** (Fig. S3†), while lacking significant cytotoxic effects on the cell viability at 10 μM (Fig. S4†), indicating that their anti-inflammatory activity was not ascribed to the cell death or injury. Compounds **6b–c**, **15a–b**, **15f**, and **15h** suppressed LPS-induced production of IL-6 and TNF-α in a dose-dependent manner (Fig. S5†), further validating their significant anti-inflammatory effects. Particularly, **15a** exhibited the most potent activity against the release of IL-6 and TNF-α with IC_50_ values of 1.62 μM and 6.37 μM, respectively. Compound **15a** was further found exhibiting negligible hERG inhibition with an IC_50_ value greater than 40 μM, indicating its low risk of cardiac toxicity (Table S3†).

### Metabolic and pharmacokinetic properties of **15a**

Compare to **2**, compound **15a** possessed about 4- to 7-fold enhanced metabolic stability in both human and rat microsomes (Table 1). Also, it exhibited markedly improved overall pharmacokinetic (PK) properties with a half-life (*T*_1/2_) of 4.05 h and an oral bioavailability (F) of 30.2% (Table S4†) when compared to those of **2** (*T*_1/2_ = 0.34 h; *F* < 3.5% (ref. 8a)). A tissue distribution study showed that **15a** was mainly distributed in the lung with time extending. At 12 h, the lung drug concentration is about 11 to 300 times higher than that in other tissues (Table 2), indicating the selective lung accumulation of **15a** suitably for treating ALI.

**Table 1:** *In vitro* metabolic stability in liver microsomes

Compound	Species	*T*_1/2_ (min)	Clint *in vitro* (mL min^–1^ g^–1^ protein)
**15a**	Human	34.09	61.62
	Rat	23.48	89.44
**2**	Human	7.58	277.17
	Rat	3.15	666.40

**Table 2:** Tissue distribution of **15a** following a single intravenous administration (2 mg kg^–1^)^*a*^

Tissue	Concentration of **15a**		
	0.25 h	2 h	12 h
Heart plasma (ng mL^–1^)	144	31.2	1.90
Portal vein plasma (ng mL^–1^)	142	33.1	2.04
Heart (ng g^–1^)	992	223	19.8
Liver (ng g^–1^)	2557	518	57.3
Lung (ng g^–1^)	3320	847	633
Kidney (ng g^–1^)	806	187	33.0
Brain (ng g^–1^)	224	93.9	3.11

^*a*^Six rats were assigned to each group.

### *In vivo* anti-ALI effect of **15a**

**Fig. 3:**
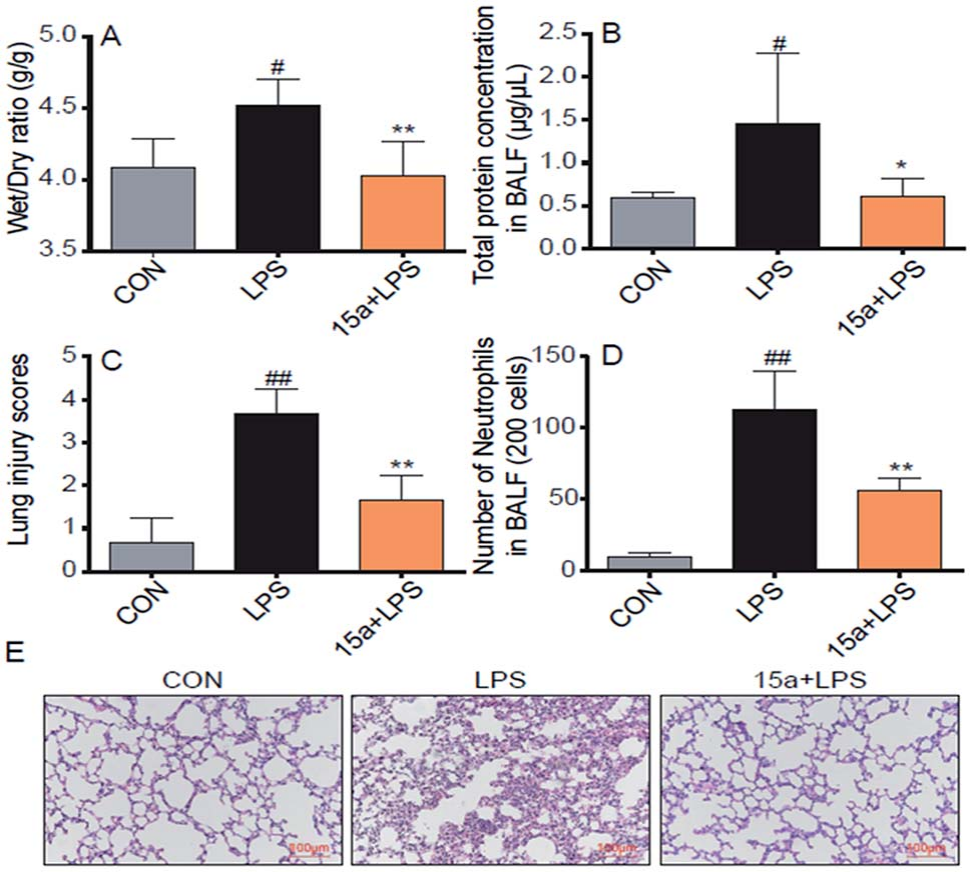
Compound **15a** attenuated the LPS-induced ALI in mice. (A) Wet/dry ratio. (B) The protein concentration in BALF. (C) Number of neutrophils in BALF. (D) The histogram of lung injury scores. (E) Compound **15a** attenuated the LPS-induced histopathological change in lung tissue (H&E staining). Data were presented as mean ± S.E.M. *p < 0.05, **p < 0.01 *vs.* LPS group, #*p* < 0.05, ##*p* < 0.01 *vs.* CON group, and *n* = 7 per group.

Encouraged by the potent *in vitro* anti-inflammatory activity as well as the promising drug-like properties, the *in vivo* anti-ALI effect of **15a** was further evaluated. As shown in Fig. 3A, the lung wet/dry weight ratio, an index of lung edema, was significantly increased after LPS-stimulation, compared with the control group. Nevertheless, pretreatment with **15a** at 5 mg kg^–1^ effectively reduced the lung wet/dry ratio, indicating that **15a** suppressed LPS-induced lung edema. The protein concentration in mice bronchial alveolar lavage fluid (BALF) is an important indicator for the structural integrity of the alveolar wall. It was found that the protein concentration of mice BALF was markedly increased after LPS instillation, whereas the increase was significantly inhibited by pretreatment with **15a** (Fig. 3B). Recruitment of neutrophils into the pulmonary compartment is also an important characteristic of ALI. The LPS challenge led to a strong increase in the number of neutrophils in the BALF (Fig. 3D), which was also significantly decreased by pretreatment with **15a**. In addition, the effects of **15a** on the histopathological features of ALI mice lungs were also tested (Fig. 3C and E). Compared to the normal structure of mice lung tissues, the LPS treatment remarkably increased the alveolar wall thickness, hemorrhage, alveolar collapse, and inflammatory infiltration in the lungs (Fig. 3E, middle panel). As a comparison, the group pretreated with 5 mg kg^–1^ of **15a** displayed very little histopathological changes, when compared to the normal lung structure. In Fig. 3C, the injury score of lung tissues is well correlated with the histopathological changes as shown in Fig. 3E. All the results indicate that **15a** effectively attenuates LPS-induced ALI in mice with optimal treatment potential for ALI.

**Fig. 4:**
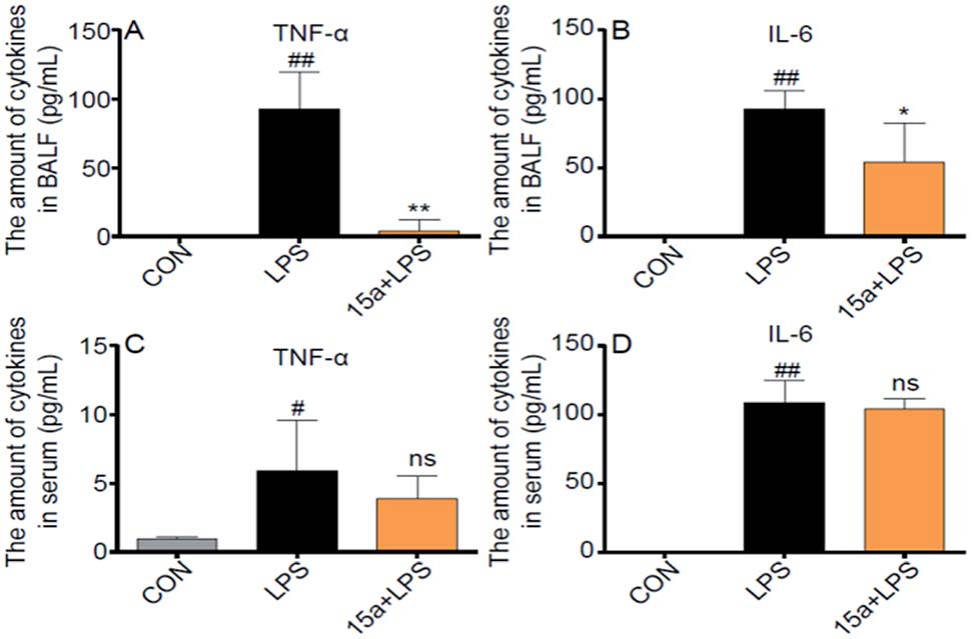
Effects of compound **15a** on LPS-induced acute lung inflammation in mice. (A) The amount of TNF-α in BALF. (B) The amount of IL-6 in BALF. (C) The amount of TNF-α in the serum. (D) The amount of IL-6 in the serum. Data were presented as mean ± S.E.M. *p < 0.05, **p < 0.01 *vs.* LPS group, ^#^*p* < 0.05, ^##^*p* < 0.01, ns = not significant, *vs.* CON group, and *n* = 7 per group.

Considering that pro-inflammatory cytokines play critical roles in the progression of ALI, the production levels of TNF-α and IL-6 in the BALF and serum from ALI mice were then examined as biomarkers of the *in vivo* anti-ALI effects of **15a.** As shown in Fig. 4A and B, the levels of TNF-α and IL-6 in the BALF of LPS-induced ALI mice were distinctly suppressed by pretreatment with **15a** at 5 mg kg^–1^; in contrast, no significant inhibition was observed in the serum (Fig. 4C and D) probably due to the low distribution of **15a** in the plasma, demonstrating that **15a** possesses a unique anti-inflammatory activity selectively in lung tissue, thus probably avoiding potential side effects arising from systematic inhibition of pro-inflammatory cytokines.

**Fig. 5:**
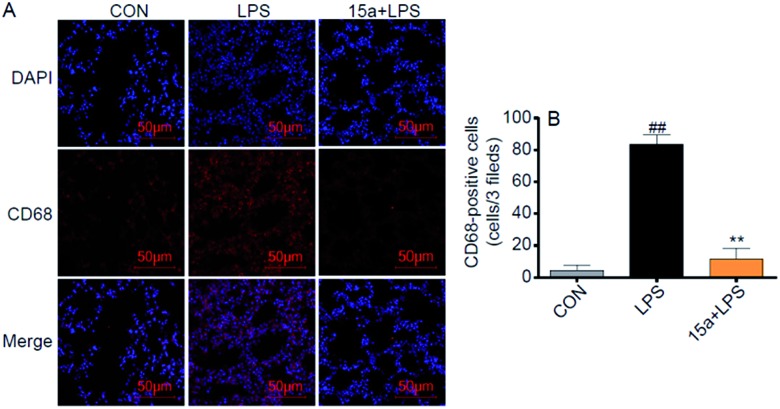
Compound **15a** exhibited the inhibitory effect against macrophage infiltration. (A) Representative images of CD68/DAPI immunohistochemical staining in the mice lung tissues (CD68 = red; nuclei = blue); scale bars = 50 μm. (B) Quantification of CD68 positive cells from 7 mice. **p < 0.01 *vs.* LPS group and ##*p* < 0.01 *vs.* CON group.

Besides, **15a** markedly suppressed the mRNA expression of pro-inflammatory cytokines, including TNF-α, IL-6, and IL-1β, and the adhesion molecule intercellular cell adhesion molecule-1 (ICAM-1), in the LPS-treated lung tissues (Fig. S6†). To validate the inhibitory effect of **15a** against macrophage infiltration into the lung, immunohistochemistry analysis of CD68, a macrophage marker, was performed in the lung tissue. Pretreatment with **15a** at 5 mg kg^–1^ significantly attenuated LPS-induced lung macrophage infiltration into the lung, as evidenced by CD68-immunostaining in Fig. 5. These data indicate that **15a** improves ALI therapy *via* its anti-inflammatory actions including decreasing inflammatory gene expression and inhibiting macrophage infiltration.

## Conclusions

In summary, we have developed a biomimetic synthetic approach featuring one-pot tandem N-heterocyclization that allows for convenient assembly of the natural product salviadione in 56% overall yield over 2 steps as well as a series of unique hybrids of **5** with **2** bearing a furan-fused BCD core. Most of the resulting compounds exhibited markedly improved *in vitro* anti-inflammatory effects. Particularly, **15a** was identified as the most potent with an IC_50_ value of 1.62 μM against the release of IL-6 induced by LPS in macrophages. In addition to its negligible cellular and cardiovascular toxicity, when compared to the natural product **2**, the derivative **15a** exhibited significantly improved drug-like properties including metabolic stability (4- to 7-fold enhancement), pharmacokinetic properties (*T*_1/2_ = 4.05 h; *F* = 30.2%) and preferable tissue distribution in the lung (11- to 300-fold selectivity). More importantly, **15a** effectively attenuates LPS-induced ALI in mice *via* lung tissue-specific anti-inflammatory actions, thus confirming the furan-fused BCD core as a unique chemotype with ALI therapeutic potential.

## Ethical statements

Protocols involving the use of animals were approved by the Wenzhou Medical University Animal Policy and Welfare Committee, and all experiments were performed in compliance with the Animal Ethics Procedures and Guidelines of the People's Republic of China.

## Conflicts of interest

There are no conflicts of interest to declare.

## Supplementary Material

Supplementary information

Crystal structure data
