# Supplementary material for: Biomimetic synthesis of the natural product salviadione and its hybrids: discovery of tissue-specific anti-inflammatory agents for acute lung injury
Source: Chem Sci. 2019 Mar 21;10(17):4667–72. doi: 10.1039/c9sc00086k (PMC6498537; doi:10.1039/c9sc00086k)

Electronic Supplementary Material (ESI) for Chemical Science.
This journal is © The Royal Society of Chemistry 2019

Biomimetic Synthesis of Natural Product Salviadione and Its Hybrids: Discovery of Tissue-Specific Anti-Inflammatory Agents for Acute Lung Injury

Chunyong Ding[†,§], Hongjin Chen[‡], Bin Liang[†], Mingkun Jiao[†], Guang Liang[*,‡], Ao Zhang[*,†,§]

[†]CAS Key Laboratory of Receptor Research, State key Laboratory of Drug Research, Shanghai Institute of *Materia Medica*, Chinese Academy of Sciences, Shanghai 201203, China

[‡]Chemical Biology Research Center, School of Pharmaceutical Sciences, Wenzhou Medical University, Wenzhou, Zhejiang 325035, China

[§]University of Chinese Academy of Sciences, Beijing 100049, China.

Content

Table S1--**S3**

Table S2--**S4-S5**

Figure S1--**S6**

Scheme S1 and Figure S2---**S7**

Figure S3-- **S8**

Figure S4-- **S9**

Figure S5-- **S10**

Figure S5-- **S10**

Table S3-S4--**S11**

Figure S6---**S12**

Biological experiment procedure---**S13-19**

Synthetic experiment procedure and spectroscopic data---------------------**S20-S35**

Copies of ^{1}H and ^{13}C NMR spectra of final products--------------------------**S36-S72**

Table S1. Optimization of Reaction Conditions for Synthesis of **12**[a]

Entry	Solvent	Oxidant	Yield (%)[b]
2	Toluene	O_2	53
3	Toluene	$AgCO_3$	55
4	Toluene	AgOAc	58
5	Toluene	Ag_2O	45
6	Toluene	MnO_2	36
7	Toluene	$Cu(OAc)_2$	48
9	Toluene	BQ	52
10	Toluene	**TEMPO**	**60**
11	Toluene	$PhI(OAC)_2$	43
13	Toluene	TBHP	0
14	PhCl	TEMPO	59
15	o-xylene	TEMPO	55
16	DCE	TEMPO	34
17	1,4-dioxane	TEMPO	25
18	*t*-BuOH	TEMPO	30

[a]Reaction condition: miltirone (0.1 mmol), phenylmethanamine (0.11 mmol), oxidant (0.13 mmol), solvent (1 mL), 120 ^{o}C, 48 h; [b]Isolated yield.

Table S2. Comparisons of the spectroscopic data of our synthesized salviadione with those reported by literature.

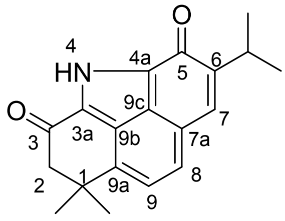

Reported salviadione (500 MHz, $CDCl_3$) [a]			Our synthesized salviadione (**5**) (300 MHz, $CDCl_3$)	
position	$\delta_{C,}$ type	δ_H(J in Hz)	δ_C	δ_H (J in Hz)
1(4)	41.3, C		42.03	
2 (3)	57.4, CH_2	2.95 s	58.12	2.97 s
3 (2)	188.0, C		188.67	
3a (1)	128.2, C		128.95	
4a (11)	126.9, C		127.59	
5 (12)	175.9, C		176.71	
6 (13)	151.4, C		152.12	
7 (14)	129.1, CH	7.56 s	129.74	7.58 s
7a (8)	127.9, C		128.45	
8 (7)	129.7, CH	7.66 d (7.5)	130.36	7.68 d (7.2)
9 (6)	121.0, CH	7.30 d (7.5)	121.75	7.32 d (6.9)
9a (5)	145.3, C		145.92	
9b (10)	123.9, C		124.61	
9c (9)	124.3, C		125.04	
i-Pr (CH)	27.4, CH	3.54 m	28.13	3.58 m

i-Pr (Me)	22.8, CH_3	1.31 d (7.0)	23.49	1.33 d (6.9)
i-Pr (Me)	22.8, CH_3	1.31 d (7.0)	23.49	1.33 d (6.9)
C1 (Me)	30.1, CH_3	1.49 s	30.77	1.51 s
C1 (Me）	30.1, CH_3	1.49 s	30.77	1.51 s

[a]*J. Nat. Prod.* **2005**, *68*, 1066-1070. *Tetrahedron* **2011**, *67*, 4753–4757.

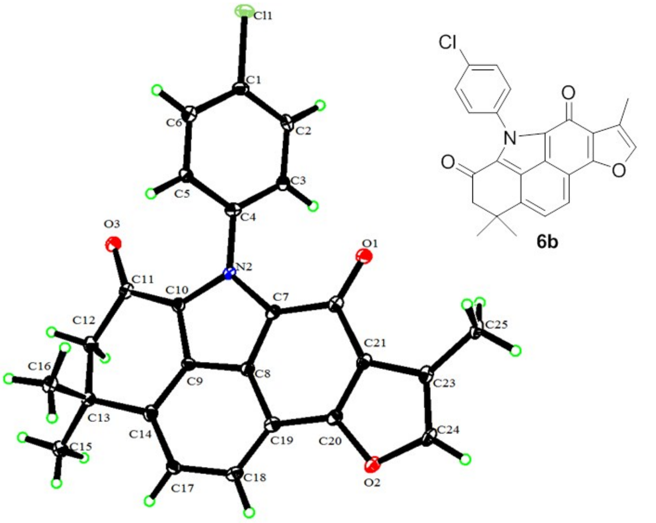

Figure S1. X-ray structure of **6b**. Deposition number in Cambridge Structural Database: CCDC1520901.

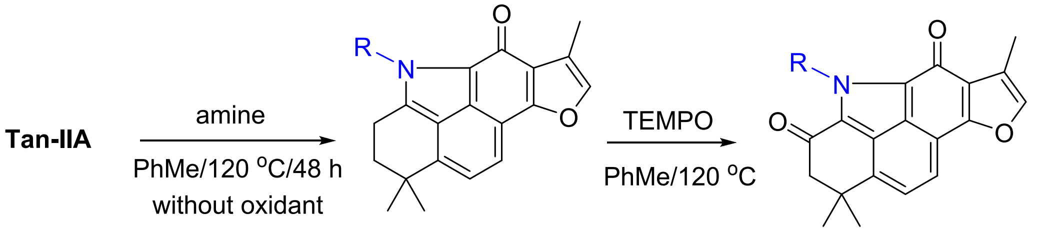

11a (R = 4-Cl-Ph, 70%)
11b (R = Bn, 65%)

6b (R = 4-Cl-Ph, 82%)
15a (R = Bn, 78%)

Scheme S1. Control reactions for the mechanism study.

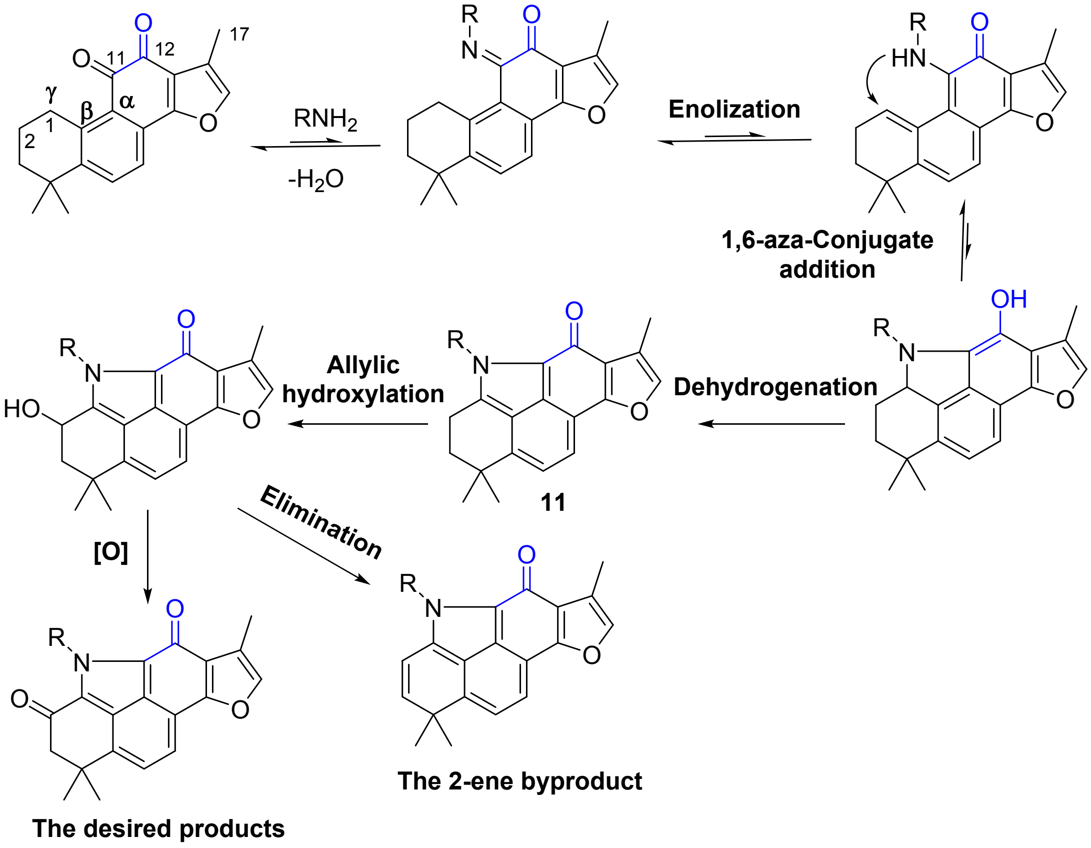

Figure S2. Proposed reaction mechanism

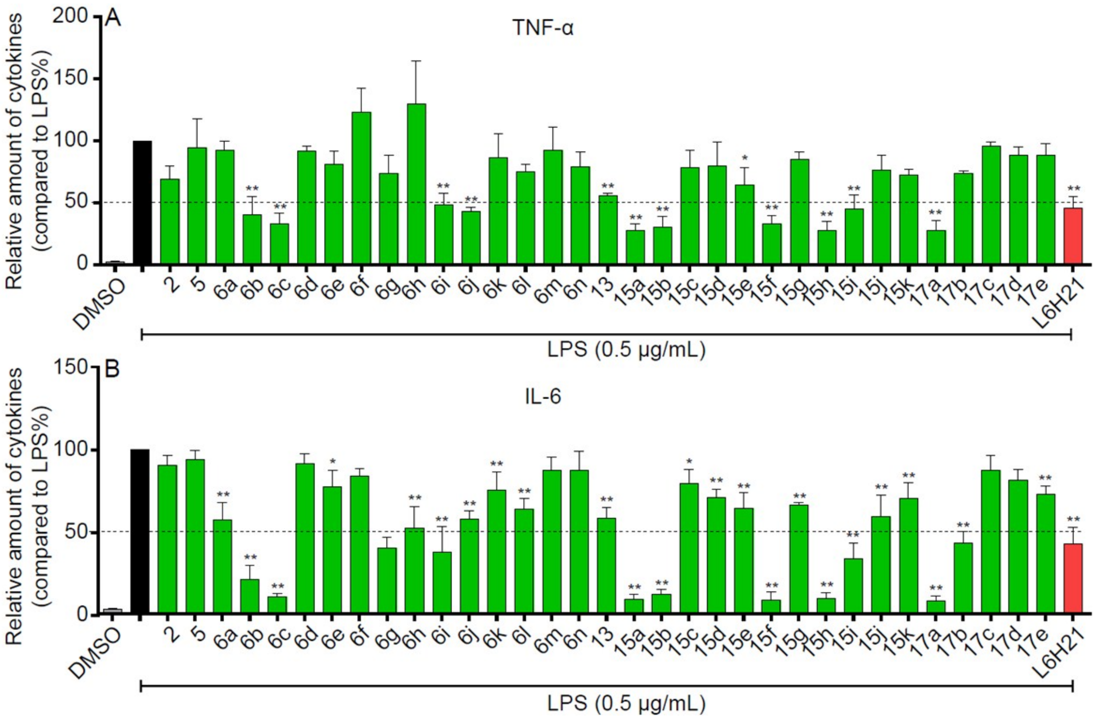

Figure S3. Inhibitory activity of the benzo[def]carbazole-3,5-diones against LPS-induced TNF-α and IL-6 secretion in mouse peritoneal macrophages. Macrophages were plated at a density of 5 × 10^5/plate for overnight at 37 °C and in 5% CO_2. Cells were pretreated with **5** or tanshinone analogues (10 μM) for 30 min, then treated with LPS (0.5 μg/mL) for 24 h. L6H21 (**19**) was used as a positive control. TNF-α and IL-6 levels in the culture medium were measured by ELISA and were normalized by the total protein level. The results were presented as the percent of LPS control. Each bar represents the mean ± SD of three independent experiments. Statistical significance relative to the LPS group is indicated: ∗, $p < 0.05$; ∗∗, $p < 0.01$.

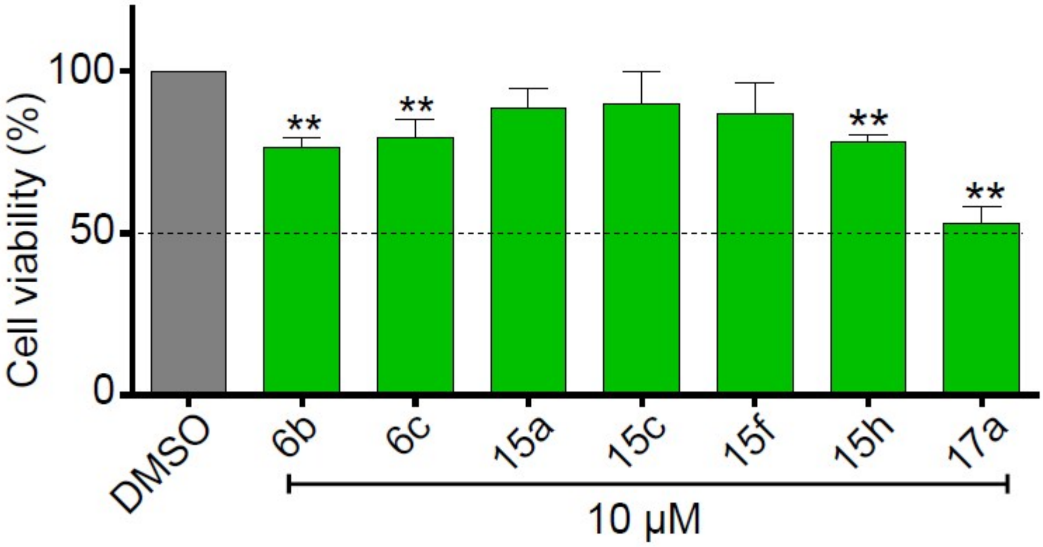

Figure S4. The cytotoxic evaluation in mouse peritoneal macrophages analyzed by an MTT assay. Macrophages (1×10^4/well) were seeded in 96-well plates and treated with salviadione analogues at the dose of 10 μM for 24 h. Statistical significance relative to the DMSO group is indicated: ∗∗, $p < 0.01$.

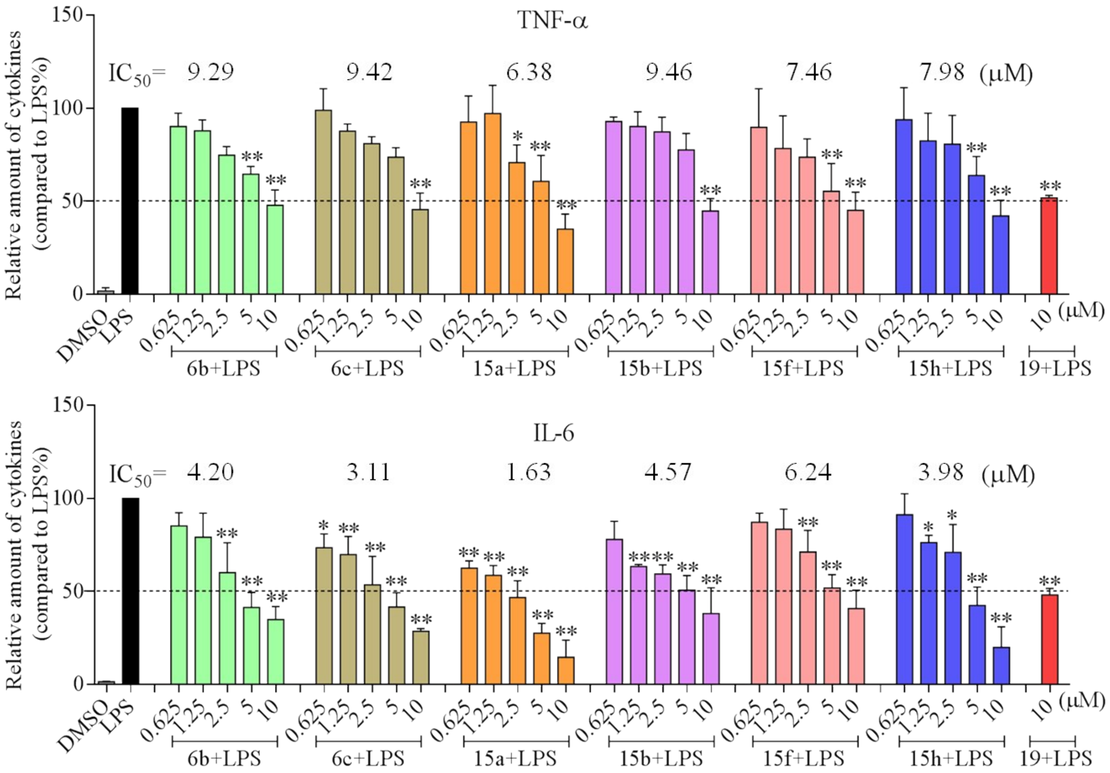

Figure S5. Inhibition of LPS-induced TNF-α and IL-6 release of the six potent compounds in a dose-dependent manner in MPMs. Macrophages were plated at a density of 5.0 × 10^5/plate at 37 °C and 5 % CO_2 overnight. Cells were pre-treated with tanshinone analogs in a series concentration of 0.625 μM, 1.25 μM, 2.5 μM, 5 μM, 10 μM and L6H21 (**19**) (10 μM) for 30 min, then treated with LPS (0.5 μg/mL) for 24 h. IL-6 and TNF-α levels in the culture media were measured by ELISA and were normalized by the total protein. The results were expressed as the percent of LPS. Each bar represents mean ± SEM of three independent experiments. Statistical significance relative to LPS group was indicated, *p<0.05, **p<0.01.

Table S3. Inhibition Assay of the hERG Potassium Ion Channel

Compounds	hERG inhibition IC_{50} (μM)
**15a**	>40
**Cisapride**	0.15

Table S4. Preliminary Pharmacokinetic Parameters for Compound 15a[a-c]

		$T_{1/2}$ (h)	T_{ma} (h)	C_{max} (ng/mL)	AUC (h*ng/mL)	$CL_{\_obs}$ (mL/min/kg)	MRT_{INF_obs} (h)	$Vss_{\_obs}$ (mL/kg)	F (%)
**15a**	*po*	4.05	1.83	81.2	426	--	5.33	--	30.2
	iv	5.00	--	--	485	34.9	2.98	5980	--

[a]Values are the average of three runs. Vehicle: DMSO, Tween 80, normal saline. CL, clearance; Vss, volume of distribution; $T_{1/2}$, half-life; C_{max}, maximum concentration; T_{max}, time of maximum concentration; MRT, mean residence time; AUC, area under the plasma concentration time curve; F, oral bioavailability. [b]Dose: p.o. at 3.0 mg/kg; [c]Dose: i.v. at 1.0 mg/kg

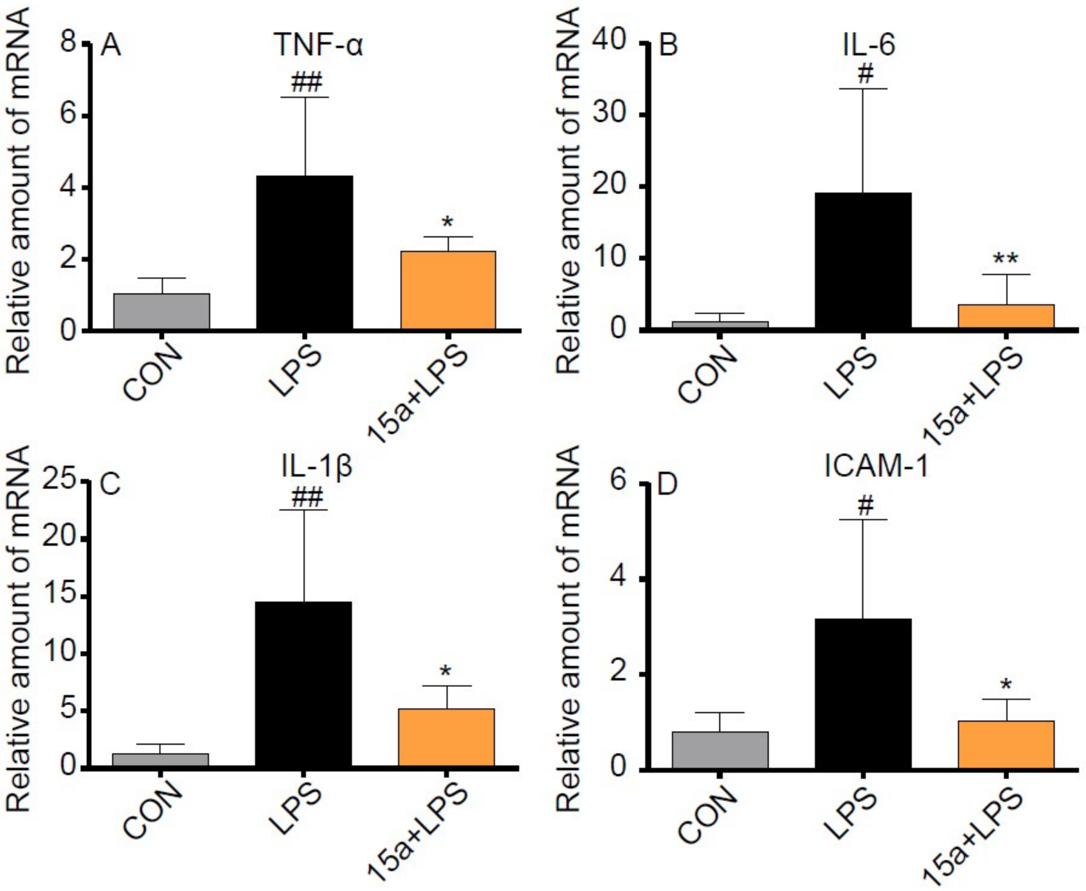

Figure S6. Effects of compound 15a on the LPS-induced mRNA expression of inflammatory genes. Mice was sacrificed and total RNA in lung was extracted. The mRNA levels of inflammatory cytokines TNF-α, IL-6, IL-1β, and ICAM-1 were detected by RT-qPCR (A–D). Data was presented as mean ± S.E.M. *p<0.05, **p<0.01 vs LPS group, #p<0.05, ##p<0.01 vs CON group, n = 7 per group.

hERG Cardiotoxicity Assay. The human embryonic kidney cells stably expressing hERG channels were used in the study. A Multi Clamp 700B manual patch clamp system was used to induce and record hERG currents of HEK293 cell in whole-cell voltage clamp mode. Compound 15a or positive control Cisapride at six various concentrations (40 μM, 13.33 μM, 4.44 μM, 1.48 μM, 0.49 μM, and 0.16 μM) and vehicle control were delivered separately to the clamped cells by a 6-channel perfusion system. The hERG channel tail currents were recorded by clampex 10.3. The data were collected and analyzed by clampfit 10.3. The peak of the tail currents in the presence of positive control and test article were normalized with the currents recorded in the presence of corresponding vehicle control as 100 %, respectively. The IC50 value was calculated using GraphPad Prism 5.00.

Liver Microsomal Stability Assay. Microsomes in 0.1 M TRIS buffer pH 7.4 (final concentration 0.33 mg/mL), co-factor $MgCl_2$ (final concentration 5 mM) and tested compound (final concentration 0.1 μM, co-solvent (0.01% DMSO) and 0.005% Bovin serum albumin) were incubated at 37°C for 10min. The reaction was started by the addition of NADPH (final concentration 1 mM). Aliquots were sampled at 0, 7, 17, 30 and 60 min, respectively, and methanol (cold in 4 °C) was added to terminate the reaction. After centrifugation (4000 rpm, 5 min), samples were then analyzed by LC-MS/MS.

Tissue Distribution Assay. Compound **15a** (2 mg/kg) dissolved in PEG300/EtOH/NaCl（40/10/50, v/v/v）to a concentration of 0.4 mg/mL, and was

given to SD rats (Male, 180 – 220 g, three groups with three rats in each group) by intravenous administration. Blood samples and the important tissues including heart, liver, lung, kidney, and brain were collected at 0.25, 2 and 12 h after administration (anticoagulant: EDTA-Na2). For plasma samples, 250 μL of solvent of methanol: acetonitrile (1:1, v/v) with internal standard was added to 50 μL of plasma and vortexed thoroughly. For tissue samples, three times the weight of volume of PBS were added, then homogenized. The homogenates were precipitated by five times of methanol: acetonitrile (1:1, v/v) with internal standard. They were centrifuged for 5 min, and then 20 μL of the supernatant was mixed with 20 μL of water for analysis. Samples were analyzed by AB6500 triple quadrupole mass spectrometer (AB Sciex, USA). The ACQUITY UPLC BEH C18 (1.7 μm, 2.0 mm × 50 mm, Waters, USA) was used for the analysis. Gradient elution was applied consisting of ultrapure water containing 0.1% formic acid and acetonitrile containing 0.1% formic acid.

Animals. Male ICR and C57BL/6 mice weighing 20-24 g were obtained from the Animal Center of Wenzhou Medical College (Wenzhou, China). Animals were housed at a constant room temperature with a 12:12 h light−dark cycle and fed with a standard rodent diet and water. The animals were acclimatized to the laboratory for at least 7 days before used in experiments. Protocols involving the use of animals were approved by the Wenzhou Medical College Animal Policy and Welfare Committee.

Reagents and Cells. Chemical reagents and lipopolysaccharide (LPS) were purchased from Sigma (St. Louis, MO). Saline was prepared as 0.9% NaCl solution. For

preparation of MPMs, ICR mice were stimulated by intraperitoneal (i.p) injection of 2 mL of thioglycollate solution (0.3 g of beef extract, 1 g of tryptone, 0.5 g of sodium chloride, and 6 g of soluble starch were dissolved and boiled in 100 mL of water; before use, the solution was filtrated with 0.22 μm filter) per mouse and kept in pathogen-free conditions for 3 days before peritoneal macrophage isolation. Total peritoneal macrophages were harvested by washing the peritoneal cavity with Roswell Park Memorial Institute (RPMI)-1640 medium (8 mL per mouse) and centrifuged. The pellet was then re-suspended in RPMI-1640 medium (Gibco, Eggenstein, Germany) with 10% FBS (Hyclone, Logan, UT), 100 U/mL penicillin, and 100 mg/mL streptomycin. Nonadherent cells were removed by washing with medium at 4 h after seeding. Experiments were undertaken after the cells adhered firmly to the culture plates. Before use, peritoneal macrophages were cultured in RPMI-1640 medium on 35 mm plates (5.0×10^5 cells / plate) and maintained at 37 °C in 5% CO_2-humidified air.

Determination of TNF-α and IL-6. The levels of TNF-α and IL-6 in medium, bronchoalveolar lavage fluid (BALF) and serum were determined with an ELISA kit (eBioScience, San Diego, CA) according to the manufacturer's instructions. The total amount of the inflammatory factor in the medium was normalized to the total protein quantity of the viable cell pellets.

Viability Assay. Mouse peritoneal macrophages (1×10^4 cells /plate) were seeded in 96-well plates and treated with salviadione analogues at the dose of 10 μM for 24 h. Cells were incubated in RPMI-1640 medium at 37 °C in 5% CO_2 for 24 hours. After

add MTT (5 mg/ml), the plates were incubated at 37 °C in 5% CO_2 for 4 hours. Cells were then dissolved with 150 μL dimethyl sulfoxide (DMSO), and the optical density was read at 490 nm. Cell viability was defined as the ratio (expressed as a percentage) of absorbance of treated cells to DMSO treated cells.

LPS-Induced Acute Lung Injury (ALI) in B6 Mice. Compound **15a** was first dissolved with macrogol 15 hydroxystearate (a nonionic solubilizer for injection from BASF) with or without medium chain triglycerides (MCT, from BASF) in a water bath at 37 °C. The concentration of compound was 2 mg/mL. The concentration of solubilizer was 5−10%, and that for MCT was 0.5−2% in final solution. For the vehicle, the mixture of solubilizer and MCT was prepared at 10% and 2%, respectively. Mice, weighing 20−24 g, were randomized to the following three groups of seven animals each: CON group, LPS group, **15a** + LPS group. **15a** + LPS group mice were pretreated with compounds in a water solution (5 mg/kg) by i.v injection 15 min before 5 mg/kg of LPS was administered by intratracheal instillation. The animals in CON group received a similar volume of vehicle. After 6 hours, mice were euthanized to collect the BALF, serum and lung tissue samples. Collection of the BALF was performed three times through a tracheal cannula with autoclaved physiological saline, instilled up to a total volume of 1 mL.

Lung Wet/Dry Ratio. The middle lobe of right lung was collected, and the wet weight was recorded. Lung was then heated in a thermostatic oven at 65 °C for 72 hours and weighed to determine the baseline lung dry mass levels.

Determination Total Protein Concentration and Number of Neutrophils in BALF. Bronchoalveolar lavage fluid (BALF) cells and supernatant were separated by centrifugal separation at 4 °C and 3,000 rpm for 10 minutes. Total protein concentration in supernatant of BALF was measured using a coomassie blue staining assay kit according to the manufacturer's instructions. The cells of BALF were resuspended in 40 μL of physiological saline and used for neutrophil cell counts by Wright–Giemsa stain.

Serum Analysis. Blood was collected from mice at sacrifice and was centrifuged at 4 °C and 12,000 rpm for 10 minutes. Serum supernatants were collected and stored at -80 °C before analysis.

Lung Histopathology and Immunohistochemistry Analysis. Lungs were removed and fixed in 4% formalin and embedded in paraffin, and sectioned at 5 μM. After dehydration, sections were stained with hematoxylin and eosin (H&E) for general histological examination. Each category was graded on a 0- to 4-point scale: 0 = no injury; 1 = injury up to 25% of the field; 2 = injury up to 50% of the field; 3 = injury up to 75% of the field; and 4 = diffuse injury. The immunohistochemistry analysis was performed following the staining protocol for the antiCD68 antibody (Santa Cruz, CA, USA).

Real-Time Quantitative PCR. Lung tissues were homogenized in TRIZOL kit (Invitrogen, Carlsbad, CA) for extraction of RNA according to each manufacturer's protocol. Both reverse transcription and quantitative PCR were carried out using a two-

step M-MLV Platinum SYBR Green qPCR SuperMix-UDG kit (Invitrogen, Carlsbad, CA). Eppendorf Mastercycler ep realplex detection system (Eppendorf, Hamburg, Germany) was used for RT-qPCR analysis. The primers of genes including TNF-α, IL-6, IL-1β, ICAM-1, and β-actin were synthesized by Invitrogen. The primer sequences of mouse genes used are shown as follows:

TNF-α sense primer, 5′-TGGAACTGGCAGAAGAGG-3

TNF-α antisense primer, 5′-AGACAGAAGAGCGTGGTG-3′;

IL-6 sense primer, 5′-GAGGATACCACTCCCAACAGACC-3′;

IL-6 antisense primer, 5′-AAGTGCATCATCGTTGTTCATACA-3′;

IL-1β sense primer, 5′-ACTCCTTAGTCCTCGGCCA-3′;

IL-1β antisense primer, 5′-CCATCAGAGGCAAGGAGGAA-3′;

ICAM-1 sense primer, 5′-GCCTTGGTAGAGGTGACTGAG-3′;

ICAM-1 antisense primer, 5′-GACCGGAGCTGAAAAGTTGTA-3′;

β-actin sense primer, 5′-TGGAATCCTGTGGCATCCATGAAAC-3′;

β-actin antisense primer, 5′-TAAAACGCAGCTCAGTAACAGTCCG-3′.

Statistical Analysis. The results are presented as the mean ±SD. The Student's t test was employed to analyze the differences between sets of data. Statistics were performed using GraphPad Pro (GraphPad, San Diego, CA). P values less than 0.05 ($p < 0.05$)

were considered indicative of significance. All experiments were repeated at least three times.

General Information for Chemical Synthesis. All reactions were performed in glassware containing a Teflon coated stir bar. Solvents and chemical reagents were obtained from commercial sources and used without further purifications. ^{1}H NMR spectral data were recorded in $CDCl_3$ on a Varian Mercury 300 or 400 NMR spectrometer, and ^{13}C NMR was recorded in $CDCl_3$ on a Varian Mercury 400 NMR spectrometer. Chemical shifts (δ) are reported in ppm downfield from an internal TMS standard. Low and high-resolution mass spectra were obtained in the ESI mode. Column chromatography on silica gel (200−300 mesh) was used for the routine purification of reaction products. The column output was monitored by TLC on silica gel (200−300 mesh) precoated on glass plates (15 mm × 50 mm), and spots were visualized by UV light at 254 or 365 nM. HPLC analysis was conducted for all bioassayed compounds on an Agilent Technologies 1260 series LC system (Agilent ChemStation Rev.A.10.02; ZORBAX-C18, 4.6 mm × 150 mm, 5 μM, MeOH (0.1% DEA)/H2O, rt) with two ultraviolet wavelengths (UV 254 and 214 nm). All the assayed compounds displayed a chemical purity of 95%−99% in both wavelengths. X-ray crystallographic image and data of compound **6b** are reported in Supplementary Figure 1S and Supplementary Tables, and its cif file was uploaded as Supplementary Data with CCDC number 1520901.

General Experimental Procedure for the Preparation of tested compounds **6a-n, 12-13, 14, 15a-k, 17a-e, and 18**: A 10 mL sealed tube was charged with tanshinones (0.1 mmol), various amines (0.11 mmol), and TEMPO (0.12 mmol). PhMe (1 mL) was

added, and the resulting mixture was stirred at 120 °C. The reaction was monitored by TLC until the starting material disappeared. The solvent was removed *in vacuo,* and the residue was purified using a silica gel column with CH_2Cl_2 as the eluent to give the benzo[*def*]carbazole-3,5-dione products as yellow solids.

1,1,6-Trimethyl-4-phenyl-1,4-dihydro-3*H*-benzo[*def*]furo[3,2-b]carbazole-3,5(2*H*)-dione (**6a**). Yellow solid (85%). ^{1}H NMR (300 MHz, $CDCl_3$) δ 7.86 (d, *J* = 6.9 Hz, 1H), 7.60 - 7.56 (m, 5H), 7.37 (d, *J* = 7.2 Hz, 1H), 7.32 (s, 1H), 2.92 (s, 2H), 2.35 (s, 3H), 1.53 (s, 6H). ^{13}C NMR (126 MHz, $CDCl_3$) δ 186.71, 173.62, 156.04, 143.96, 140.85, 136.55, 129.65, 128.77(2C), 128.31, 126.80 (2C), 126.46, 126.39, 125.37, 123.61, 122.71, 121.80, 120.66, 116.05, 57.83, 40.67, 29.91 (2C), 9.41. HRMS m/z (EI) calcd for $C_{25}H_{19}NO_3$ 381.1365, found 381.1366.

4-(4-Chlorophenyl)-1,1,6-trimethyl-1,4-dihydro-3*H*-benzo[*def*]furo[3,2-b]carbazole-3,5(2*H*)-dione (**6b**). Yellow solid (73%). ^{1}H NMR (300 MHz, $CDCl_3$) δ 7.86 (d, *J* = 7.2 Hz, 1H), 7.58 - 7.49 (m, 4H), 7.37 (d, *J* = 7.2 Hz, 1H), 7.32 (s, 1H), 2.92 (s, 2H), 2.34 (s, 3H), 1.52 (s, 6H). ^{13}C NMR (126 MHz, $CDCl_3$) δ 186.78, 173.59, 156.04, 143.97, 140.97, 135.60, 134.86, 128.97 (2C), 128.40, 128.22 (2C), 126.32, 126.23, 125.31, 123.67, 123.01, 121.76, 120.90, 116.03, 57.76, 40.66, 29.89 (2C), 9.38. HRMS m/z (EI) calcd for $C_{25}H_{18}ClNO_3$ 415.0975 found 415.0978.

4-(4-Bromophenyl)-1,1,6-trimethyl-1,4-dihydro-3*H*-benzo[*def*]furo[3,2-*b*]carbazole-3,5(2*H*)-dione (**6c**). Yellow solid (80%). ^{1}H NMR (300 MHz, $CDCl_3$) δ 7.86 (d, *J* = 6.9 Hz, 1H), 7.67 (d, *J* = 8.1 Hz, 2H), 7.50 (d, *J* = 8.1 Hz, 2H), 7.37 (d, *J* = 7.2 Hz, 1H),

7.33 (s, 1H), 2.92 (s, 2H), 2.35 (s, 3H), 1.53 (s, 6H). ^{13}C NMR (151 MHz, $CDCl_3$) δ 186.74, 173.57, 156.02, 143.96, 140.96, 135.37, 131.93 (2C), 128.50 (2C), 128.41, 126.25, 126.16, 125.31, 123.80, 123.69, 122.99, 121.76, 120.89, 116.04, 57.76, 40.66, 29.89 (2C) 9.37. HRMS m/z (EI) calcd for $C_{25}H_{18}BrNO_3$ 459.0470, found 459.0468.

1,1,6-Trimethyl-4-(4-(trifluoromethyl)phenyl)-1,4-dihydro-3*H*-benzo[def]furo[3,2-*b*]carbazole-3,5(2*H*)-dione (**6d**). Yellow solid (38%). ^{1}H NMR (300 MHz, $CDCl_3$) δ 7.88 (d, *J* = 6.9 Hz, 1H), 7.82 (d, *J* =7.8 Hz, 2H), 7.76 (d, *J* = 7.8 Hz, 2H), 7.39 (d, *J* = 6.9 Hz, 1H), 7.34 (s, 1H), 2.93 (s, 2H), 2.35 (s, 3H), 1.54 (s, 6H). ^{13}C NMR (126 MHz, $CDCl_3$) δ 186.78, 173.60, 156.05, 144.04, 141.06, 139.28, 131.60, 131.38, 128.56, 127.60 (2C), 126.33, 126.21, 125.93, 125.91, 125.30, 123.83, 123.24, 121.76, 121.10, 116.12, 57.75, 40.70, 29.89 (2C), 9.39. HRMS m/z (EI) calcd for $C_{26}H_{18}F_3NO_3$449.1239, found 449.1243.

N-(4-(1,1,6-trimethyl-3,5-dioxo-1,2,3,5-tetrahydro-4*H*-benzo[*def*]furo[3,2-*b*]carbazol-4-yl)phenyl)acetamide (**6e**). Yellow solid (50%). ^{1}H NMR (300 MHz, $CDCl_3$) δ 8.35 (s, 1H), 7.88 (d, *J* = 7.2 Hz, 1H), 7.66 (d, *J* = 8.4 Hz, 2H), 7.48 (d, *J* = 8.4 Hz, 2H), 7.38 (d, *J* = 7.2 Hz, 1H), 7.32 (s, 1H), 2.92 (s, 2H), 2.34 (s, 3H), 2.10 (s, 3H), 1.53 (s, 6H). ^{13}C NMR (126 MHz, $CDCl_3$) δ 187.52, 174.34, 169.18, 156.86, 144.83, 141.62, 140.51, 132.30, 128.94, 127.71 (2C), 127.40, 127.05, 126.01, 124.30, 123.68, 122.37, 121.41, 120.20 (2C), 116.57, 58.55, 41.39, 30.61 (2C), 25.27, 10.08. HRMS m/z (EI) calcd for $C_{27}H_{22}N_2O_4$ 438.1580, found 438.1574.

4-(4-Ethylphenyl)-1,1,6-trimethyl-1,4-dihydro-3*H*-benzo[*def*]furo[3,2-*b*]carbazole-3,5(2*H*)-dione (**6f**). Yellow solid (71%). ^{1}H NMR (300 MHz, $CDCl_3$) δ 7.85 (d, *J* = 7.2 Hz, 1H), 7.52 (d, *J* = 7.2 Hz, 2H), 7.40 - 7.34 (m, 3H), 7.32 (s, 1H), 2.91 (s, 2H), 2.78 (q, *J* = 7.5 Hz, 2H), 2.35 (s, 3H), 1.52 (s, 6H), 1.33 (t, *J* = 7.5 Hz, 3H). ^{13}C NMR (126 MHz, $CDCl_3$) δ 188.10, 175.02, 157.44, 147.03, 145.33, 142.21, 135.50, 129.69, 129.56 (2C), 127.94 (2C), 127.80, 126.78, 125.01, 123.98, 123.22, 121.94, 117.41, 59.29, 42.04, 31.31 (2C), 30.09, 16.42, 10.84. HRMS m/z (EI) calcd for $C_{27}H_{23}NO_3$ 409.1678, found 409.1681.

4-(4-Methoxyphenyl)-1,1,6-trimethyl-1,4-dihydro-3*H*-benzo[*def*]furo[3,2-*b*]carbazole-3,5(2*H*)-dione (**6g**). Yellow solid (73%). ^{1}H NMR (300 MHz, $CDCl_3$) δ 7.84 (d, *J* = 7.2 Hz, 1H), 7.53 (d, *J* = 8.4 Hz, 2H), 7.35 (d, *J* = 7.2 Hz, 1H), 7.32 (s, 1H), 7.05 (d, *J* = 8.4 Hz, 2H), 3.89 (s, 3H), 2.91 (s, 2H), 2.35 (s, 3H), 1.52 (s, 6H). ^{13}C NMR (126 MHz, $CDCl_3$) δ 188.15, 175.04, 161.66, 157.45, 145.29, 142.21, 130.66, 129.63, 129.32 (2C), 127.87, 127.80, 126.79, 124.94, 123.97, 123.21, 121.94, 117.36, 115.31 (2C), 59.28, 56.90, 42.03, 31.31 (2C), 10.83. HRMS m/z (EI) calcd for $C_{26}H_{21}NO_4$ 411.1471, found 411.1476.

4-(4-Ethoxyphenyl)-1,1,6-trimethyl-1,4-dihydro-3*H*-benzo[*def*]furo[3,2-*b*]carbazole-3,5(2*H*)-dione (**6h**). Yellow solid (75%). ^{1}H NMR (300 MHz, $CDCl_3$) δ 7.84 (d, *J* =6.9 Hz, 1H), 7.51 (d, *J* = 8.1 Hz, 2H), 7.35 (d, *J* = 7.2 Hz, 1H), 7.31 (s, 1H), 7.03 (d, *J* = 8.4 Hz, 2H), 4.11 (q, *J* = 6.9 Hz, 2H), 2.91 (s, 2H), 2.35 (s, 3H), 1.52 (s, 6H), 1.46 (t, *J* = 6.9 Hz, 3H). ^{13}C NMR (126 MHz, $CDCl_3$) δ 186.74, 173.63, 159.71, 156.04, 143.87,

140.78, 129.04, 128.20, 127.85(2C), 126.47, 126.40, 125.37, 123.51, 122.53, 121.80, 120.50, 115.94, 114.32(2C), 63.70, 57.87, 40.60, 29.89(2C), 14.88, 9.40. HRMS m/z (EI) calcd for $C_{27}H_{23}NO_4$ 425.1627 found 425.1627.

4-(2-Methoxyphenyl)-1,1,6-trimethyl-1,4-dihydro-3*H*-benzo[*def*]furo[3,2-*b*]carbazole-3,5(2*H*)-dione (**6i**). Yellow solid (64%). ^{1}H NMR (300 MHz, $CDCl_3$) δ 7.84 (d, *J* = 6.9 Hz, 1H), 7.51 - 7.49 (m, 2H), 7.43 - 7.28 (m, 2H), 7.14 - 7.10 (m, 2H), 3.75 (s, 3H), 3.01 - 2.80 (m, 2H), 2.35 (s, 3H), 1.52 (s, 3H), 1.50 (s, 3H). ^{13}C NMR (126 MHz, $CDCl_3$) δ 186.58, 173.55, 156.03, 154.24, 143.92, 140.69, 130.92, 128.07, 128.01, 126.85, 126.32, 126.19, 125.31, 123.41, 122.45, 121.75, 120.49, 120.32, 116.09, 112.11, 57.62, 55.81, 40.69, 30.23, 29.62, 9.40. HRMS m/z (EI) calcd for $C_{26}H_{21}NO_4$ 411.1471, found 411.1467.

1,1,6-Trimethyl-4-(*m*-tolyl)-1,4-dihydro-3*H*-benzo[*def*]furo[3,2-*b*]carbazole-3,5(2*H*)-dione (**6j**). Yellow solid (63%). ^{1}H NMR (300 MHz, $CDCl_3$) δ 7.85 (d, *J* = 7.2 Hz, 1H), 7.49 - 7.30 (m, 6H), 2.91 (s, 2H), 2.46 (s, 3H), 2.35 (s, 3H), 1.52 (s, 6H). ^{13}C NMR (151 MHz, $CDCl_3$) δ 186.65, 173.55, 156.01, 143.90, 140.80, 138.70, 136.54, 130.51, 128.56, 128.23, 127.28, 126.52, 126.49, 125.35, 123.75, 123.52, 122.57, 121.79, 120.56, 116.02, 57.83, 40.64, 29.91(2C), 21.51, 9.39. HRMS m/z (EI) calcd for $C_{26}H_{21}NO_3$ 395.1521, found 395.1529.

4-(4-Bromo-3-methylphenyl)-1,1,6-trimethyl-1,4-dihydro-3*H*-benzo[*def*]furo[3,2-*b*]carbazole-3,5(2*H*)-dione (**6k**). Yellow solid (66%). ^{1}H NMR (300 MHz, $CDCl_3$) δ 7.85 (d, *J* = 7.2 Hz, 1H), 7.70 (d, *J* = 8.4 Hz, 1H), 7.46 (s, 1H), 7.38 – 7.28 (m, 3H),

2.91 (s, 2H), 2.49 (s, 3H), 2.35 (s, 3H), 1.52 (s, 6H). ^{13}C NMR (126 MHz, $CDCl_3$) δ 188.12, 174.96, 157.44, 145.34, 142.33, 140.07, 136.99, 134.03, 130.36, 129.74, 127.76, 127.73, 127.04, 126.73, 125.02, 124.28, 123.19, 122.22, 117.45, 59.20, 42.08, 31.32(2C), 24.62, 10.79. HRMS m/z (EI) calcd for $C_{26}H_{20}BrNO_3$ 473.0627, found 473.0626.

4-(3-Chloro-4-fluorophenyl)-1,1,6-trimethyl-1,4-dihydro-3*H*-benzo[*def*]furo[3,2-*b*]carbazole-3,5(2*H*)-dione (**6l**). Yellow solid (66%). ^{1}H NMR (300 MHz, $CDCl_3$) δ 7.87 (d, *J* = 7.2 Hz, 1H), 7.68 (d, *J* = 4.5 Hz, 1H), 7.51 (s, 1H), 7.43 – 7.27 (m, 3H), 2.92 (s, 2H), 2.35 (s, 3H), 1.53 (s, 6H). ^{13}C NMR (126 MHz, $CDCl_3$) δ 188.15, 174.97, 161.04, 159.03, 157.44, 145.37, 142.45, 134.25, 130.88, 129.75, 128.37, 127.23, 125.02, 124.58, 123.16, 122.78, 122.62, 122.48, 118.09, 117.70, 59.11, 42.10, 31.31(2C), 10.75. HRMS m/z (EI) calcd for $C_{25}H_{17}ClFNO_3$ 433.0881, found 433.0879.

4-(4-Bromo-3-fluorophenyl)-1,1,6-trimethyl-1,4-dihydro-3*H*-benzo[*def*]furo[3,2-*b*]carbazole-3,5(2*H*)-dione (**6m**). Yellow solid (51%). ^{1}H NMR (300 MHz, $CDCl_3$) δ 7.87 (d, *J* = 6.9 Hz, 1H), 7.72 (t, *J* = 8.0 Hz, 1H), 7.43 -7.29 (m, 4H), 2.93 (s, 2H), 2.35 (s, 3H), 1.53 (s, 6H). ^{13}C NMR (151 MHz, $CDCl_3$) δ 186.69, 173.50, 159.34, 157.69, 156.00, 143.98, 141.06, 136.70, 133.33, 128.43, 126.21, 125.28, 123.98, 123.70, 123.24, 121.75, 121.12, 116.06, 115.85, 110.8, 57.69, 40.69, 29.88 (2C), 9.34. HRMS m/z (EI) calcd for $C_{25}H_{17}BrFNO_3$ 477.0376, found 477.0377.

4-(3,4-Dimethoxyphenyl)-1,1,6-trimethyl-1,4-dihydro-3*H*-benzo[*def*]furo[3,2-*b*]carbazole-3,5(2*H*)-dione (**6n**). Yellow solid (72%). ^{1}H NMR (300 MHz, $CDCl_3$) δ

7.85 (d, J = 6.9 Hz, 1H), 7.39 - 7.30 (m, 2H), 7.16 - 7.17 (m, 1H), 7.09 (s, 1H), 7.00 (d, J = 7.8 Hz, 1H), 3.96 (s, 3H), 3.91 (s, 3H), 2.92 (s, 2H), 2.36 (s, 3H), 1.53 (s, 6H). ^{13}C NMR (126 MHz, $CDCl_3$) δ 188.57, 175.40, 157.88, 151.75, 150.55, 145.75, 142.69, 131.23, 130.09, 128.44, 128.39, 127.25, 125.38, 124.46, 123.69, 122.46, 120.99, 117.84, 112.36, 112.25, 59.78, 58.04, 57.88, 42.50, 31.80(2C), 11.30. HRMS m/z (EI) calcd for $C_{27}H_{23}NO_5$ 441.1576, found 441.1577.

4-Benzyl-6-isopropyl-1,1-dimethyl-1,4-dihydro-3H-benzo[def]carbazole-3,5(2*H*)-dione (**12**). Yellow solid (60%). ^{1}H NMR (300 MHz, $CDCl_3$) δ 7.68 (d, J = 6.9 Hz, 2H), 7.61 (d, J = 6.9 Hz, 1H), 7.52 (s, 1H), 7.29 - 7.31 (m, 4H), 6.22 (s, 2H), 3.49 (s, 1H), 2.96 (s, 2H), 1.50 (s, 6H), 1.28 (s, 6H). ^{13}C NMR (126 MHz, $CDCl_3$) δ 188.60, 176.30, 161.85, 151.47, 144.04, 137.55, 128.67(2C), 128.63(2C), 128.44(2C), 128.16, 127.89, 126.12, 125.99, 123.78, 123.38, 57.97, 51.11, 40.85, 29.96 (2C), 27.01, 22.78 (2C). HRMS m/z (EI) calcd for $C_{28}H_{25}NO_5$ 383.1885, found 383.1889.

4-(6-Isopropyl-1,1-dimethyl-3,5-dioxo-1,2,3,5-tetrahydro-4*H*-benzo[*def*]carbazol-4-yl)phenyl acetate (**13**). Yellow solid (62%). ^{1}H NMR (300 MHz, $CDCl_3$) δ 7.64 - 7.61 (m, 3H), 7.51 (s, 1H), 7.32 (d, J = 7.2 Hz, 2H), 7.27 (s, 1H), 3.35 (q, J = 6.9 Hz, 1H), 2.92 (s, 2H), 2.34 (s, 3H), 1.51 (s, 6H), 1.21 (s, 3H), 1.19 (s, 3H). ^{13}C NMR (126 MHz, $CDCl_3$) δ 186.44, 174.66, 168.23, 151.42, 150.70, 143.79, 133.10, 128.30, 127.84, 127.77, 127.50 (2C), 126.02, 125.98, 123.80, 123.22, 121.03 (2C), 120.59, 57.72, 40.28, 29.35 (2C), 26.52, 22.31 (2C), 20.77. HRMS m/z (EI) calcd for $C_{27}H_{25}NO_4$ 427.1784, found 427.1783.

N-(4-(1,1,6-Trimethyl-5-oxo-1,5-dihydro-4*H*-benzo[*def*]furo[3,2-*b*]carbazol-4-yl)phenyl)acetamide (**14**). Yellow solid (10%). ^{1}H NMR (300 MHz, $CDCl_3$) δ 9.59 (s, 1H), 8.05 (d, *J* = 7.2 Hz, 1H), 7.73 (d, *J* = 8.4 Hz, 2H), 7.51 (d, *J* = 8.7 Hz, 2H), 7.37 (d, *J* = 4.2 Hz, 2H), 6.63 (d, *J* = 9.9 Hz, 1H), 6.47 (d, *J* = 9.9 Hz, 1H), 2.47 (s, 3H), 1.92 (s, 3H), 1.51 (s, 6H). ^{13}C NMR (126 MHz, $CDCl_3$) δ 169.29, 169.23, 154.14, 151.44, 145.33, 140.23, 140.20, 138.35, 131.08, 126.31 (2C), 126.01, 124.05, 123.90, 121.53, 121.13, 120.63, 120.50 (2C), 118.18, 113.29, 113.26, 40.37, 29.09 (2C), 24.23, 9.70. HRMS m/z (EI) calcd for $C_{27}H_{22}N_2O_3$ 422.1630 found 422.1628.

4-Benzyl-1,1,6-trimethyl-1,4-dihydro-3H-benzo[*def*]furo[3,2-b]carbazole-3,5(2*H*)-dione (**15a**). Yellow solid (43%). ^{1}H NMR (300 MHz, $CDCl_3$) δ 7.80 (d, *J* = 7.2 Hz, 1H), 7.62 (d, *J* = 7.8 Hz, 2H), 7.35 – 7.26 (m, 5H), 6.23 (s, 2H), 2.92 (s, 2H), 2.47 (s, 3H), 1.48 (s, 6H). ^{13}C NMR (126 MHz, $CDCl_3$) δ 188.88, 175.40, 157.00, 144.35, 141.45, 138.09, 129.36 (2C), 129.17 (2C), 128.85, 128.72, 126.47, 125.94, 123.89, 123.11, 122.34, 121.03, 116.44, 58.29, 51.81, 41.35, 30.66 (2C), 10.17. HRMS *m/z* (EI) calcd for $C_{27}H_{23}NO_3$ 395.1521, found 395.1526.

1,1,6-Trimethyl-4-phenethyl-1,4-dihydro-3*H*-benzo[*def*]furo[3,2-*b*]carbazole-3,5(2*H*)-dione (**15b**). Yellow solid (45%). ^{1}H NMR (300 MHz, $CDCl_3$) δ 7.82 (d, *J* = 7.2 Hz, 1H), 7.35 (s, 1H), 7.30 (d, *J* = 7.2 Hz, 1H), 7.24 - 7.16 (m, 5H), 5.24 (t, *J* = 7.5 Hz, 2H), 3.28 (t, *J* = 7.5 Hz, 2H), 2.77 (s, 2H), 2.48 (s, 3H), 1.44 (s, 6H). ^{13}C NMR (151 MHz, $CDCl_3$) δ 187.93, 174.50, 156.36, 143.65, 140.74, 137.37, 129.22(2C), 128.37(2C), 127.42, 126.66, 126.12, 125.53, 125.18, 123.10, 122.36, 121.61, 120.10,

115.56, 57.48, 49.66, 40.62, 37.66, 29.89(2C), 9.49. HRMS m/z (EI) calcd for $C_{27}H_{23}NO_3$ 409.1678, found 409.1679.

4-(3-Chlorobenzyl)-1,1,6-trimethyl-1,4-dihydro-3*H*-benzo[*def*]furo[3,2-*b*]carbazole-3,5(2*H*)-dione (**15c**). Yellow solid (43%). ^{1}H NMR (300 MHz, $CDCl_3$) δ 7.82 (d, *J* = 7.2 Hz, 1H), 7.56 (s, 1H), 7.50 (m, 1H), 7.32 (d, *J* = 7.5 Hz, 2H), 7.23 (d, *J* = 4.5 Hz, 2H), 6.20 (s, 2H), 2.92 (s, 2H), 2.45 (s, 3H), 1.49 (s, 6H). ^{13}C NMR (126 MHz, $CDCl_3$) δ 188.88, 175.39, 157.04, 144.40, 141.54, 139.75, 135.14, 130.65, 129.12, 129.08, 128.73, 127.36, 126.36, 125.93, 123.88, 123.36, 122.34, 121.24, 116.48, 58.20, 51.18, 41.40, 30.67(2C), 10.14. HRMS m/z (EI) calcd for $C_{26}H_{20}ClNO_3$ 429.1132, found 429.1134.

1,1,6-Trimethyl-4-(3-methylbenzyl)-1,4-dihydro-3*H*-benzo[*def*]furo[3,2-*b*]carbazole-3,5(2*H*)-dione (**15d**). Yellow solid (65%). ^{1}H NMR (300 MHz, $CDCl_3$) δ 7.80 (d, *J* = 7.2 Hz, 1H), 7.42-7.28 (m, 4H), 7.17 (t, *J* = 7.5 Hz, 1H), 7.06 (d, *J* = 7.5 Hz, 1H), 6.20 (s, 2H), 2.92 (s, 2H), 2.46 (s, 3H), 2.27 (s, 3H), 1.46 (s, 6H). ^{13}C NMR (126 MHz, $CDCl_3$) δ 188.82, 175.37, 156.99, 144.35, 141.42, 138.98, 137.96, 129.64, 129.60, 129.23, 128.71, 126.50, 126.02, 125.95, 123.89, 123.07, 122.36, 120.99, 116.44, 58.28, 51.81, 41.36, 30.67 (2C), 22.19, 10.17. HRMS m/z (EI) calcd for $C_{27}H_{23}NO_3$ 409.1678, found 409.1677.

4-(4-Fluorobenzyl)-1,1,6-trimethyl-1,4-dihydro-3*H*-benzo[*def*]furo[3,2-*b*]carbazole-3,5(2*H*)-dione (**15e**). Yellow solid (48%). ^{1}H NMR (300 MHz, $CDCl_3$) δ 7.80 (d, *J* = 7.2 Hz, 1H), 7.73 -7.60 (m, 2H), 7.37- 7.28 (m, 2H), 6.97 (t, *J* = 8.7 Hz, 2H), 6.19 (s,

2H), 2.92 (s, 2H), 2.46 (s, 3H), 1.48 (s, 6H). ^{13}C NMR (126 MHz, $CDCl_3$) δ 188.97, 175.43, 164.36, 162.40, 157.04, 144.38, 141.51, 134.02, 134.00, 131.31, 128.75, 126.31, 125.92, 123.89, 123.26, 122.31, 121.14, 116.44, 116.29, 116.12, 58.27, 50.98, 41.36, 30.65(2C), 10.16. HRMS m/z (EI) calcd for $C_{26}H_{20}FNO_3$ 413.1427, found 413.1419.

4-(2-Methoxybenzyl)-1,1,6-trimethyl-1,4-dihydro-3*H*-benzo[*def*]furo[3,2-*b*]carbazole-3,5(2*H*)-dione (**15f**). Yellow solid (53%). ^{1}H NMR (300 MHz, $CDCl_3$) δ 7.83 (d, *J* = 7.2 Hz, 1H), 7.39 -7.28 (m, 2H), 7.19 (t, *J* = 7.7 Hz, 1H), 6.87 (d, *J* = 8.1 Hz, 1H), 6.74 (t, *J* = 7.5 Hz, 1H), 6.52 (d, *J* = 7.5 Hz, 1H), 6.27 (s, 2H), 3.88 (s, 3H), 2.89 (s, 2H), 2.39 (s, 3H), 1.50 (s, 6H). ^{13}C NMR (126 MHz, $CDCl_3$) δ 188.29, 174.99, 157.61, 156.90, 144.33, 141.38, 129.20, 128.47, 127.27, 127.09, 126.72, 126.16, 125.93, 123.76, 122.96, 122.36, 121.12, 120.90, 116.48, 110.87, 58.24, 56.14, 48.32, 41.46, 30.66(2C), 10.08. HRMS m/z (EI) calcd for $C_{27}H_{23}NO_4$ 425.1627, found 425.1627.

4-(4-Fluorophenethyl)-1,1,6-trimethyl-1,4-dihydro-3*H*-benzo[*def*]furo[3,2-*b*]carbazole-3,5(2*H*)-dione (**15g**). Yellow solid (52%). ^{1}H NMR (300 MHz, $CDCl_3$) δ 7.83 (d, *J* = 7.2 Hz, 1H), 7.36 (s, 1H), 7.31 (d, *J* = 7.2 Hz, 1H), 7.22 -7.14 (m, 2H), 6.90 (t, *J* = 8.7 Hz, 2H), 5.21 (t, *J* = 7.5 Hz, 2H), 3.26 (t, *J* = 7.4 Hz, 2H), 2.79 (s, 2H), 2.47 (s, 3H), 1.45 (s, 6H). ^{13}C NMR (126 MHz, $CDCl_3$) δ 188.67, 175.20, 163.50, 161.55, 157.08, 144.34, 141.48, 133.73, 131.40, 128.14, 126.69, 126.24, 125.86, 123.77,

123.14, 122.28, 120.90, 116.27, 115.91, 115.74, 58.18, 50.27, 41.31, 37.45, 30.56(2C), 10.14. HRMS m/z (EI) calcd for $C_{27}H_{22}FNO_3$ 427.1584, found 427.1587.

4-Butyl-1,1,6-trimethyl-1,4-dihydro-3*H*-benzo[*def*]furo[3,2-*b*]carbazole-3,5(2*H*)-dione (**15h**). Yellow solid (38%). ^{1}H NMR (300 MHz, $CDCl_3$) δ 7.81 (d, *J* = 7.2 Hz, 1H), 7.32 (d, *J* = 7.5 Hz, 2H), 5.02 (t, *J* = 7.5Hz, 2H), 2.92 (s, 2H), 2.45 (s, 3H), 2.08-1.90 (m, 2H), 1.50 (s, 6H), 1.47- 1.38 (m, 2H), 0.96 (t, *J* = 7.5 Hz, 3H). ^{13}C NMR (126 MHz, $CDCl_3$) δ 187.55, 174.03, 155.82, 143.05, 140.18, 127.18, 125.48, 125.40, 124.67, 122.50, 121.67, 121.09, 119.57, 115.04, 57.14, 48.04, 40.14, 33.12, 29.45(2C), 19.32, 13.25, 8.93. HRMS m/z (EI) calcd for $C_{23}H_{23}NO_3$ 361.1678, found 361.1678.

4-Butyl-1,1,6-trimethyl-1,2,3,4-tetrahydro-5*H*-benzo[*def*]furo[3,2-*b*]carbazol-5-one (**15i**). Yellow solid (16%). ^{1}H NMR (400 MHz, $CDCl_3$) δ 7.89 (d, *J* = 7.2 Hz, 1H), 7.33 (d, *J* = 0.8 Hz, 1H), 7.16 (d, *J* = 7.6 Hz, 1H), 4.63 (t, *J* = 7.2 Hz, 2H), 3.12 (t, *J* = 6.0 Hz, 2H), 2.52 (d, *J* = 0.8 Hz, 3H), 2.16 (t, *J* = 6.4 Hz, 2H), 2.09-1.92 (m, 2H), 1.46 - 1.141 (m, 8H), 0.97 (t, *J* = 0.8 Hz, 3H). ^{13}C NMR (126 MHz, $CDCl_3$) δ 169.88, 154.01, 145.55, 139.42, 125.05, 123.52, 122.47, 121.13, 119.79, 119.68, 115.29, 113.37, 46.82, 40.26, 34.46, 32.49, 29.24, 27.58 (2C), 20.11, 19.65, 13.34, 9.22. HRMS m/z (EI) calcd for $C_{23}H_{25}NO_2$ 347.1885 found 347.1891.

4-Hexyl-1,1,6-trimethyl-1,4-dihydro-3*H*-benzo[*def*]furo[3,2-*b*]carbazole-3,5(2*H*)-dione (**15j**). Yellow solid (35%). ^{1}H NMR (300 MHz, $CDCl_3$) δ 7.82 (d, *J* = 7.2 Hz, 1H), 7.32 (d, *J* = 7.2 Hz, 2H), 5.01 (t, *J* = 7.5 Hz, 2H), 2.92 (s, 2H), 2.45 (s, 3H), 2.07 - 1.92 (m, 2H), 1.50 (s, 6H), 1.32 - 1.25 (m, 6H), 0.87 (d, *J* = 6.3 Hz, 3H). ^{13}C NMR

(126 MHz, $CDCl_3$) δ 187.58, 174.08, 155.88, 143.11, 140.23, 127.24, 125.52, 125.44, 124.73, 122.57, 121.71, 121.16, 119.61, 115.12, 57.19, 48.27, 40.19, 31.13, 30.94, 29.49(2C), 25.78, 22.11, 13.56, 8.97. HRMS m/z (EI) calcd for $C_{25}H_{27}NO_3$ 389.1991, found 389.1992.

4-(2-(2-Hydroxyethoxy)ethyl)-1,1,6-trimethyl-1,4-dihydro-3*H*-benzo[*def*]furo[3,2-*b*]carbazole-3,5(2*H*)-dione (**15k**). Yellow solid (75%).^{1}H NMR (300 MHz, $CDCl_3$) δ 7.83 (d, *J* = 6.9 Hz, 1H), 7.33 (d, *J* = 6.6 Hz, 2H), 5.25 (t, *J* = 5.1 Hz, 2H), 4.06 (t, *J* = 5.1 Hz, 2H), 3.59 (s, 4H), 2.93 (s, 2H), 2.43 (s, 3H), 1.50 (s, 6H). ^{13}C NMR (126 MHz, $CDCl_3$) δ 189.26, 175.50, 157.09, 144.47, 141.49, 128.49, 127.34, 126.78, 125.87, 123.84, 123.33, 122.27, 121.03, 116.28, 73.00, 71.25, 62.43, 58.36, 48.61, 41.41, 30.63(2C), 10.09. HRMS m/z (EI) calcd for $C_{23}H_{23}NO_5$ 393.1576, found 393.1576.

4-(4-Chlorophenyl)-1,1,6-trimethyl-1,4,6,7-tetrahydro-3*H*-benzo[*def*]furo[3,2-*b*]carbazole-3,5(2*H*)-dione (**17a**). Yellow solid (75%). ^{1}H NMR (300 MHz, $CDCl_3$) δ 7.78 (d, *J* = 7.2 Hz, 1H), 7.57 (d, *J* = 8.7 Hz, 2H), 7.49 (d, *J* = 8.4 Hz, 2H), 7.35 (d, *J* = 7.2 Hz, 1H), 4.88 (t, *J* = 9.3 Hz, 1H), 4.48-4.33 (m, 1H), 3.61 (brs, 1H), 2.91 (s, 2H), 1.52 (s, 6H), 1.35 (d, *J* = 6.6 Hz, 3H). ^{13}C NMR (126 MHz, $CDCl_3$) δ 188.17, 175.61, 165.29, 148.49, 137.37, 136.58, 130.66(2C), 130.23, 130.13(2C), 128.46, 128.20, 127.35, 126.34, 125.91, 122.45, 118.01, 83.30, 59.59, 42.68, 37.30, 31.98, 31.50, 21.17. HRMS m/z (EI) calcd for $C_{25}H_{20}ClNO_3$ 417.1132, found 417.1129.

4-(4-Chlorophenyl)-6-(hydroxymethyl)-1,1-dimethyl-1,4-dihydro-3*H*-benzo[*def*]furo[3,2-*b*]carbazole-3,5(2*H*)-dione (**17b**). Yellow solid (65%). ^{1}H NMR

(300 MHz, $CDCl_3$) δ 7.98 (d, *J* = 7.5 Hz, 1H), 7.60 - 7.51 (m, 4H), 7.50 (s, 1H), 7.43 (d, *J* = 7.2 Hz, 1H), 4.69 (d, *J* = 6.9 Hz, 2H), 4.57 (t, *J* = 6.9 Hz, 1H), 2.95 (s, 2H), 1.54 (s, 6H). ^{13}C NMR (126 MHz, $CDCl_3$) δ 188.83, 175.18, 158.77, 147.26, 142.09, 137.79, 136.45, 130.95 (2C), 130.11 (2C), 129.45, 128.07, 127.69, 127.52, 126.42, 125.89, 123.01, 117.34, 59.82, 57.29, 42.79, 31.85(2C). HRMS m/z (EI) calcd for $C_{25}H_{18}ClNO_4$ 431.0924, found 431.0933.

7-Bromo-4-(4-chlorophenyl)-1,1,6-trimethyl-1,4-dihydro-3*H*-benzo[*def*]furo[3,2-*b*]carbazole-3,5(2*H*)-dione (**17c**). Yellow solid (51%). ^{1}H NMR (300 MHz, $CDCl_3$) δ 7.89 (d, *J* = 7.2 Hz, 1H), 7.63 - 7.45 (m, 4H), 7.39 (d, *J* = 7.2 Hz, 1H), 2.93 (s, 2H), 2.31 (s, 3H), 1.53 (s, 6H). ^{13}C NMR (126 MHz, $CDCl_3$) δ 186.26, 171.65, 155.19, 144.03, 135.23, 134.21, 128.51(2C), 127.78, 127.68(2C), 126.18, 125.58, 125.19, 123.38, 122.88, 122.62, 120.67, 120.49, 114.60, 57.31, 40.27, 29.39(2C), 9.47. HRMS m/z (EI) calcd for $C_{25}H_{17}BrClNO_3$ 493.0080, found 493.0074.

4-(4-Chlorophenyl)-1,1,6-trimethyl-7-(thiophen-3-yl)-1,4-dihydro-3*H*-benzo[*def*]furo[3,2-*b*]carbazole-3,5(2*H*)-dione (**17d**). Yellow solid (50%). ^{1}H NMR (300 MHz, $CDCl_3$) δ 7.91 (d, *J* = 7.2 Hz, 1H), 7.63 - 7.49 (m, 6H), 7.46 (d, *J* = 2.7 Hz, 1H), 7.38 (d, *J* = 7.2 Hz, 1H), 2.93 (s, 2H), 2.60 (s, 3H), 1.54 (s, 6H). ^{13}C NMR (126 MHz, $CDCl_3$) δ 186.25, 173.21, 153.28, 147.60, 143.39, 135.11, 134.40, 131.23, 128.47(2C), 127.87, 127.75(2C), 126.06, 125.90, 125.77, 125.68, 125.08, 123.04, 122.45, 120.82, 120.46, 116.27, 115.22, 57.31, 40.19, 29.41(2C), 9.52. HRMS m/z (EI) calcd for $C_{29}H_{20}ClNO_3S$ 497.0852, found 497.0847.

4-(4-Chlorophenyl)-1,1,6-trimethyl-7-vinyl-1,4-dihydro-3*H*-benzo[*def*]furo[3,2-*b*]carbazole-3,5(2*H*)-dione (**17e**). Yellow solid (66%). ^{1}H NMR (300 MHz, $CDCl_3$) δ 7.91 (d, *J* = 7.2 Hz, 1H), 7.58 – 7.50 (m, 4H), 7.38 (d, *J* = 7.2 Hz, 1H), 6.70 - 6.57 (m, 1H), 5.87 (d, *J* = 17.4 Hz, 1H), 5.35 (d, *J* = 11.4 Hz, 1H), 2.92 (s, 2H), 2.38 (s, 3H), 1.53 (s, 6H). ^{13}C NMR (126 MHz, $CDCl_3$) δ 188.67, 175.35, 156.41, 152.21, 146.03, 137.51, 136.73, 130.87 (2C), 130.27, 130.11 (2C), 128.36, 128.27, 128.07, 125.59, 125.17, 124.43, 122.85, 121.10, 117.51, 114.90, 59.67, 42.60, 31.80 (2C), 11.08. HRMS m/z (EI) calcd for $C_{27}H_{20}ClNO_3$ 441.1132, found 441.1124.

4-(4-Chlorophenyl)-6-(hydroxymethyl)-1,1-dimethyl-1,4-dihydro-5*H*-benzo[*def*]furo[3,2-*b*]carbazol-5-one (**18**) Yellow solid (15%). ^{1}H NMR (300 MHz, $CDCl_3$) δ 8.13 (d, *J* = 7.5 Hz, 1H), 7.66 (d, *J* = 8.4 Hz, 2H), 7.59 (d, *J* = 8.7 Hz, 2H), 7.51 (s, 1H), 7.41 (d, *J* = 7.5 Hz, 1H), 6.70 (d, *J* = 9.9 Hz, 1H), 6.57 (d, *J* = 9.9 Hz, 1H), 5.80 (brs, 1H), 4.74 (s, 2H), 1.52 (s, 6H). ^{13}C NMR (126 MHz, $CDCl_3$) δ 170.20, 156.42, 154.36, 147.81, 140.96, 140.64, 137.30, 136.62, 131.45(2C), 129.60(2C), 128.74, 128.23, 126.75, 126.27, 122.77, 122.40, 120.57, 115.00, 57.55, 42.46, 30.84(2C). HRMS m/z (EI) calcd for $C_{25}H_{18}ClNO_3$ 415.0975, found 415.0950.

6-Isopropyl-1,1-dimethyl-1,4-dihydro-3*H*-benzo[*def*]carbazole-3,5(2*H*)-dione (salvidione, **5**). To a solution of **13** (14 mg, 0.05 mmol) in methanol (1 mL) was added K_2CO_3 (14 mg, 0.1 mmol) at rt. The resulting reaction mixture was stirred at rt. The reaction was monitored by TLC until the starting material disappeared. The solvent was removed in vacuo, and the resulting residue was diluted with CH_2Cl_2 (5 mL), washed

with 5% HCl aqueous solution, and dried with anhydrous Na_2SO_4. The solvent was removed in vacuo to give the brown residue. Without further purification, the residue was reacted with $PhI(OCOCF_3)_2$ (26 mg, 0.06 mmol) in a mixture of MeCN and H_2O (1 mL, 1:1) at 0 °C for 6 h. The reaction mixture was r was diluted with CH_2Cl_2 (5 mL), washed with saturated NaCl aqueous solution, and dried with anhydrous Na2SO4. The solvent was removed in vacuo to give the oil residue, which was purified using a silica gel column with CH_2Cl_2/MeOH = 60:1 as the eluent to give the natural product **5** (13 mg, 90%) as yellow solid. ^1H NMR (300 MHz, $CDCl_3$) δ 7.68 (d, J = 7.2 Hz, 1H), 7.58 (s, 1H), 7.32 (d, J = 7.2 Hz, 1H), 3.58 (m, 1H), 2.97 (s, 2H), 1.51 (s, 6H), 1.33 (d, J = 6.9 Hz, 6H). ^{13}C NMR (126 MHz, $CDCl_3$) δ 188.67, 176.71, 152.12, 145.92, 130.36, 129.74, 128.95, 128.45, 127.59, 125.04, 124.61, 121.75, 58.12, 42.03, 30.77 (2C), 28.13, 23.49 (2C). HRMS m/z (EI) calcd for $C_{19}H_{19}NO_2$ 293.1416, found 293.1411.

4-(4-Chlorophenyl)-1,1,6-trimethyl-1,2,3,4-tetrahydro-5*H*-benzo[def]furo[3,2-*b*]carbazol-5-one (**11a**). A 10 mL sealed tube was charged with Tan-IIA (0.1 mmol), and 4-chloroaniline (0.11 mmol). PhMe (1 mL) was added, and the resulting mixture was stirred at 120 °C. The reaction was monitored by TLC until the starting material disappeared. The solvent was removed *in vacuo,* and the residue was purified using a silica gel column with CH_2Cl_2 as the eluent to give the product **11a** as yellow solids in 70% yield. ^1H NMR (300 MHz, $CDCl_3$) δ 7.91 (d, J = 7.2 Hz, 1H), 7.64-7.46 (m, 4H), 7.31 (s, 1H), 7.18 (d, J = 7.2 Hz, 1H), 3.04 (t, J = 6.0 Hz, 2H), 2.48 - 2.35 (m, 3H), 2.12 (t, J = 6.0 Hz, 2H), 1.50 (s, 6H). ^{13}C NMR (126 MHz, $CDCl_3$) δ 170.97, 155.04, 147.03,

140.83, 140.78, 136.22, 135.53, 130.04(2C), 128.32(2C), 126.35, 125.35, 124.37, 122.49, 121.44, 121.28, 117.03, 115.17, 41.45, 35.65, 28.68(2C), 22.03, 10.28. HRMS m/z (EI) calcd for $C_{25}H_{20}ClNO_2$ 401.1183, found 401.1182.

4-Benzyl-1,1,6-trimethyl-1,2,3,4-tetrahydro-5*H*-benzo[*def*]furo[3,2-*b*]carbazol-5-one (**11b**). A 10 mL sealed tube was charged with Tan-IIA (0.1 mmol), and benzylamine (0.11 mmol). PhMe (1 mL) was added, and the resulting mixture was stirred at 120 °C. The reaction was monitored by TLC until the starting material disappeared. The solvent was removed *in vacuo,* and the residue was purified using a silica gel column with CH_2Cl_2 as the eluent to give the product **11b** as yellow solids in 65% yield. ^{1}H NMR (300 MHz, $CDCl_3$) δ 7.88 (d, J = 7.5 Hz, 1H), 7.39- 7.26 (m, 6H), 7.14 (d, J = 7.2 Hz, 1H), 5.98 (s, 2H), 2.99 (t, J =6.0 Hz, 2H), 2.50 (s, 3H), 2.08 (t, J = 6.3 Hz, 2H), 1.38 (s, 6H). ^{13}C NMR (151 MHz, $CDCl_3$) δ 171.07, 154.51, 153.54, 146.07, 139.97, 136.66, 128.92(2C), 127.92, 127.58(2C), 125.58, 123.80, 123.11, 121.63, 120.49, 120.27, 115.90, 114.12, 50.30, 40.65, 34.89, 28.03(2C), 20.75, 9.68. HRMS m/z (EI) calcd for $C_{26}H_{23}NO_2$ 381.1729, found 381.1727.

5:^{1}H NMR

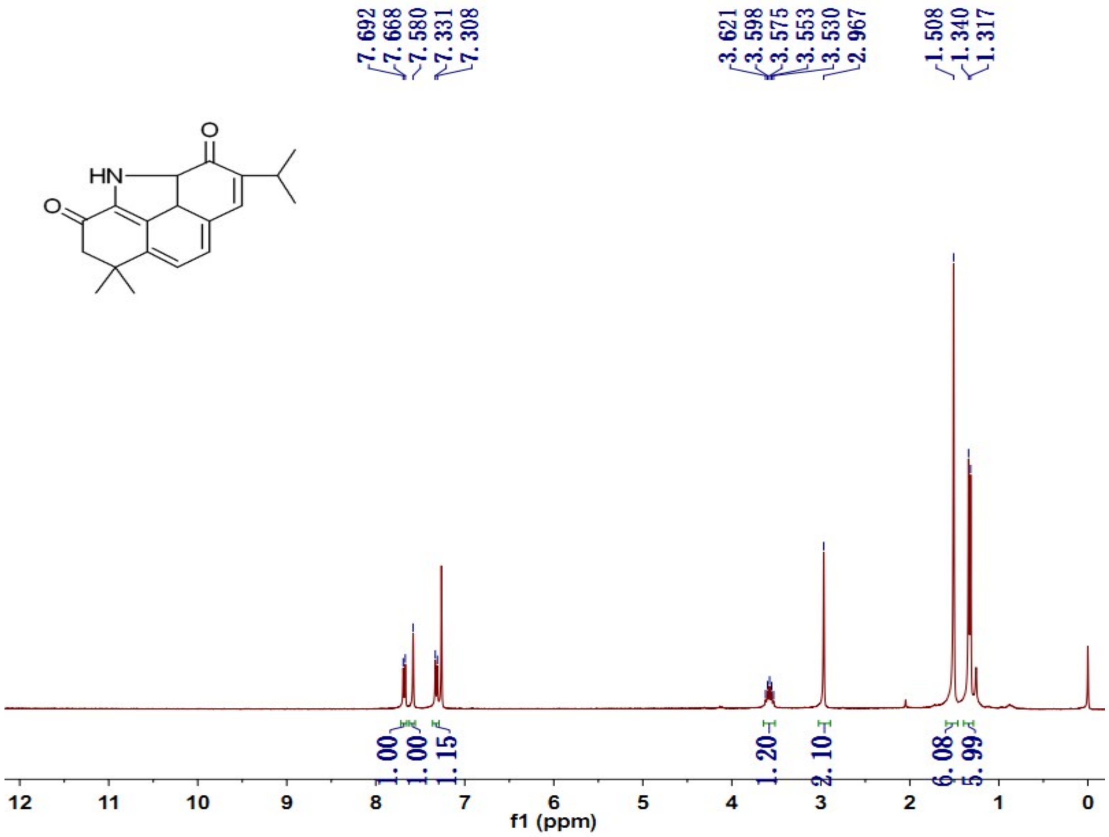

5: ^{13}C NMR

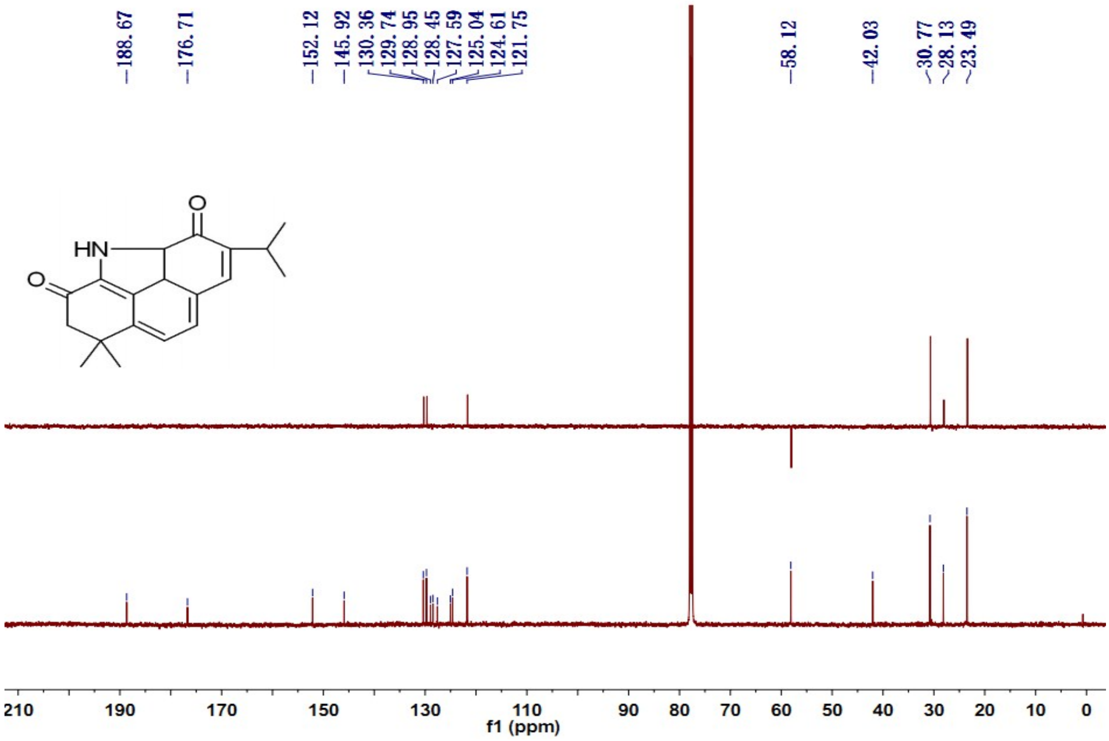

6a :^{1}H NMR

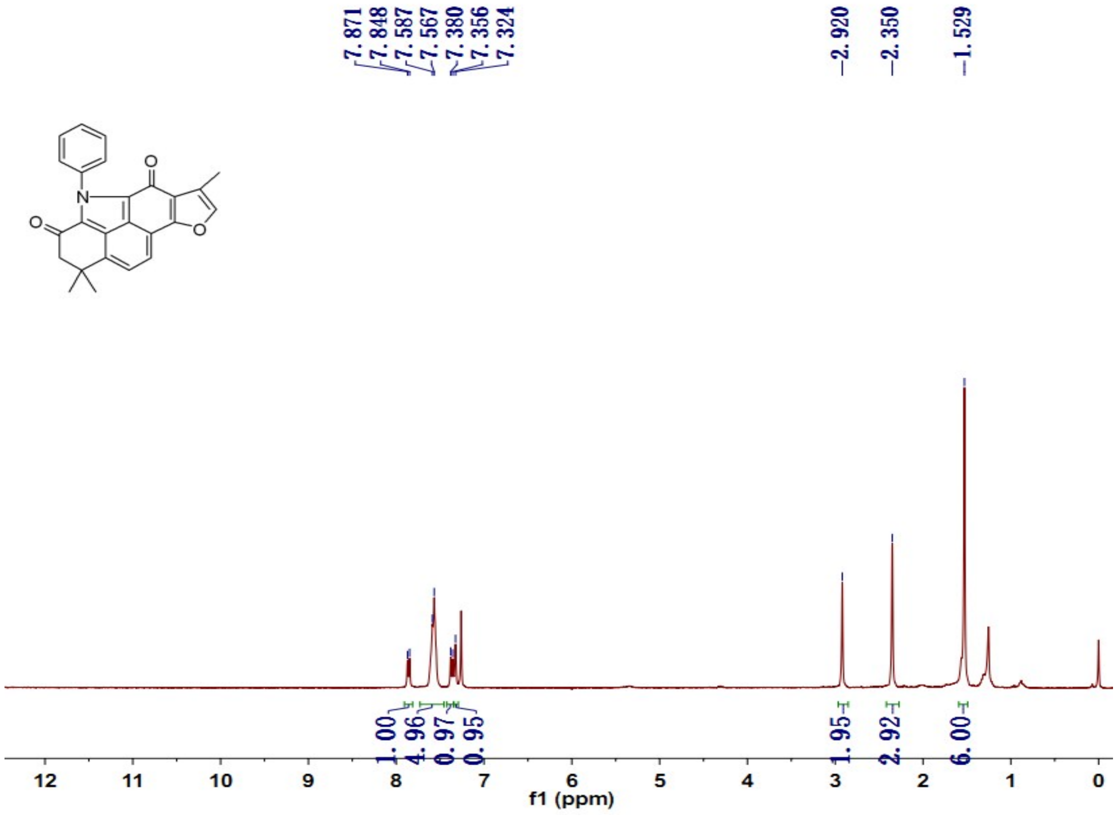

6a: ^{13}C NMR

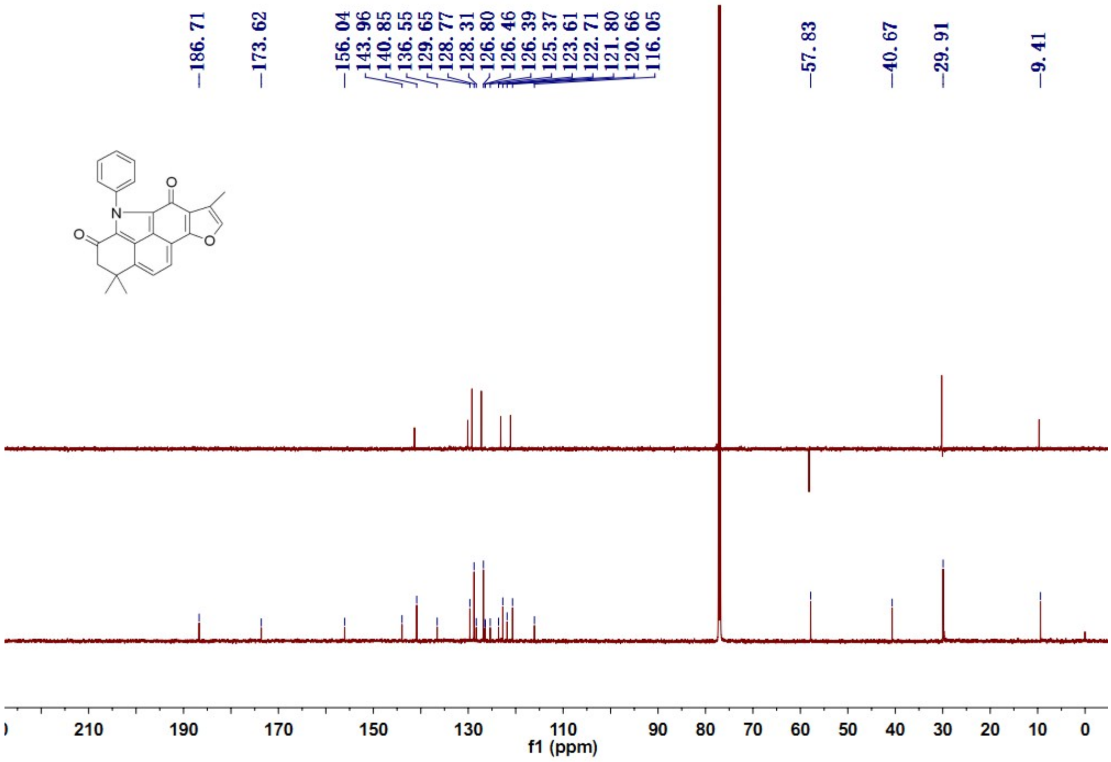

6b: ^{1}H NMR

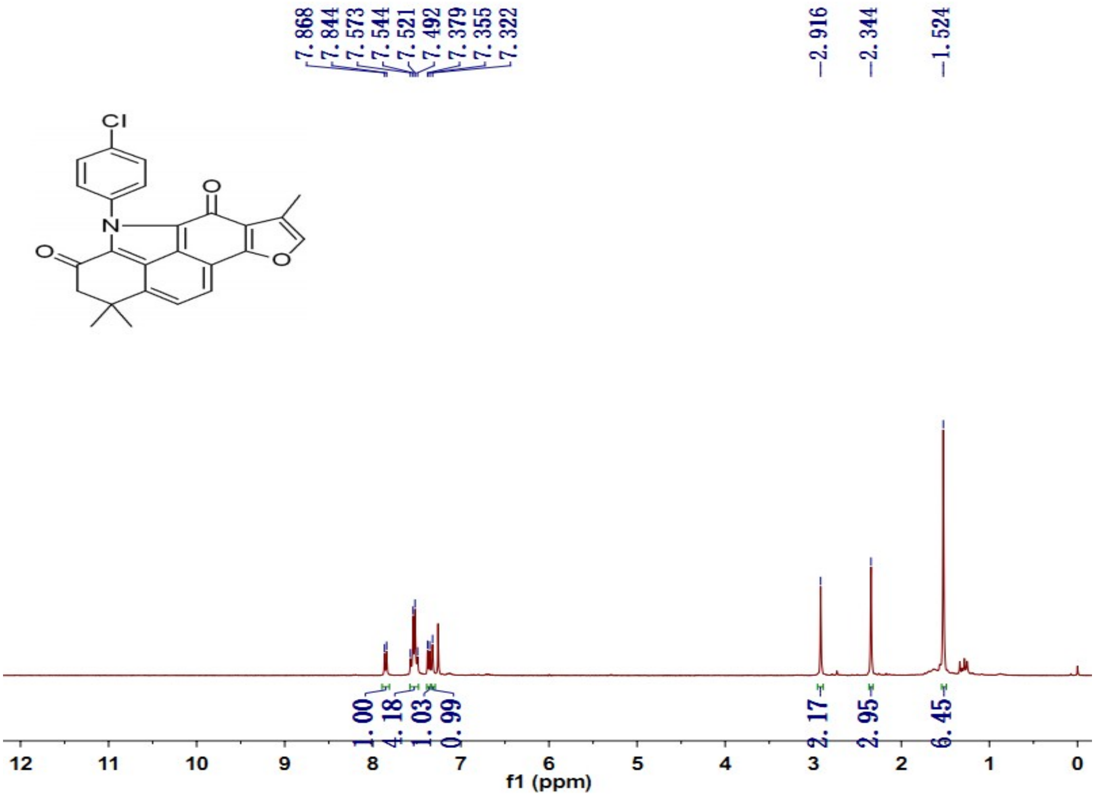

6b: ^{13}C NMR

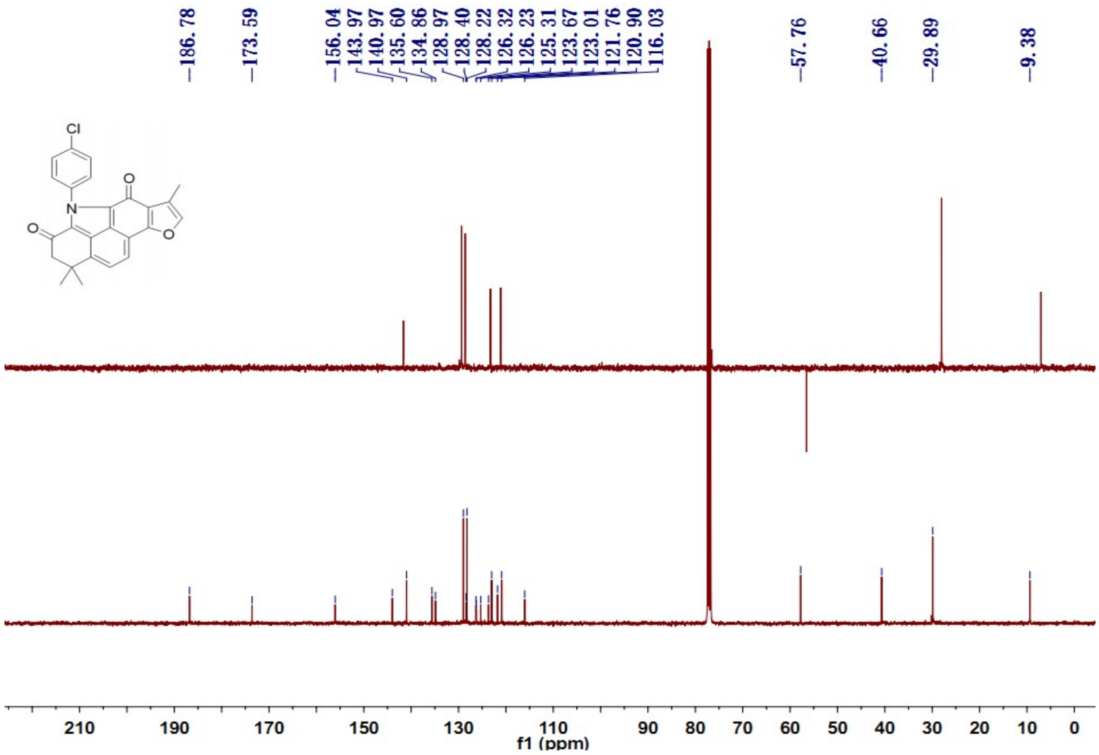

6c: ^{1}H NMR

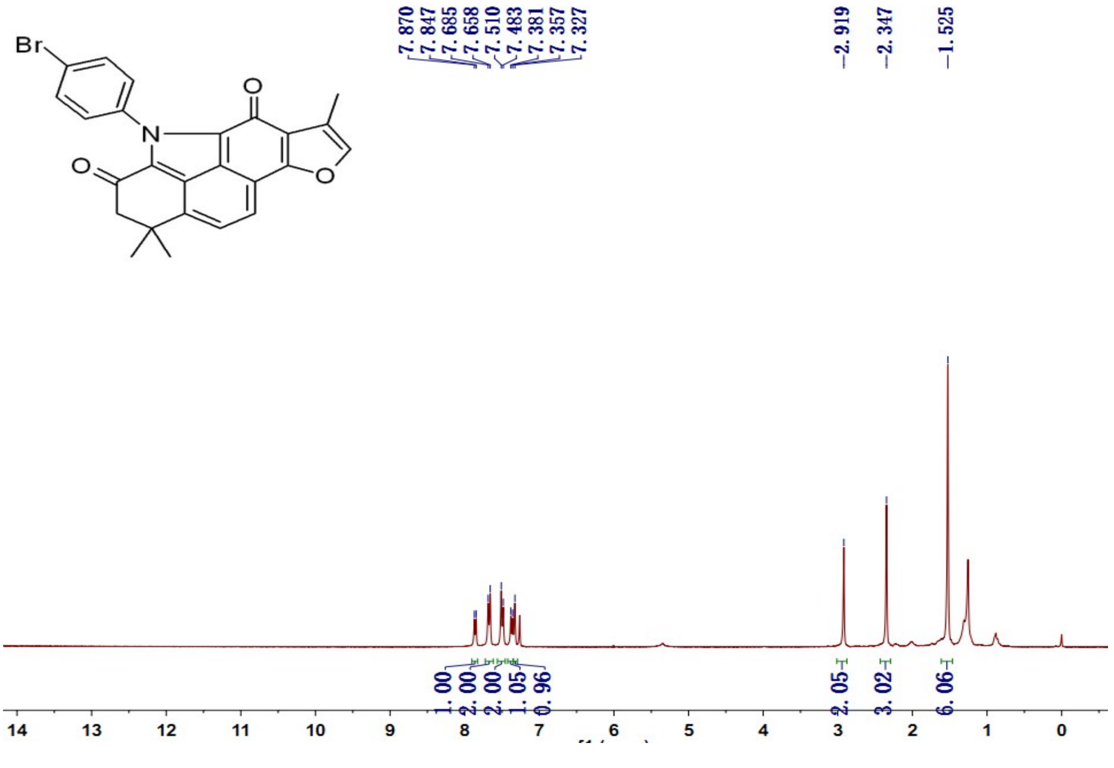

6c: ^{13}C NMR

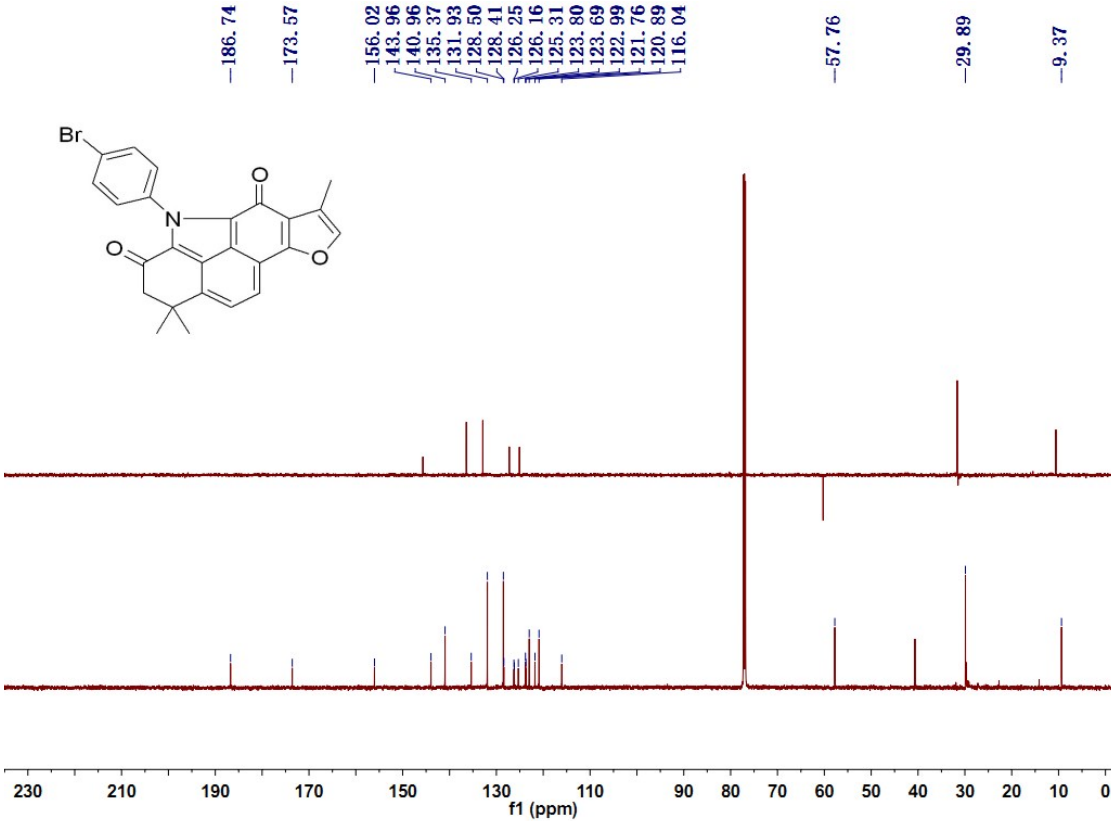

6d :^{1}H NMR

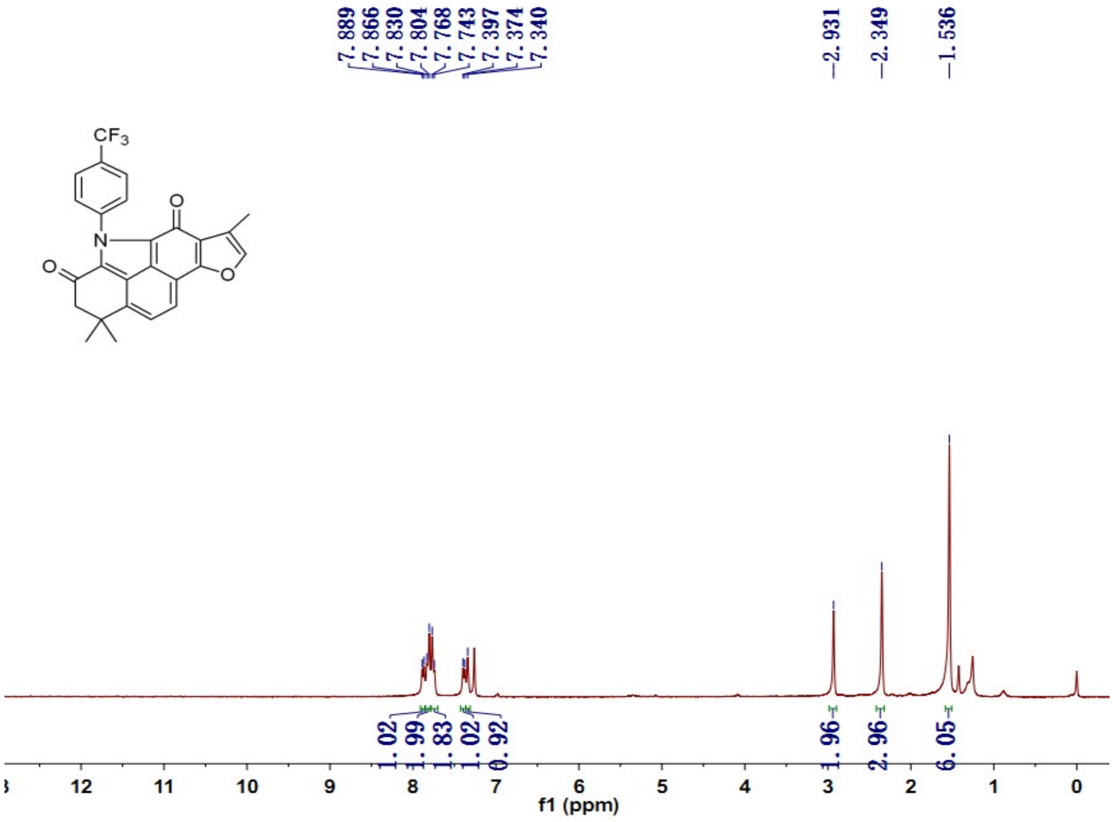

6d: ^{13}C NMR

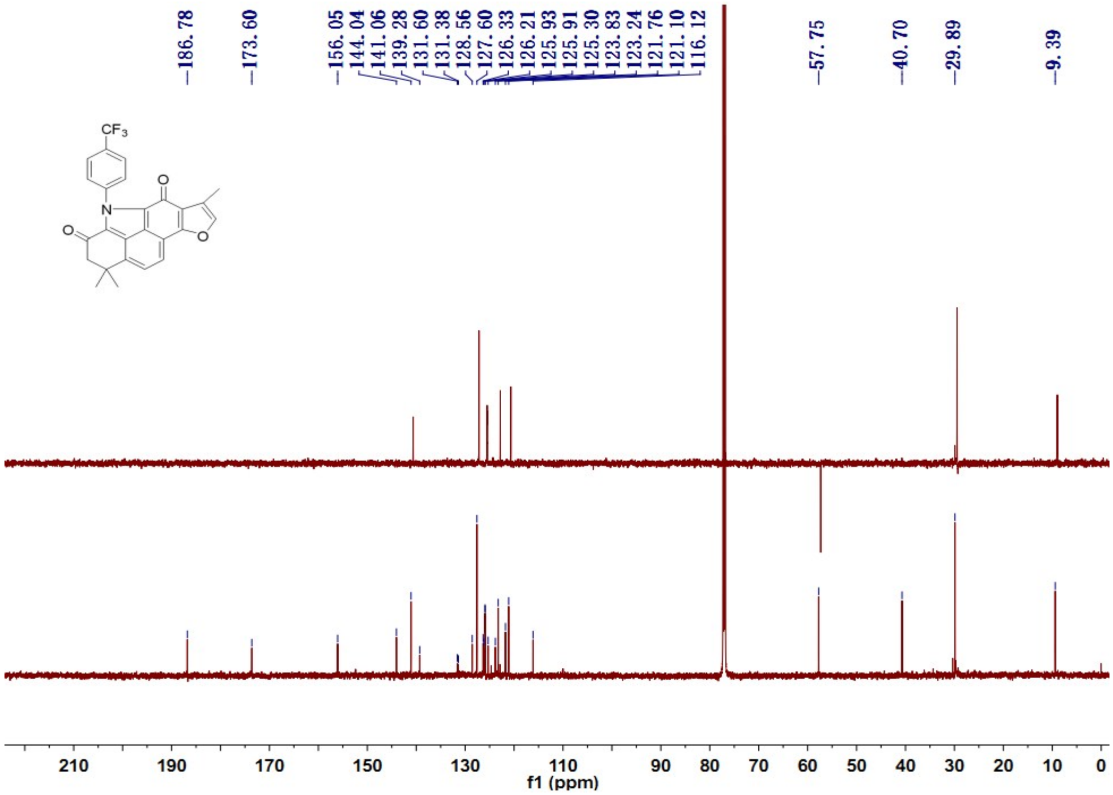

6e: ^{1}H NMR

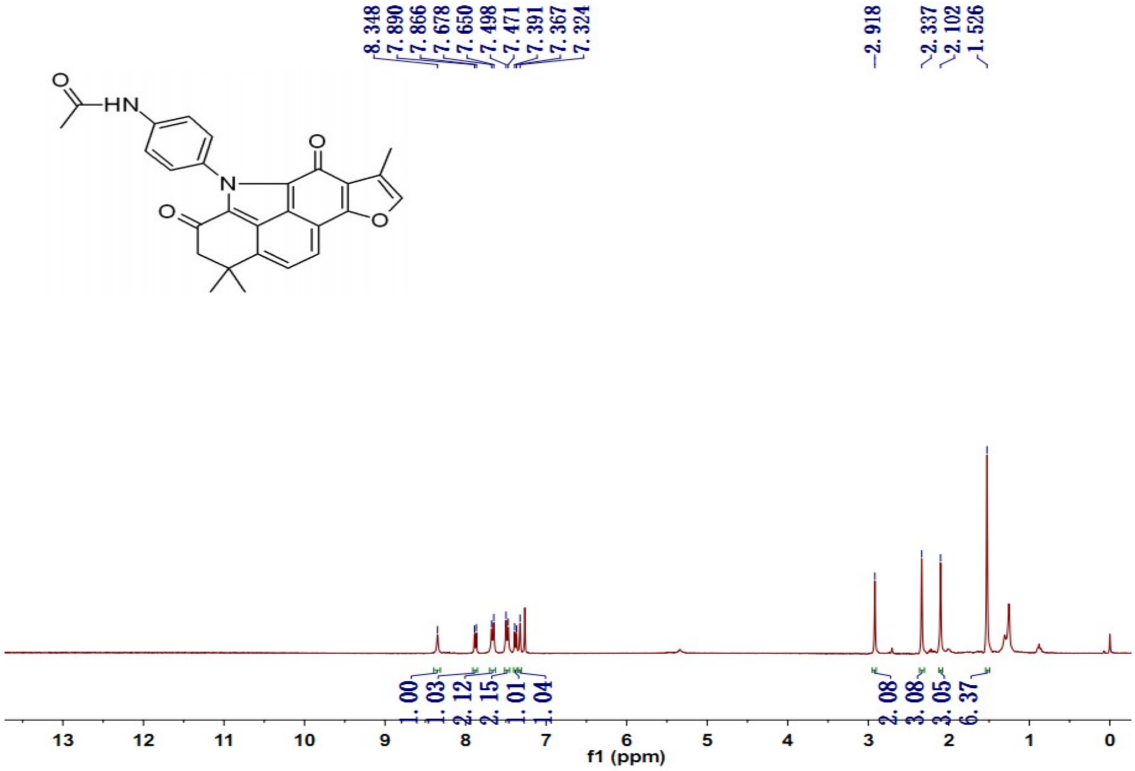

6e: ^{13}C NMR

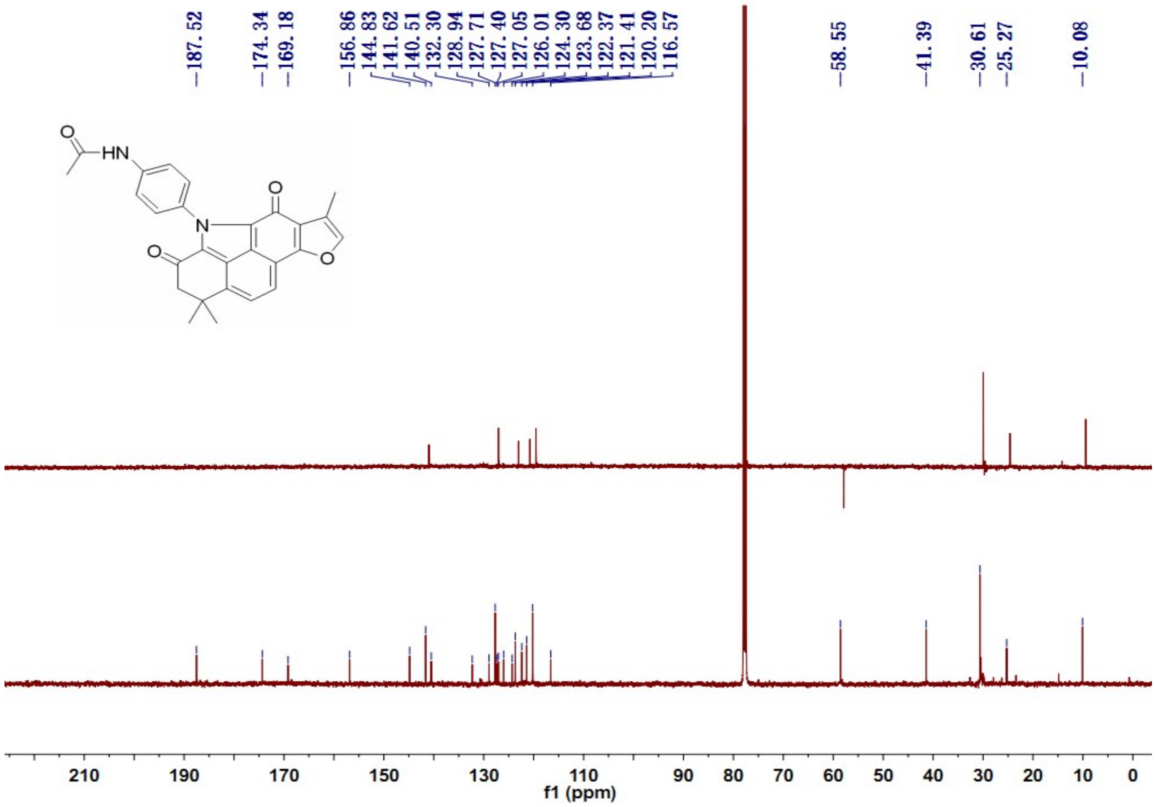

6f : ¹H NMR

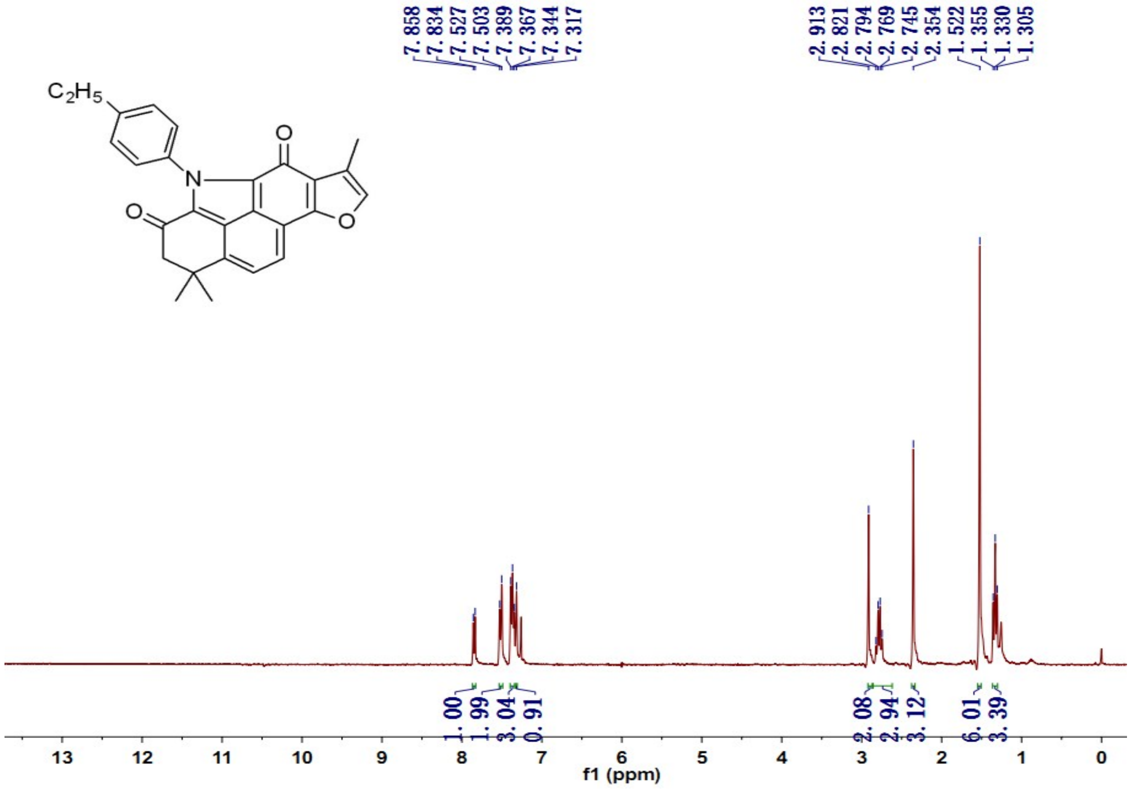

6f: ¹³C NMR

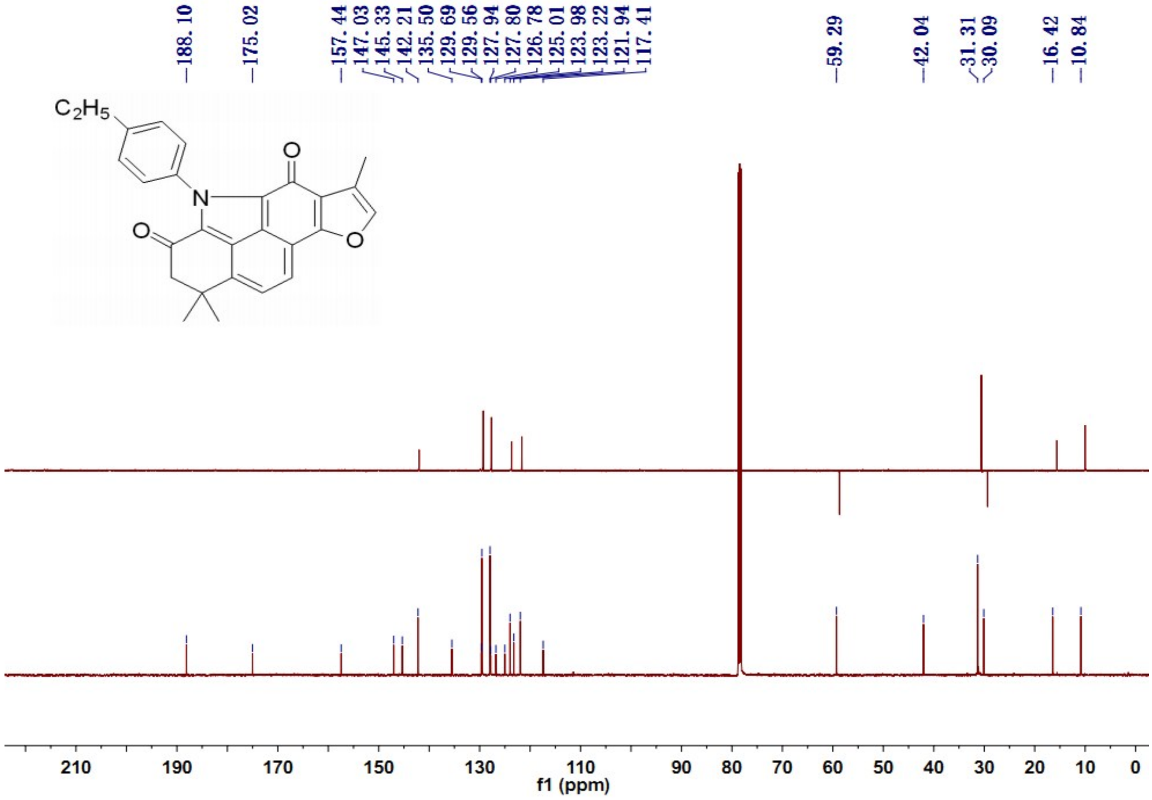

6g: ^{1}H NMR

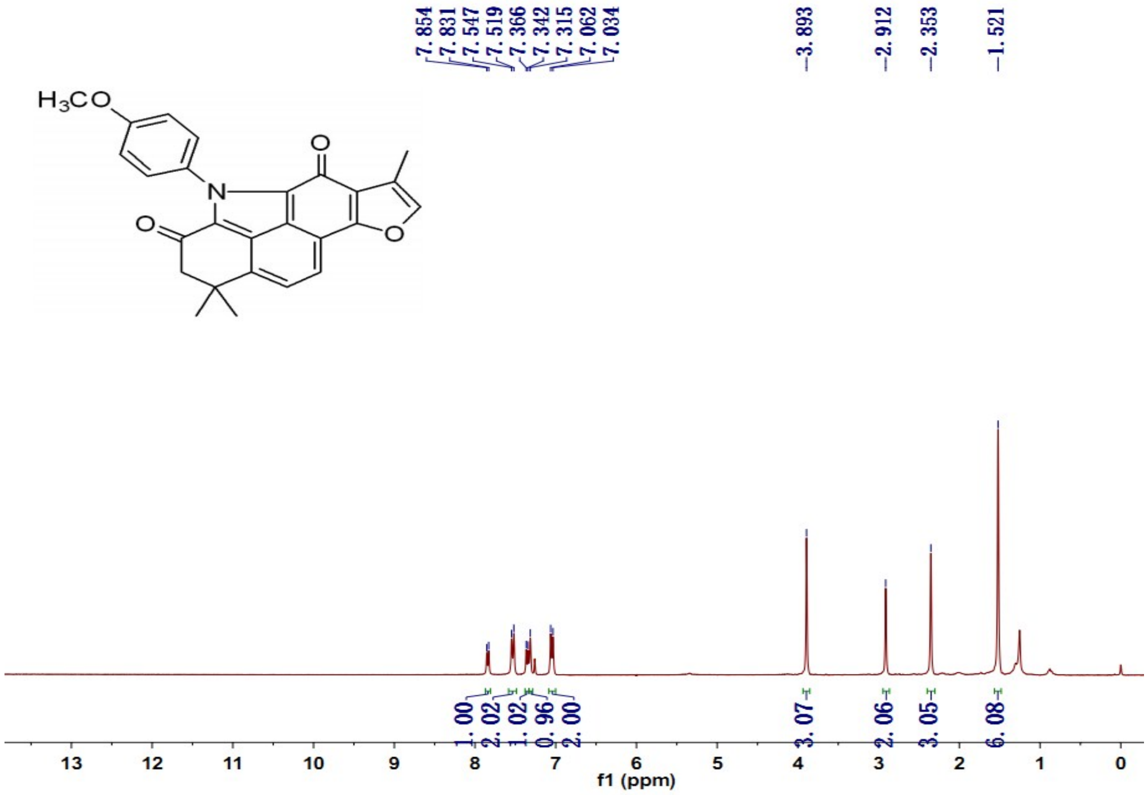

6g: ^{13}C NMR

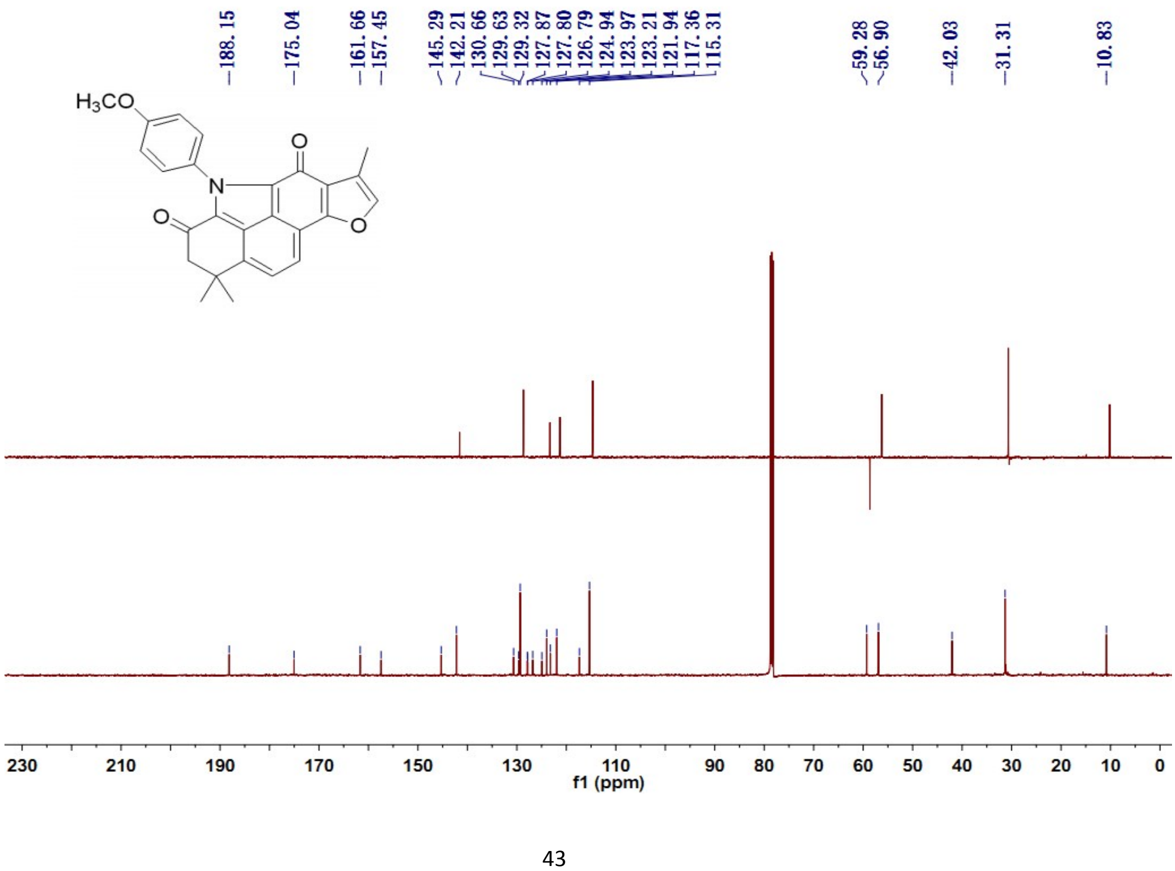

6h: ^{1}H NMR

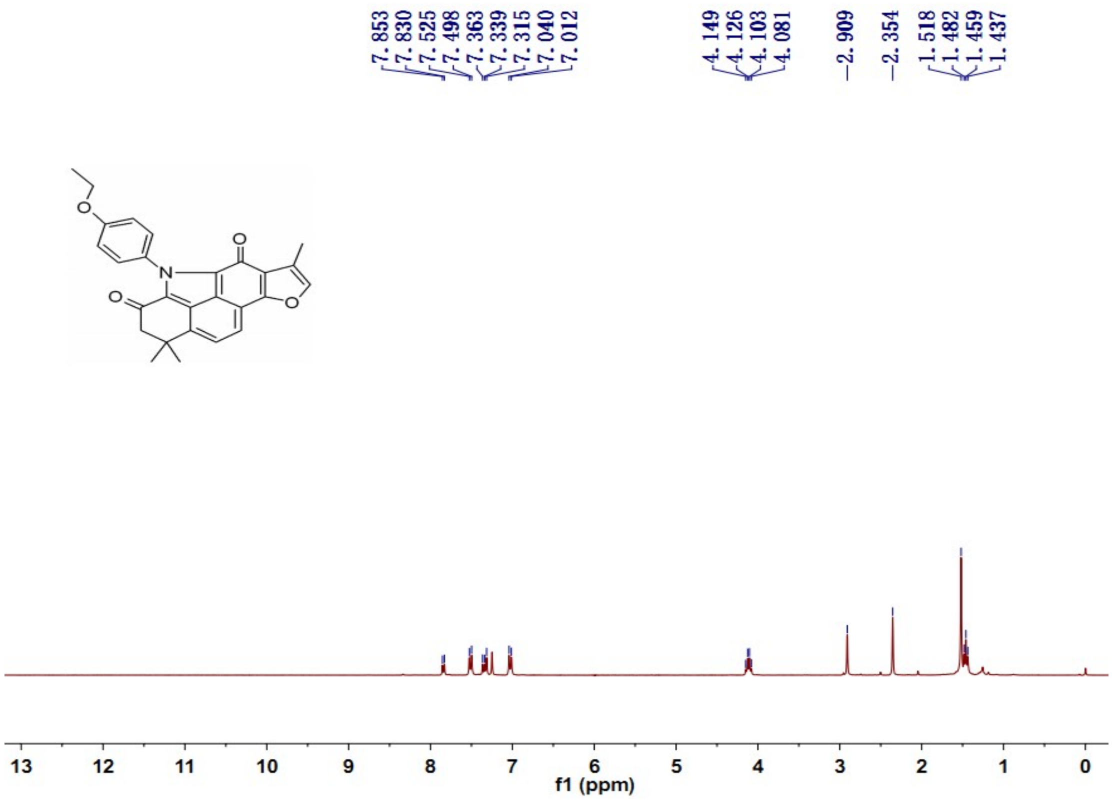

6h: ^{13}C NMR

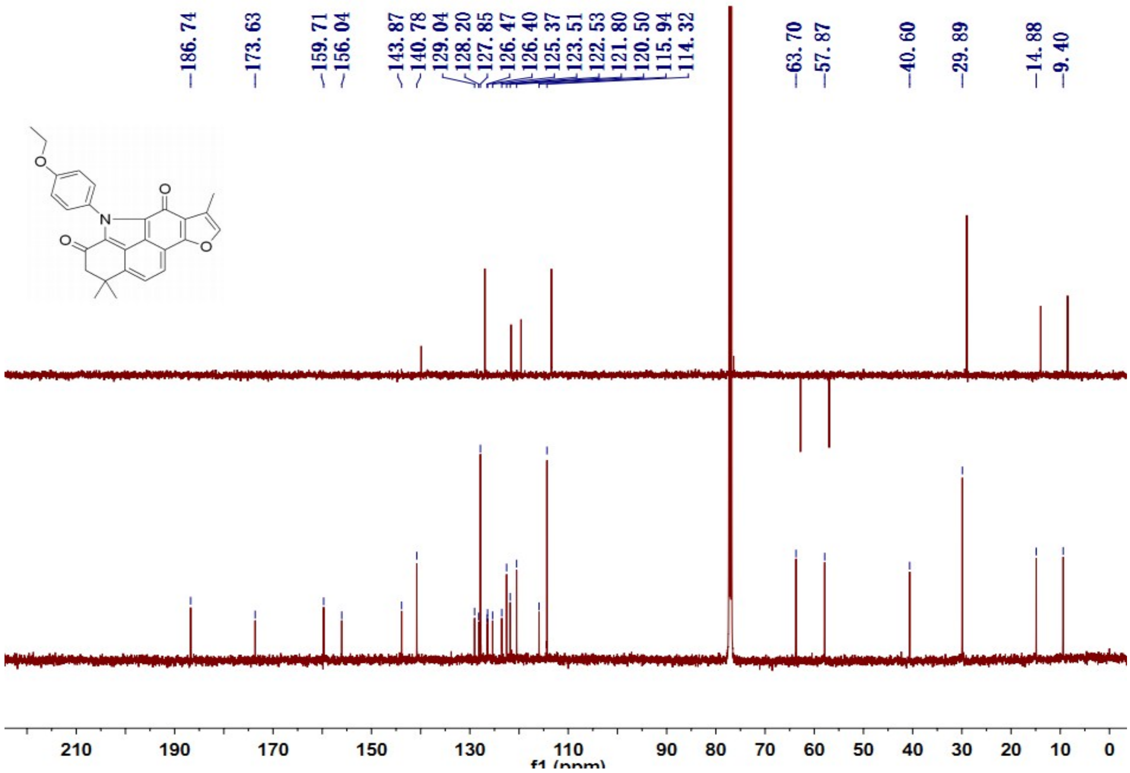

6i: ¹H NMR

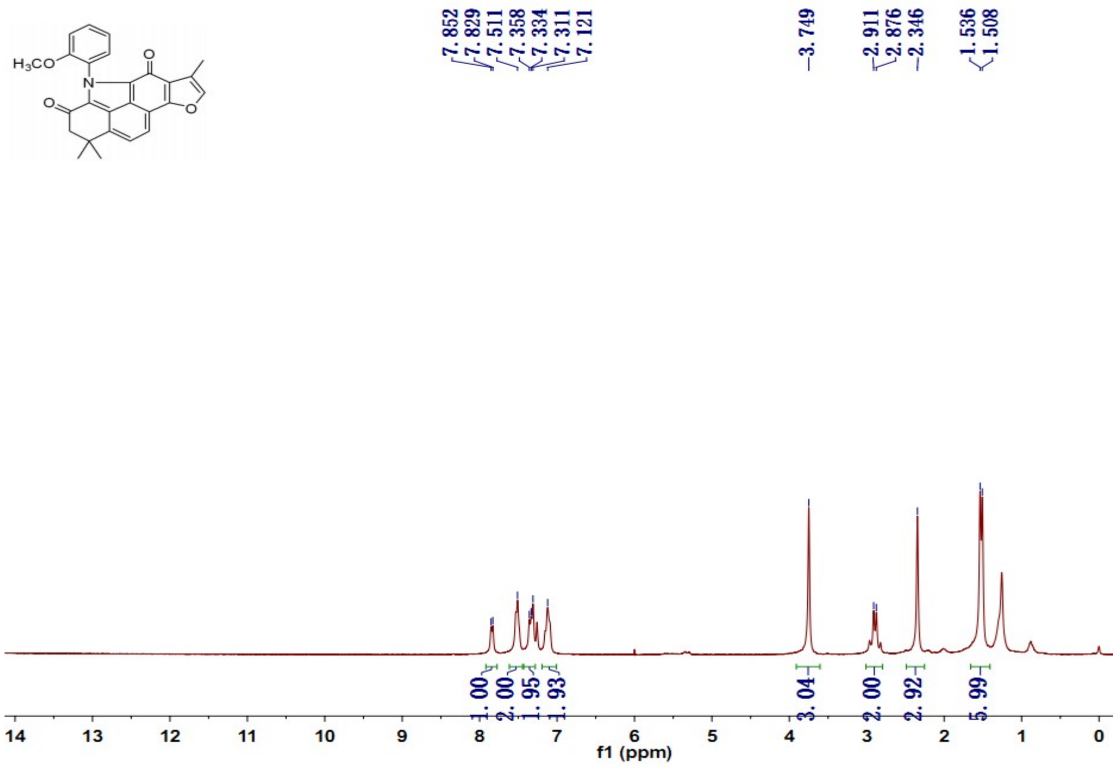

6i: ¹³C NMR

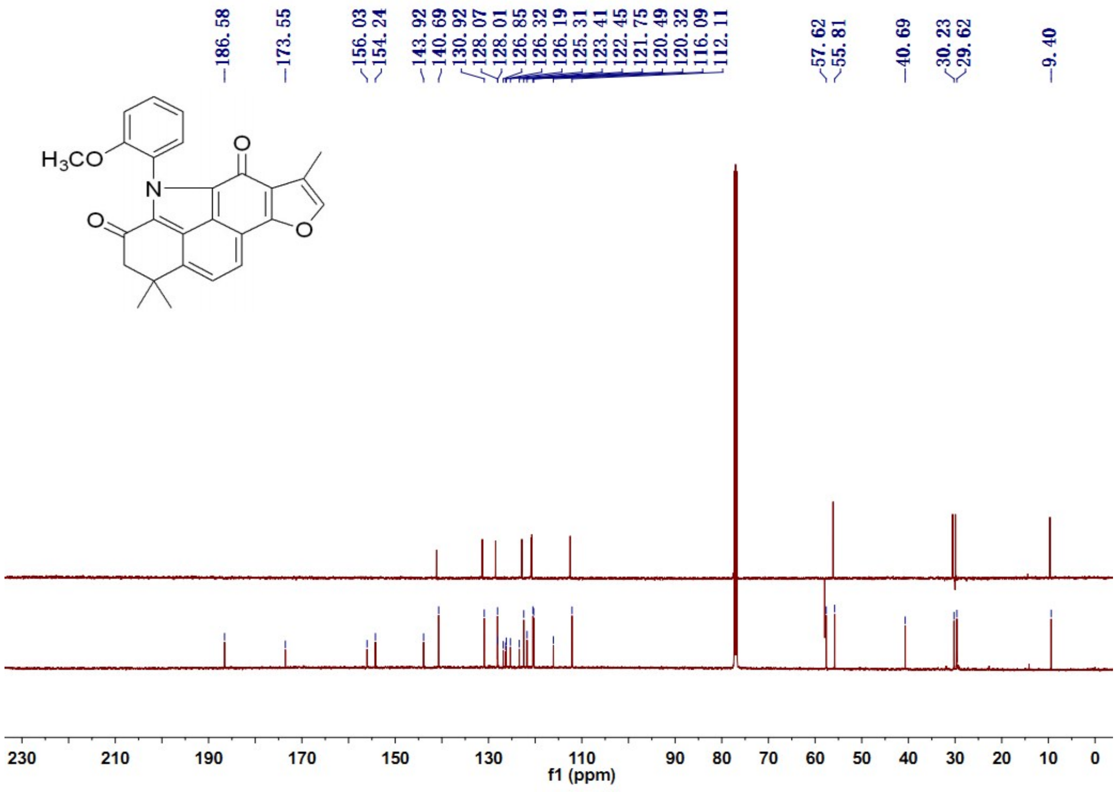

6j: ^{1}H NMR

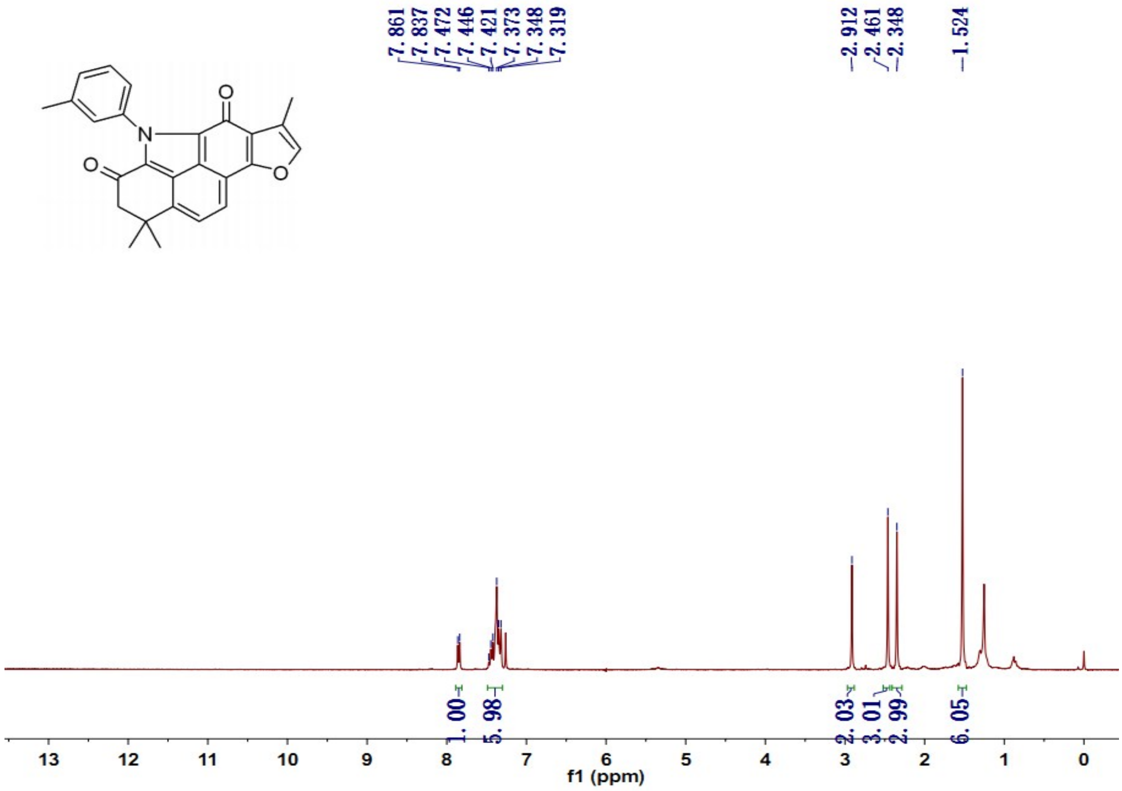

6j: ^{13}C NMR

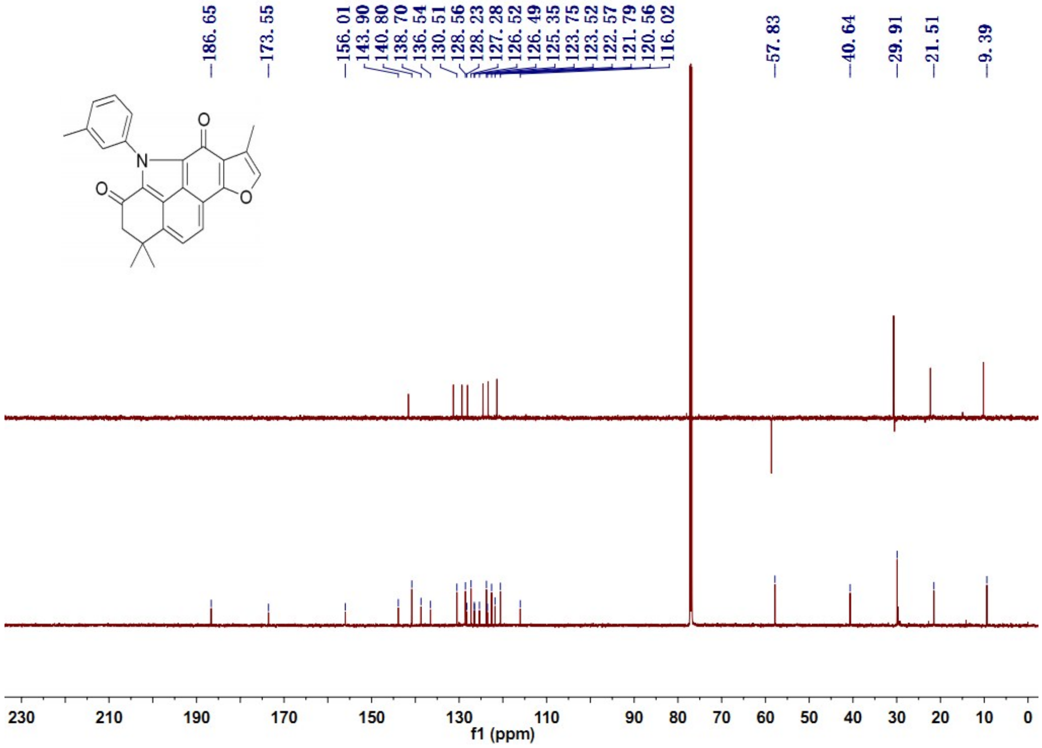

6k: ^{1}H NMR

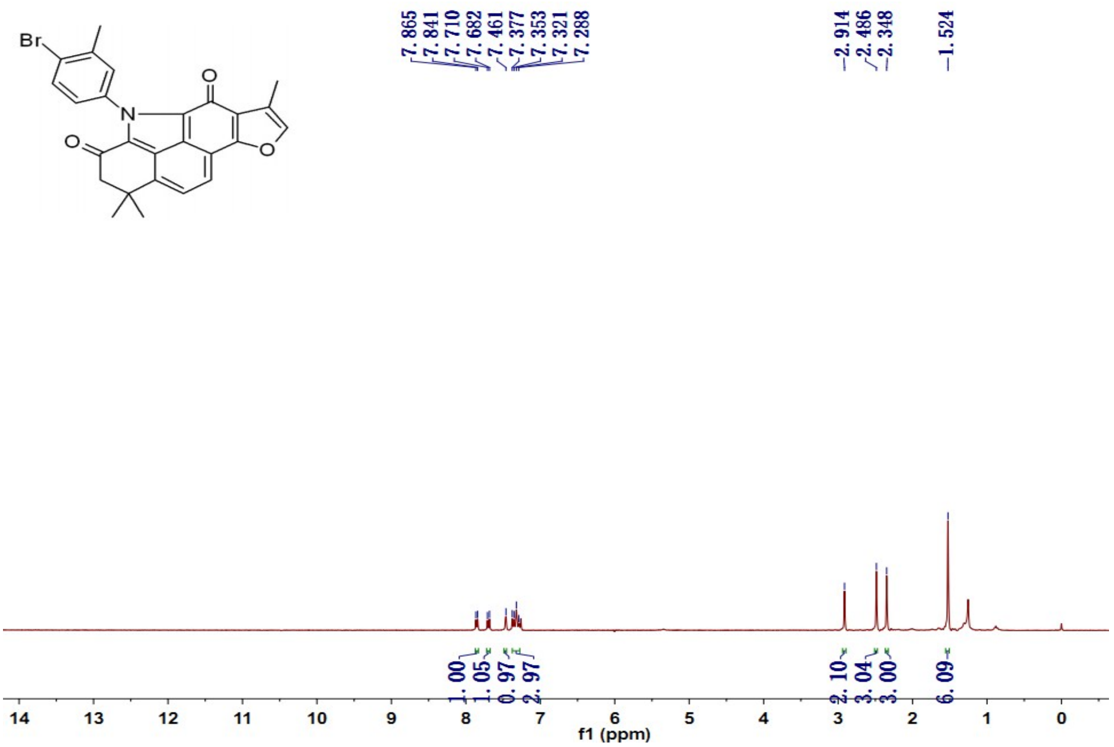

6k: ^{13}C NMR

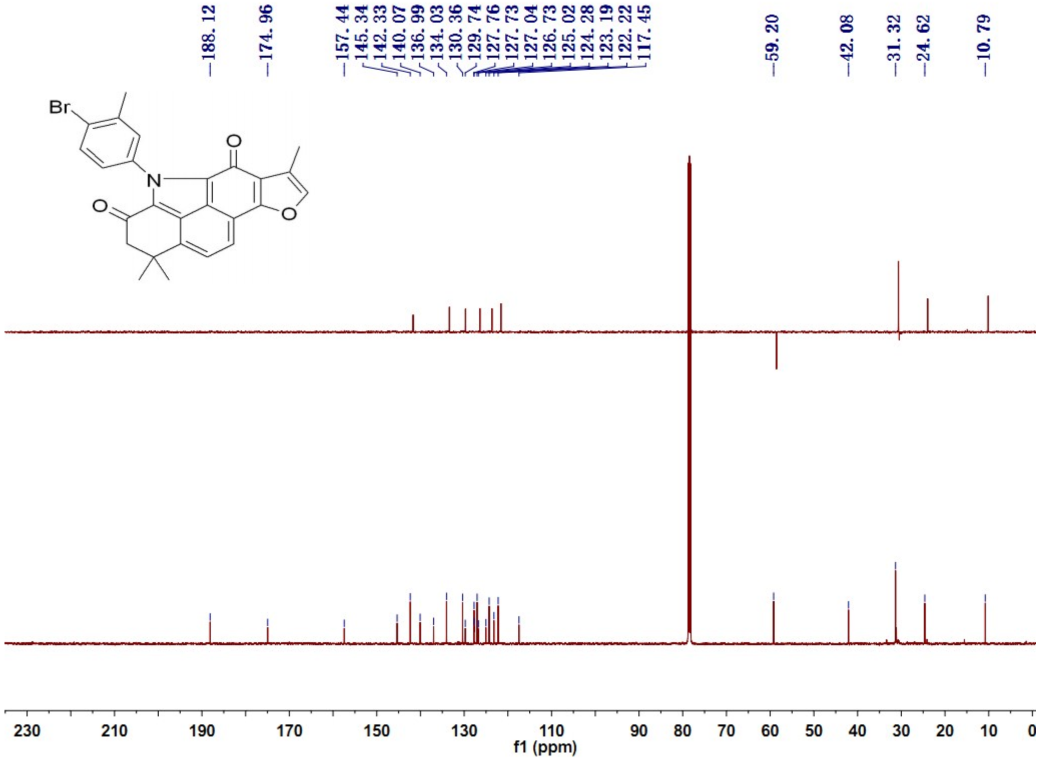

6l: ^{1}H NMR

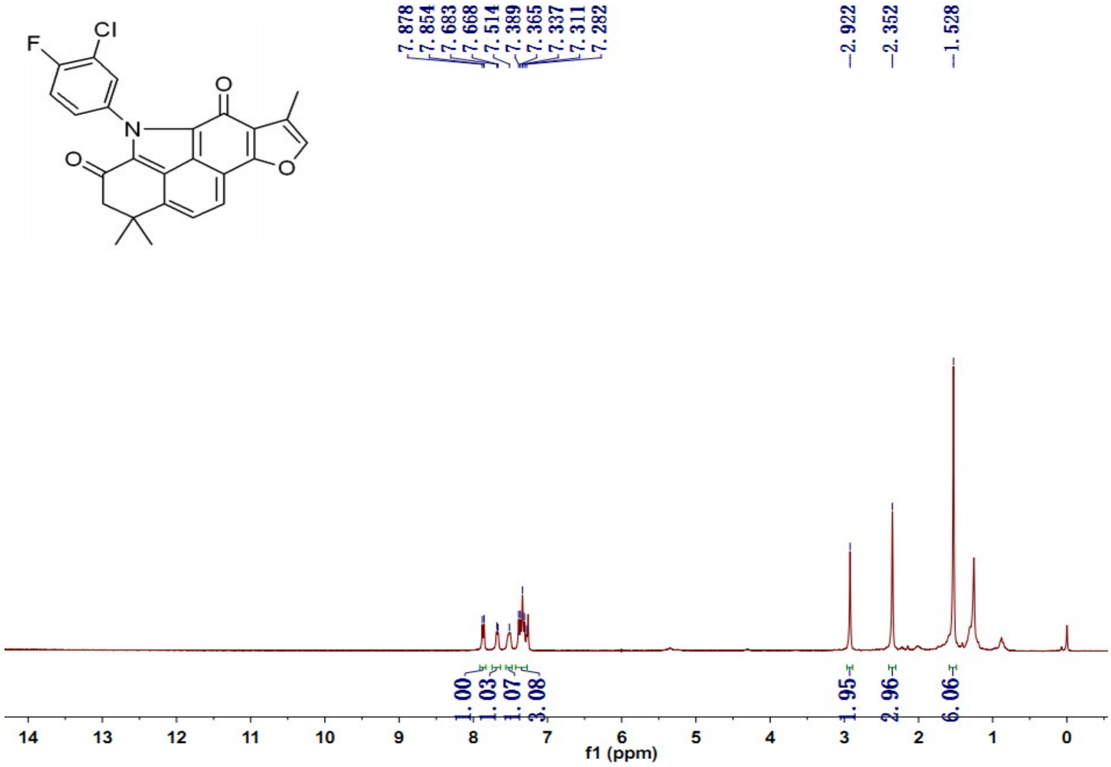

6l: ^{13}C NMR

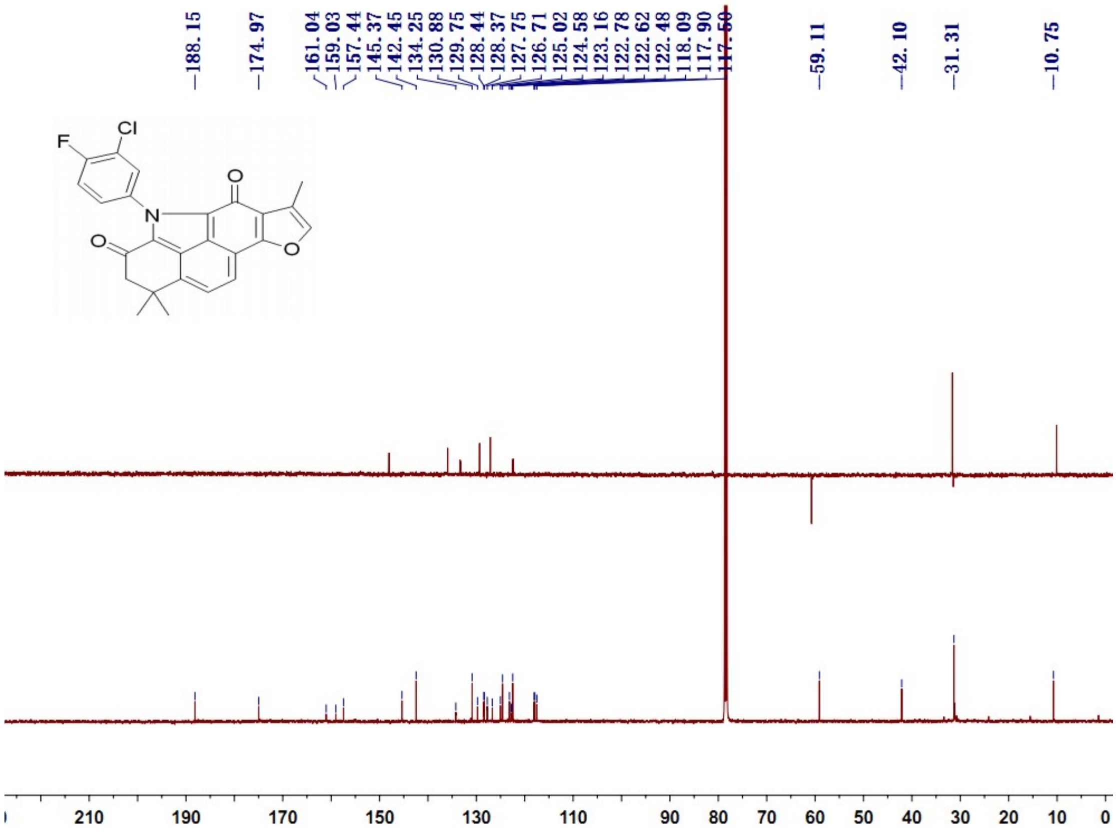

6m: ^{1}H NMR

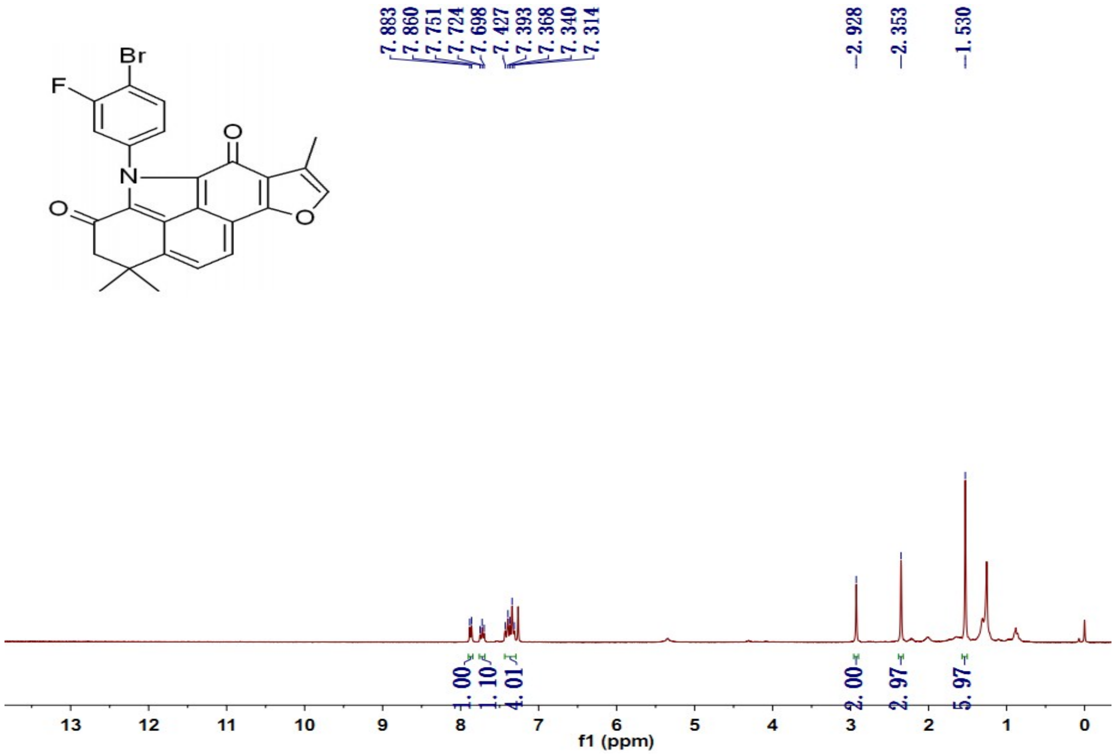

6m: ^{13}C NMR

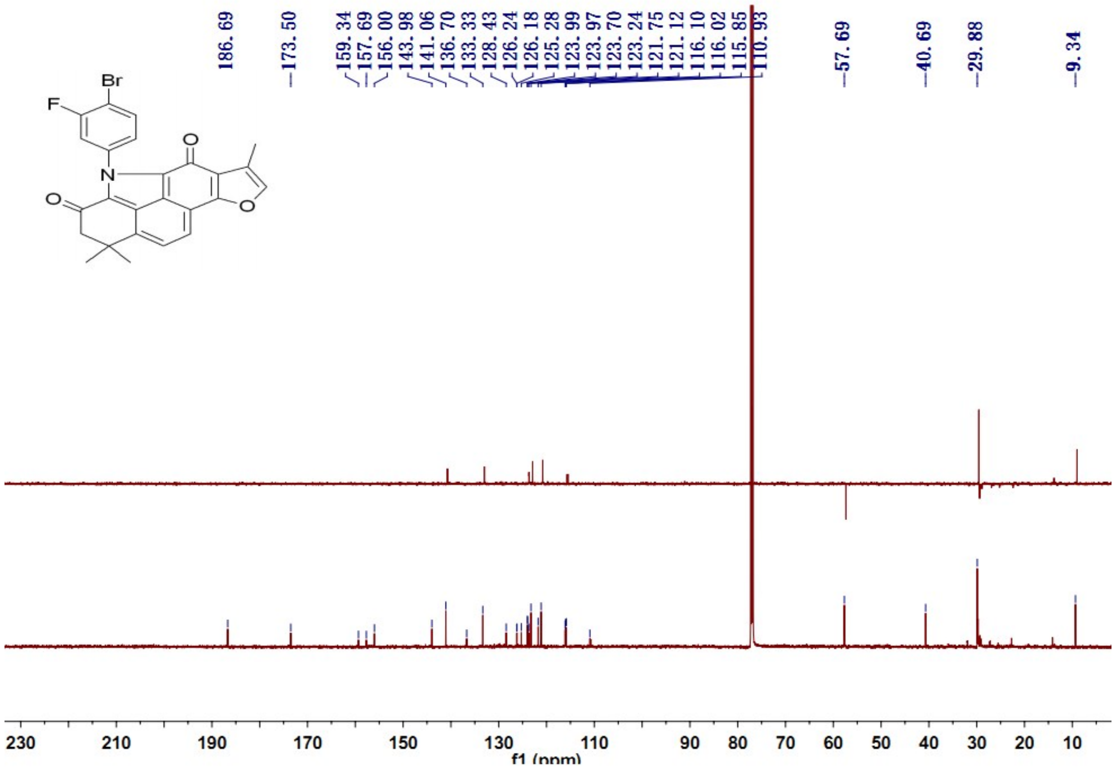

6n: ^{1}H NMR

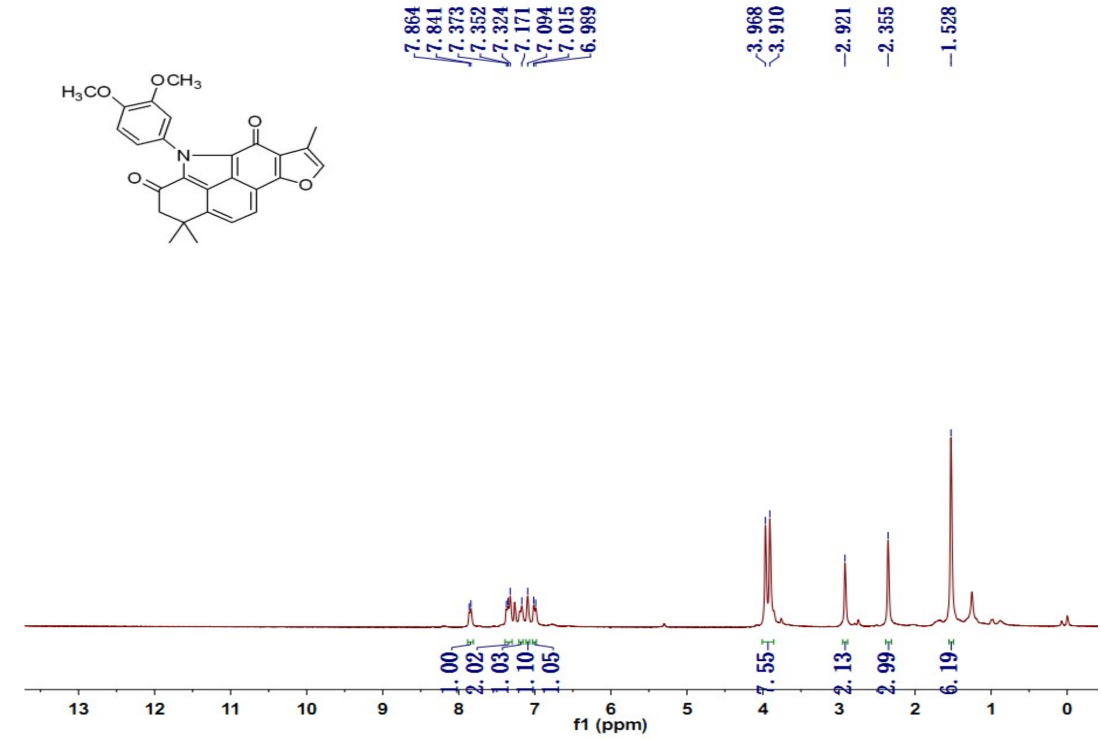

6n: ^{13}C NMR

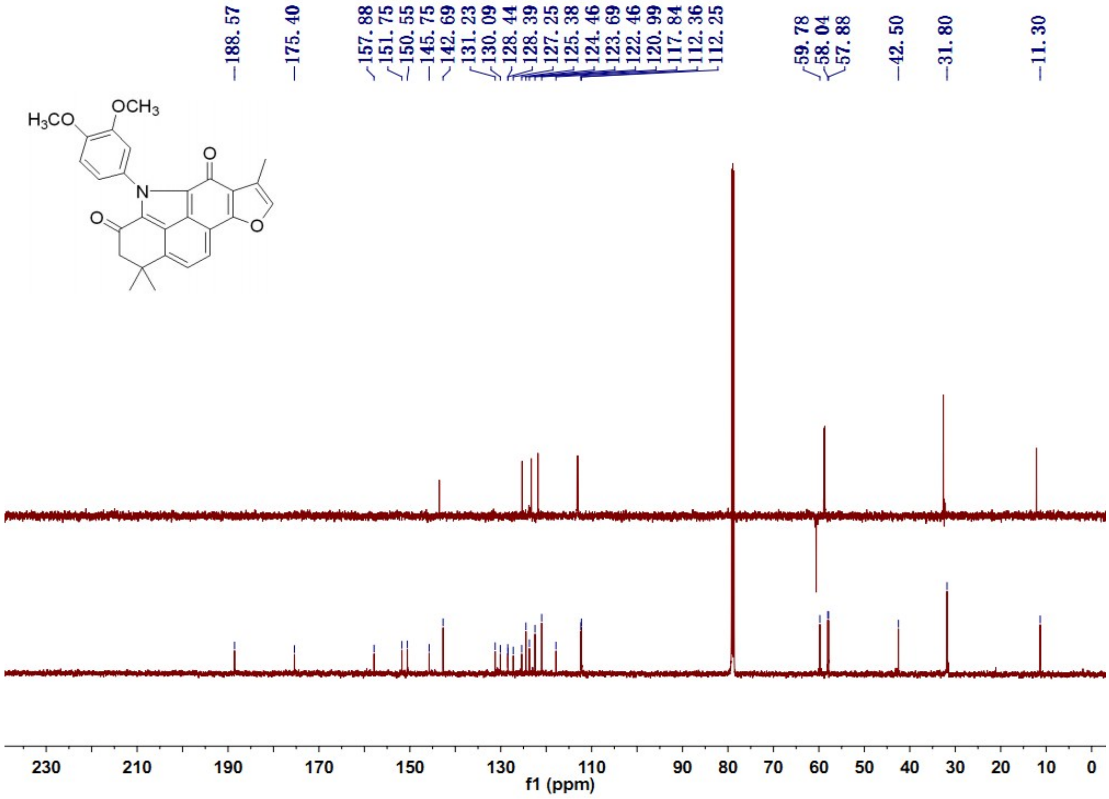

12: ¹H NMR

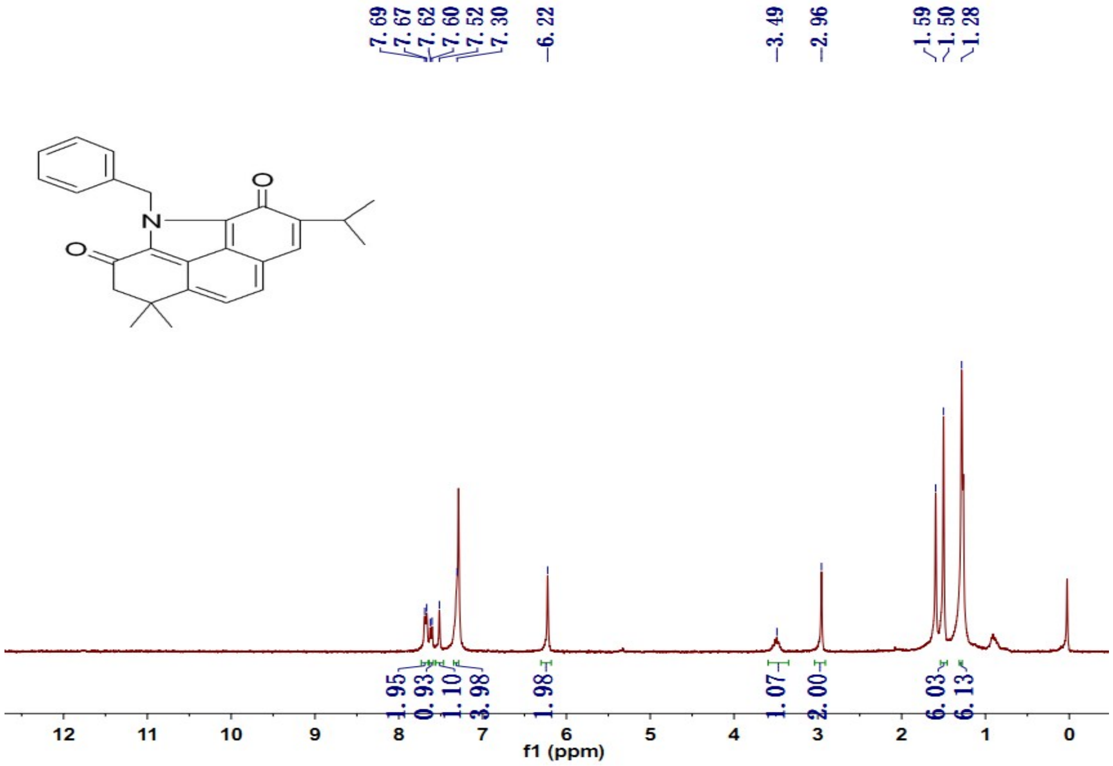

12: ¹³C NMR

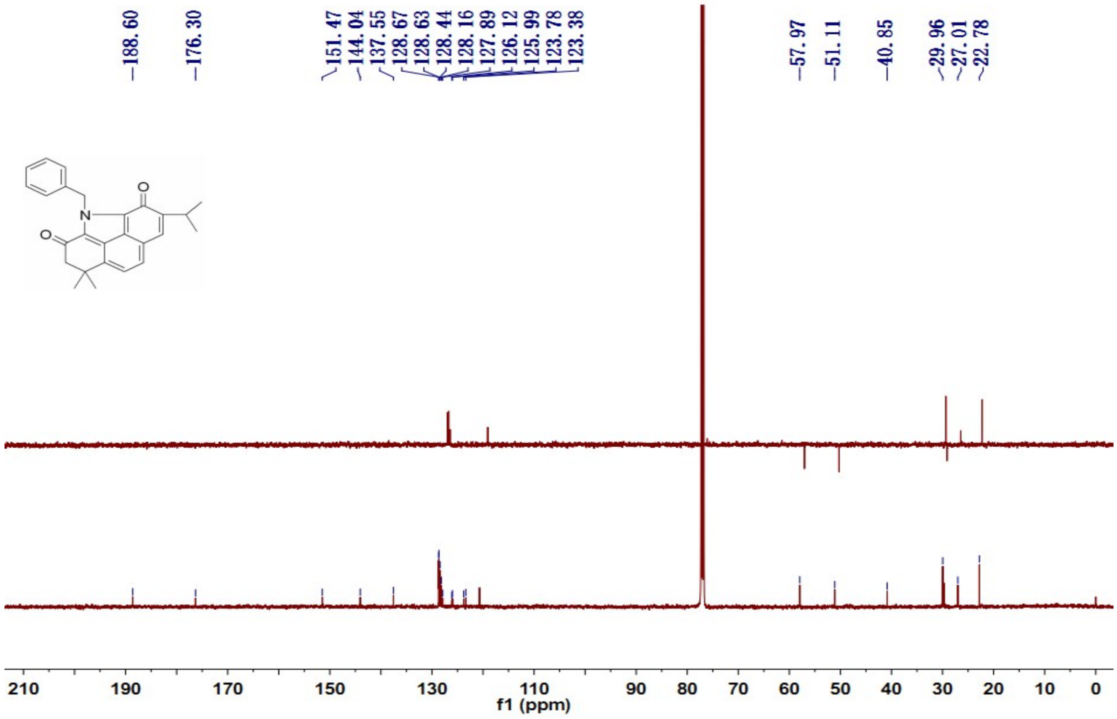

13:^{1}H NMR

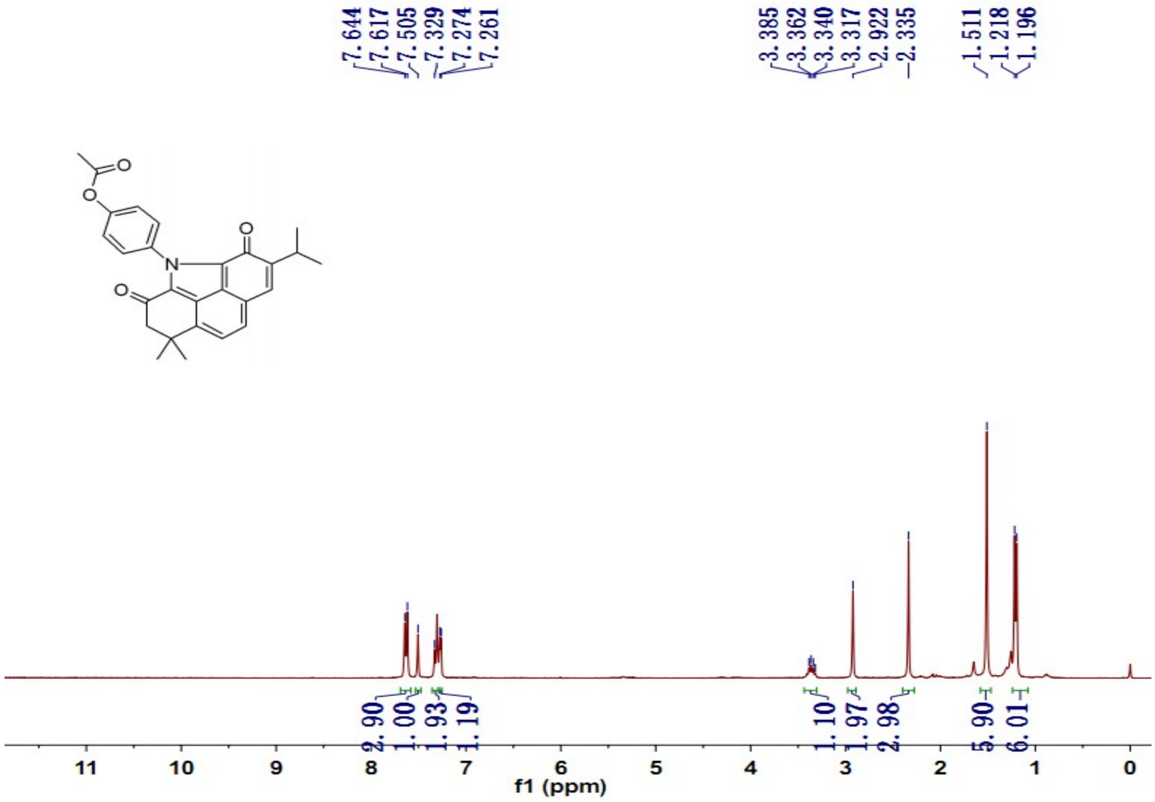

13: ^{13}C NMR

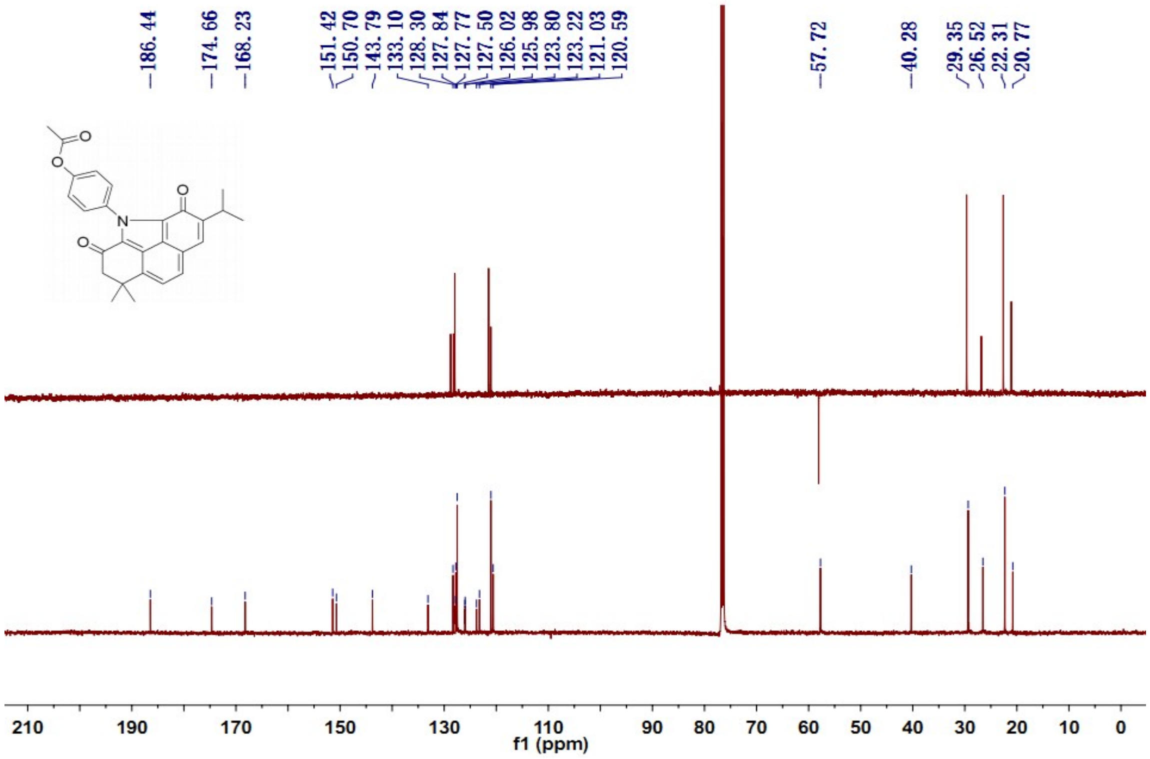

14: ^{1}H NMR

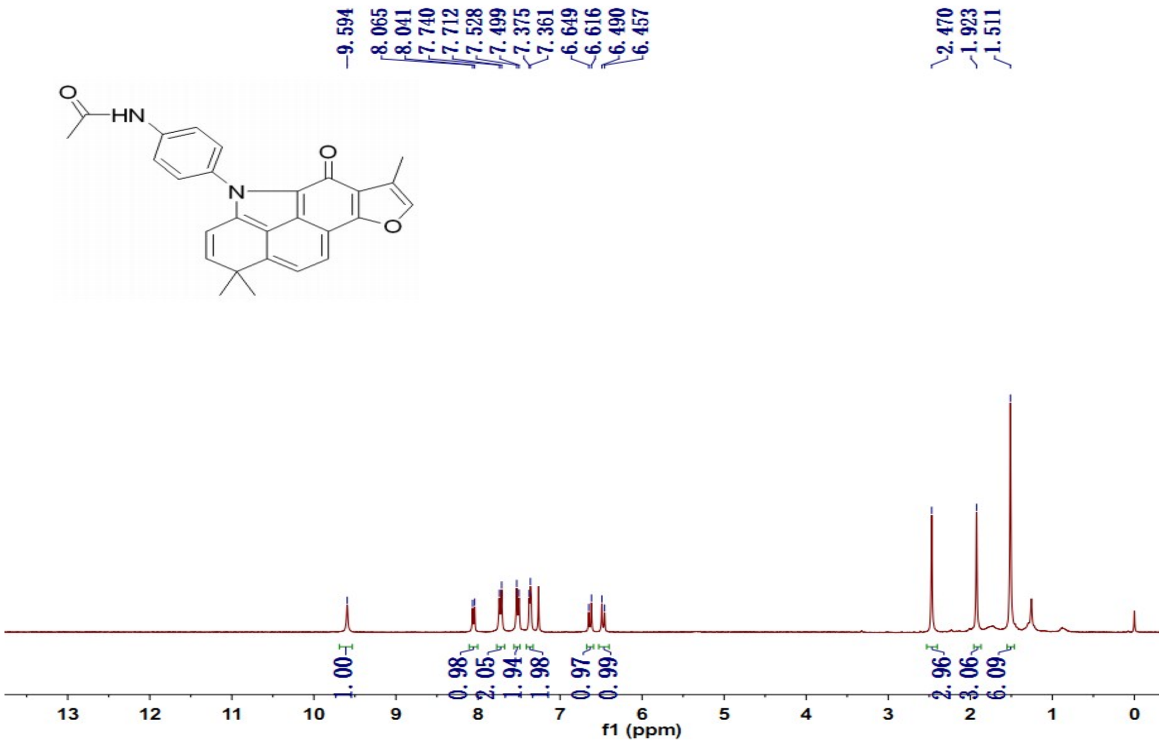

14: ^{13}C NMR

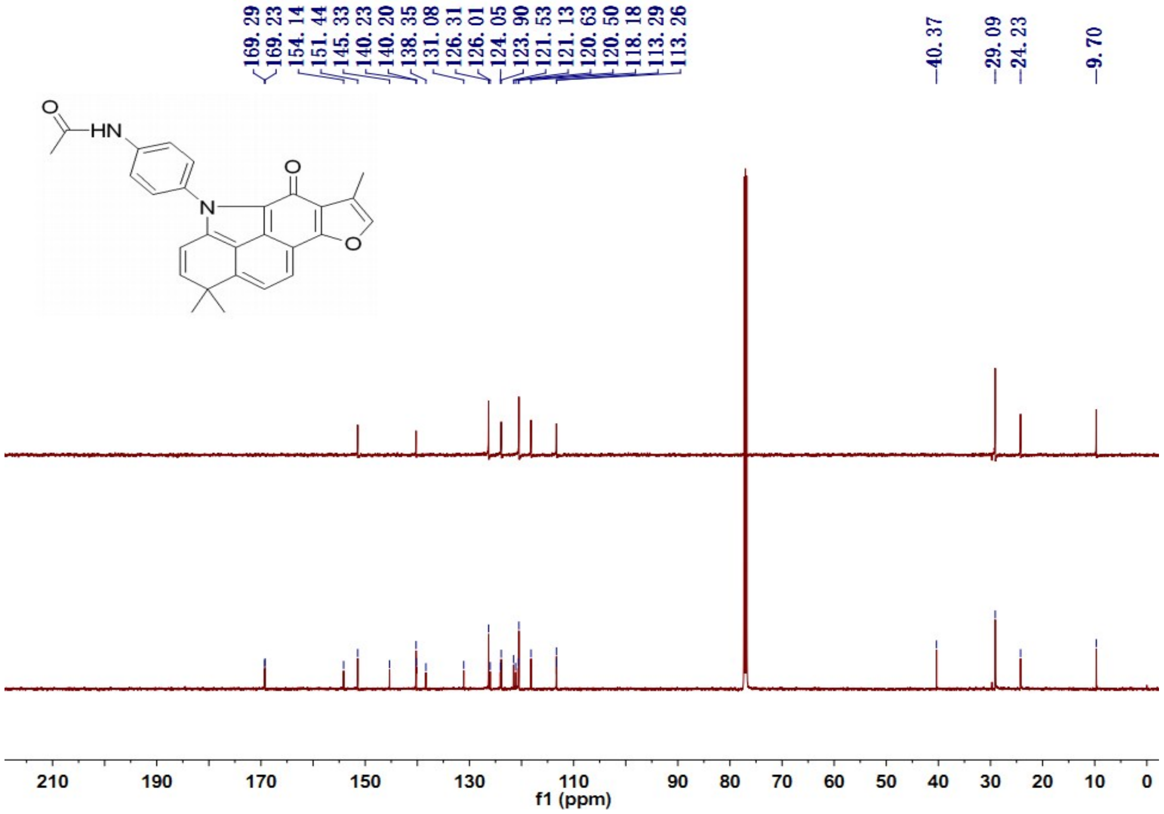

15a:^{1}H NMR

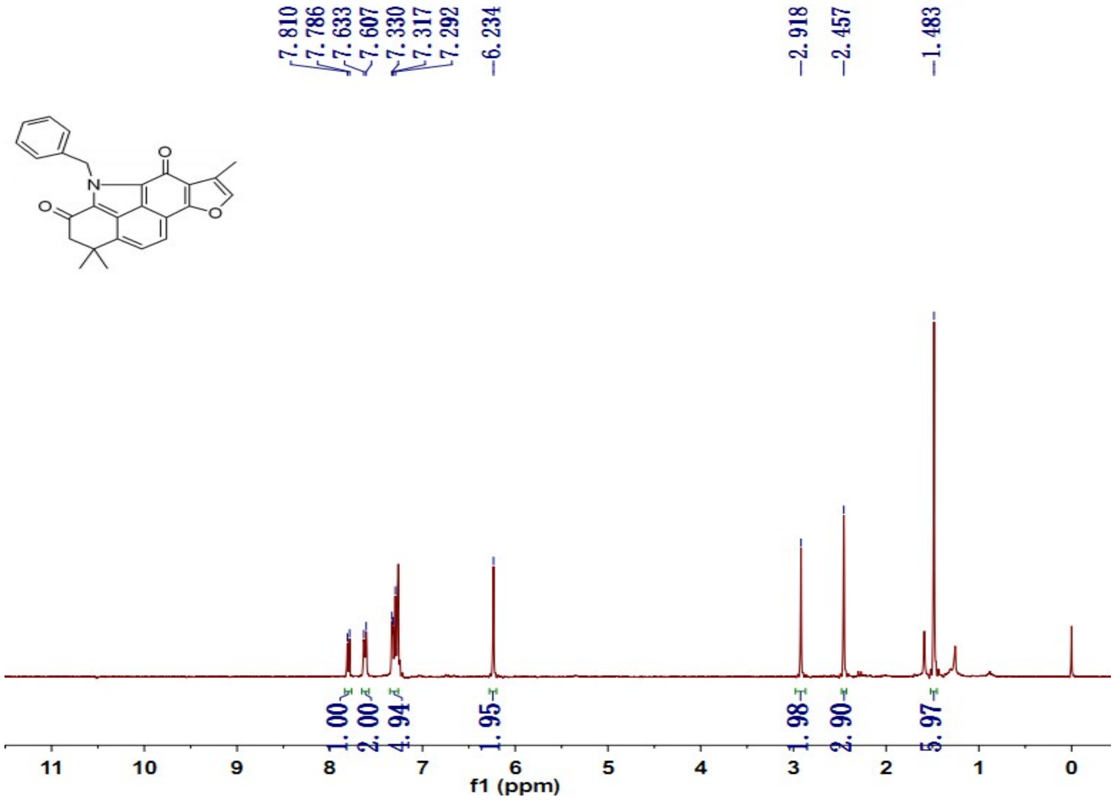

15a: ^{13}C NMR

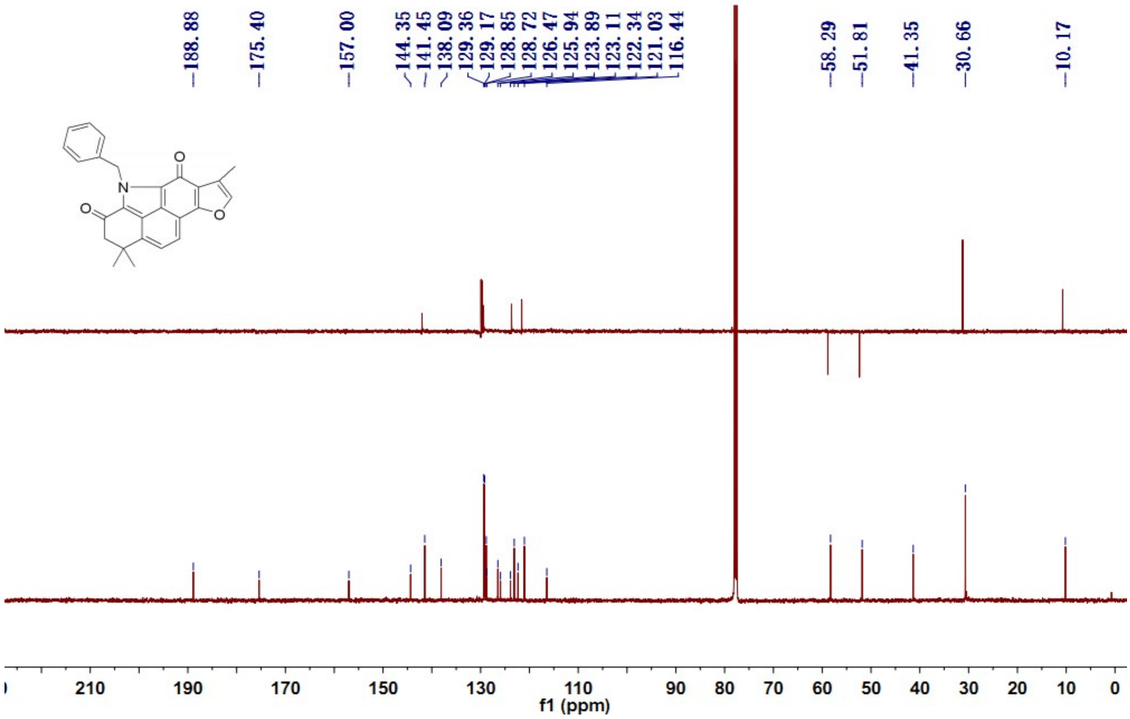

15b: ^{1}H NMR

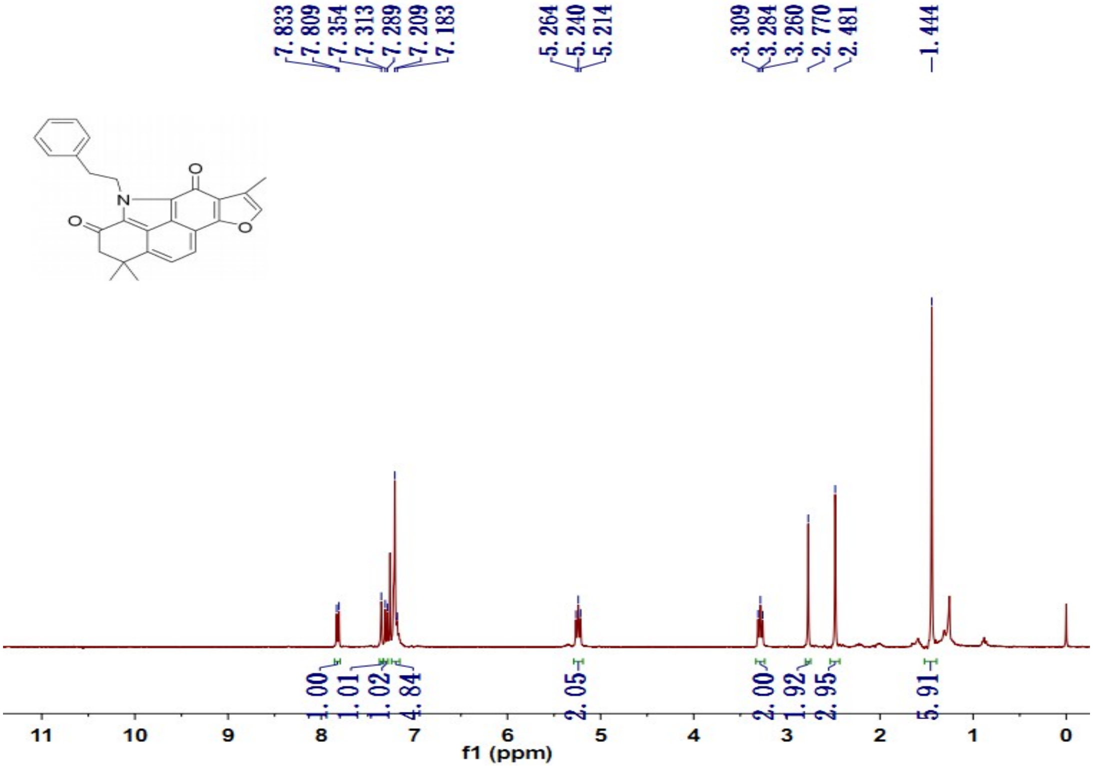

15b: ^{13}C NMR

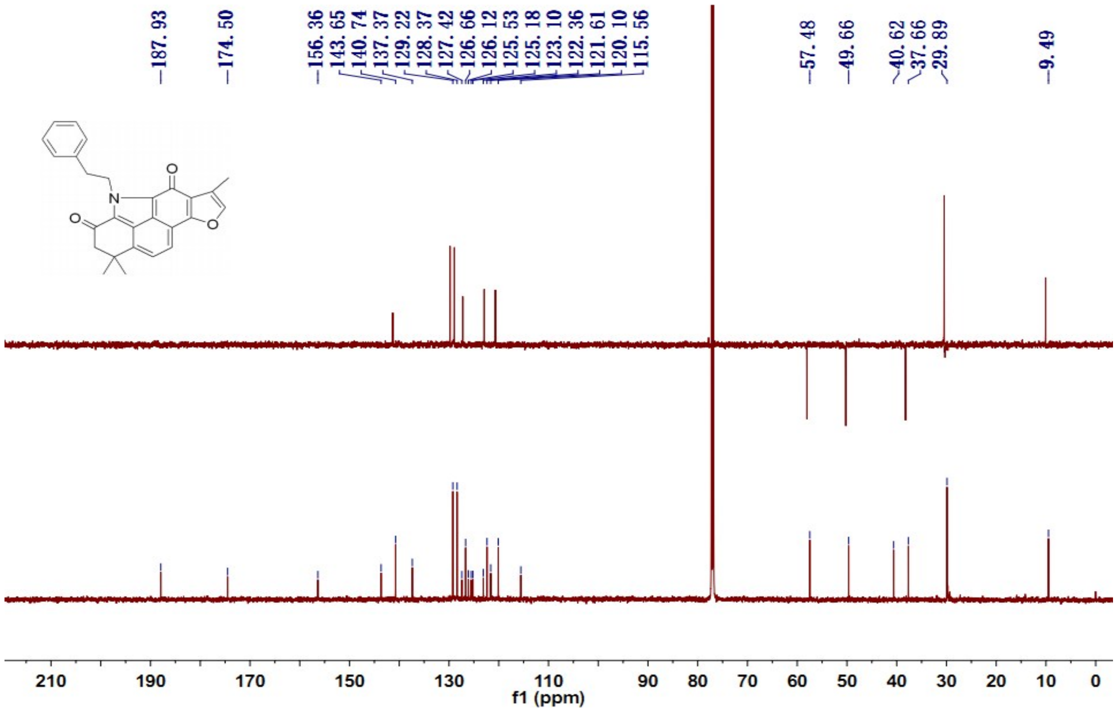

15c: ^{1}H NMR

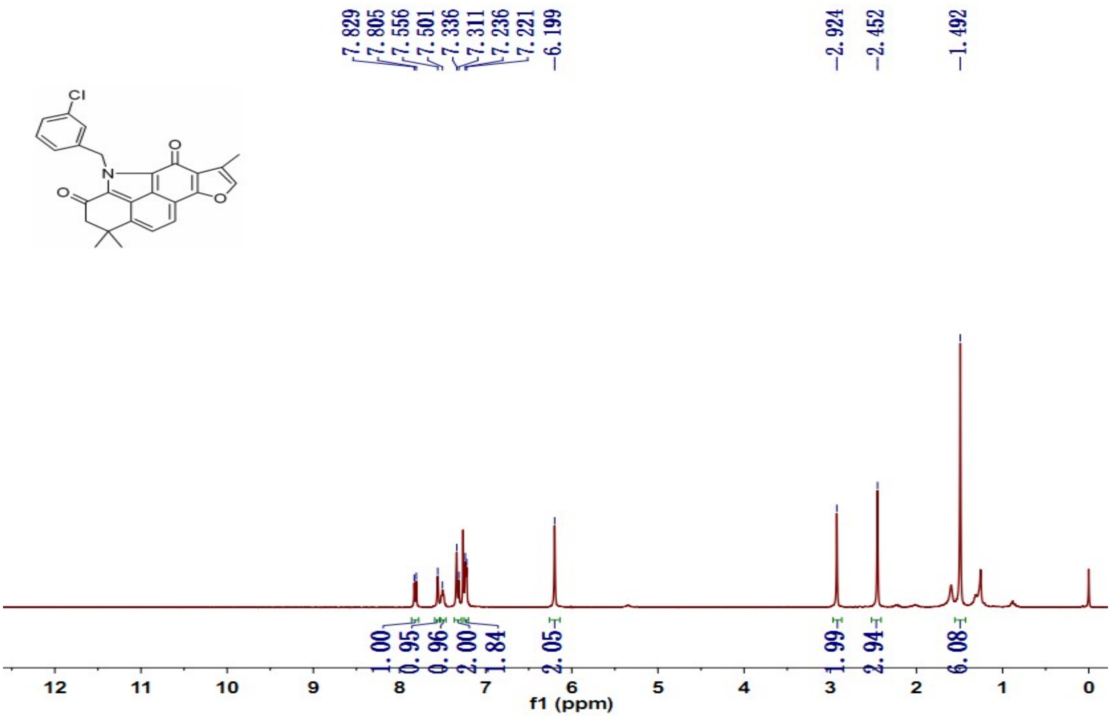

15c: ^{13}C NMR

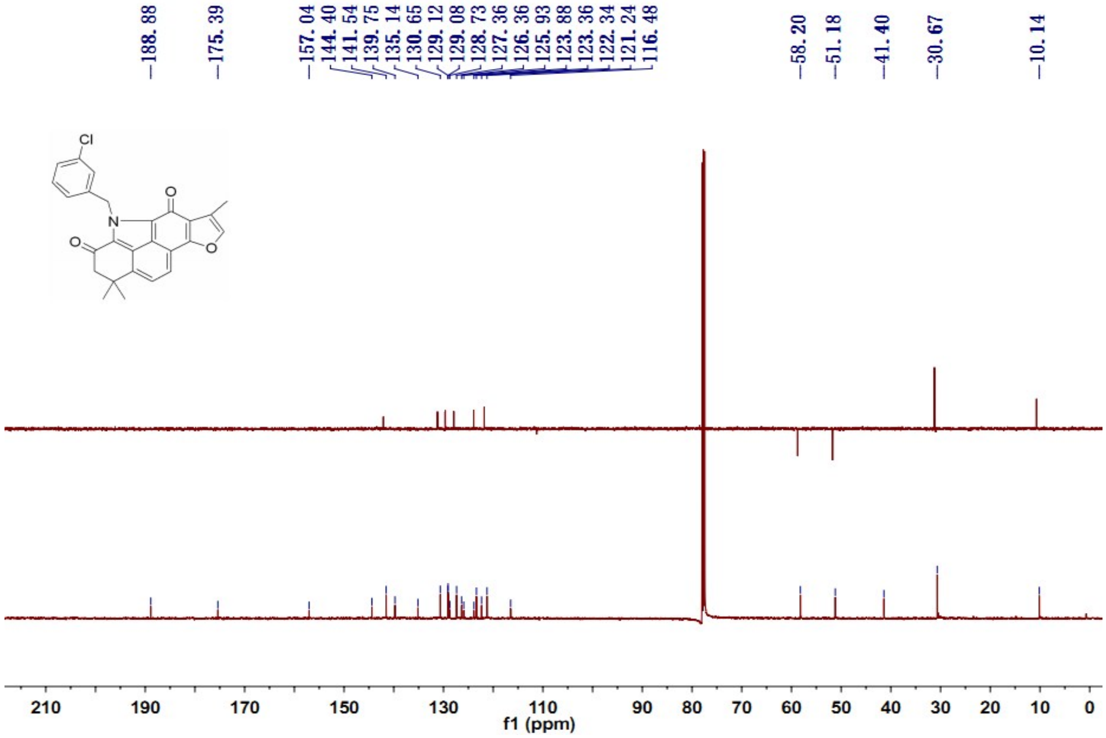

15d: ^{1}H NMR

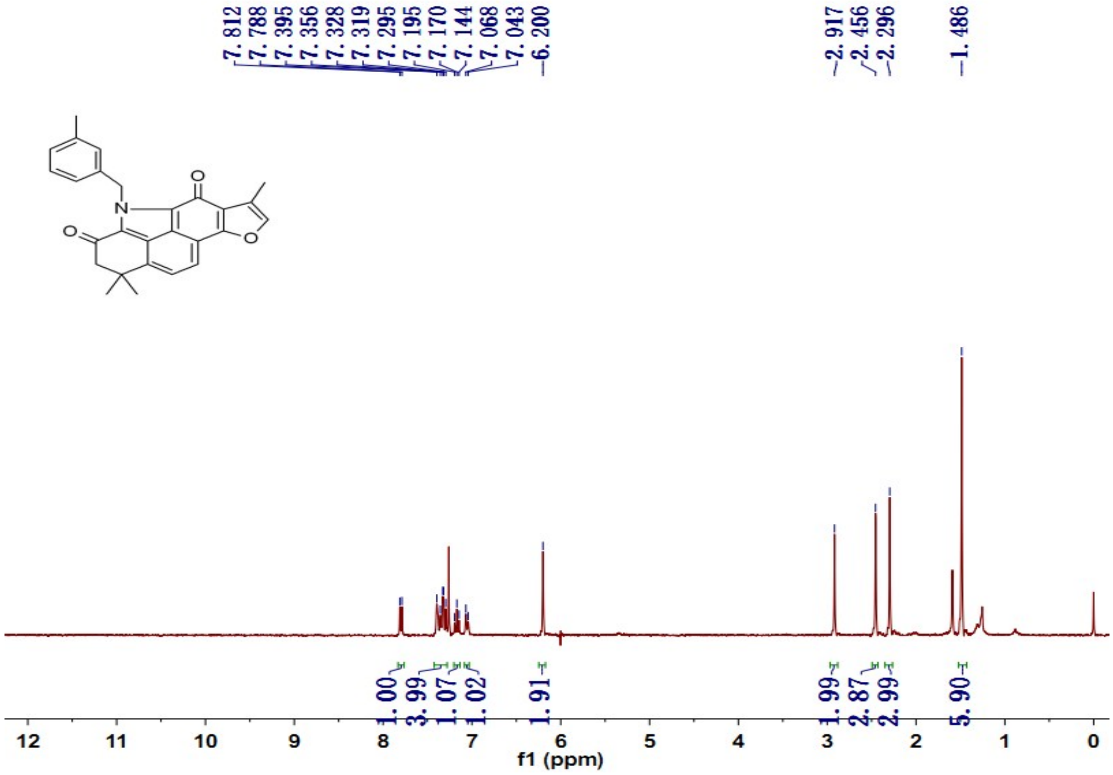

15d: ^{13}C NMR

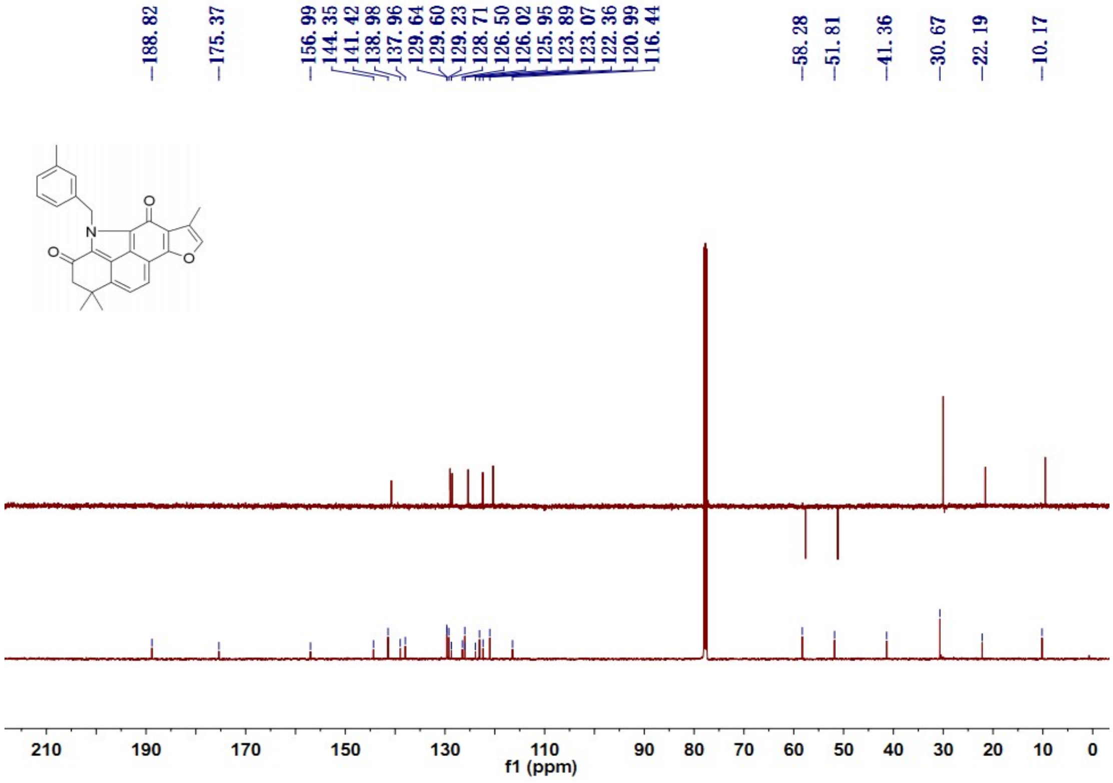

15e: ^{1}H NMR

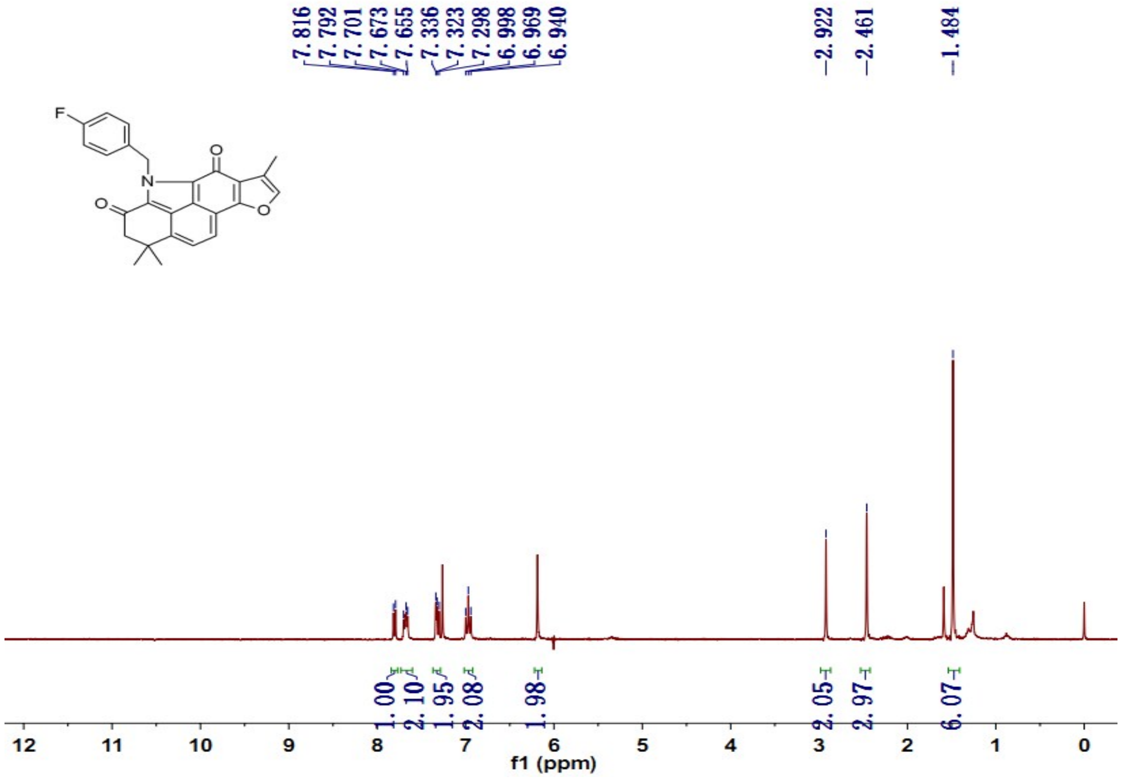

15e: ^{13}C NMR

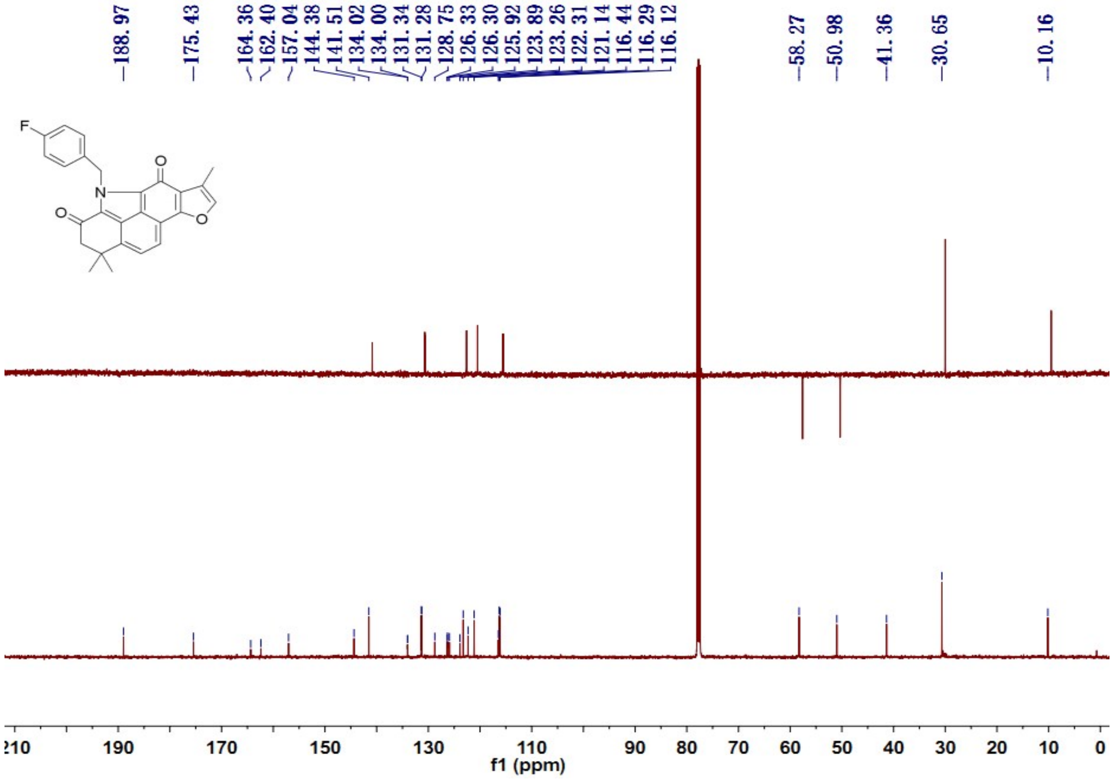

15f:^{1}H NMR

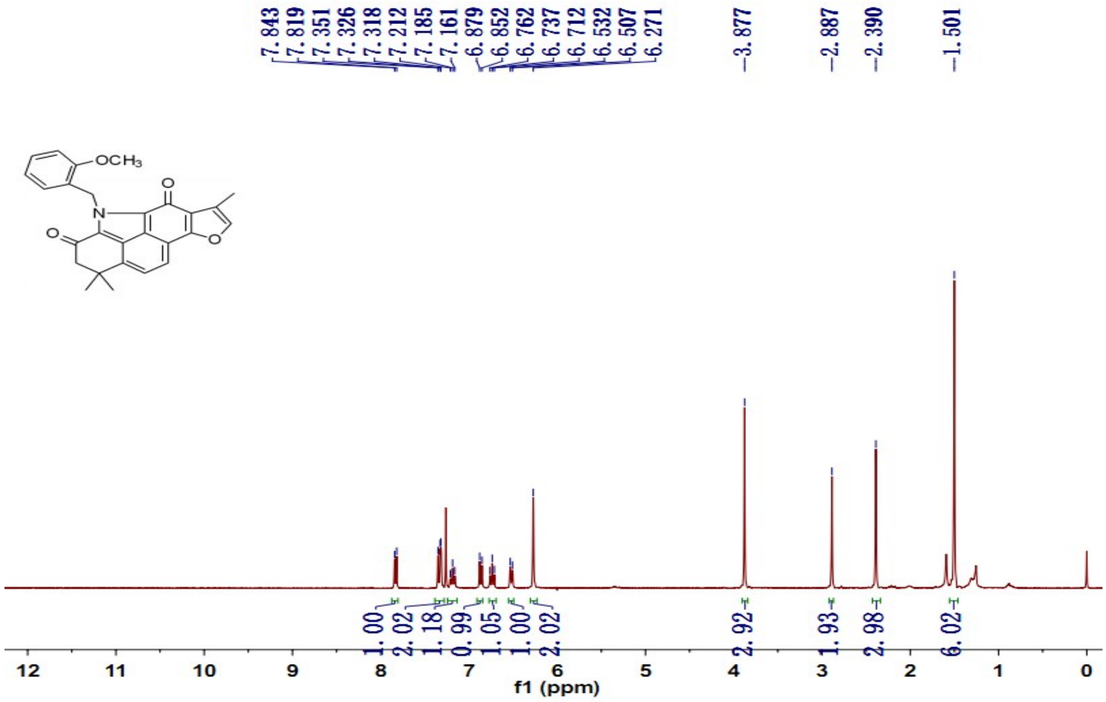

15f: ^{13}C NMR

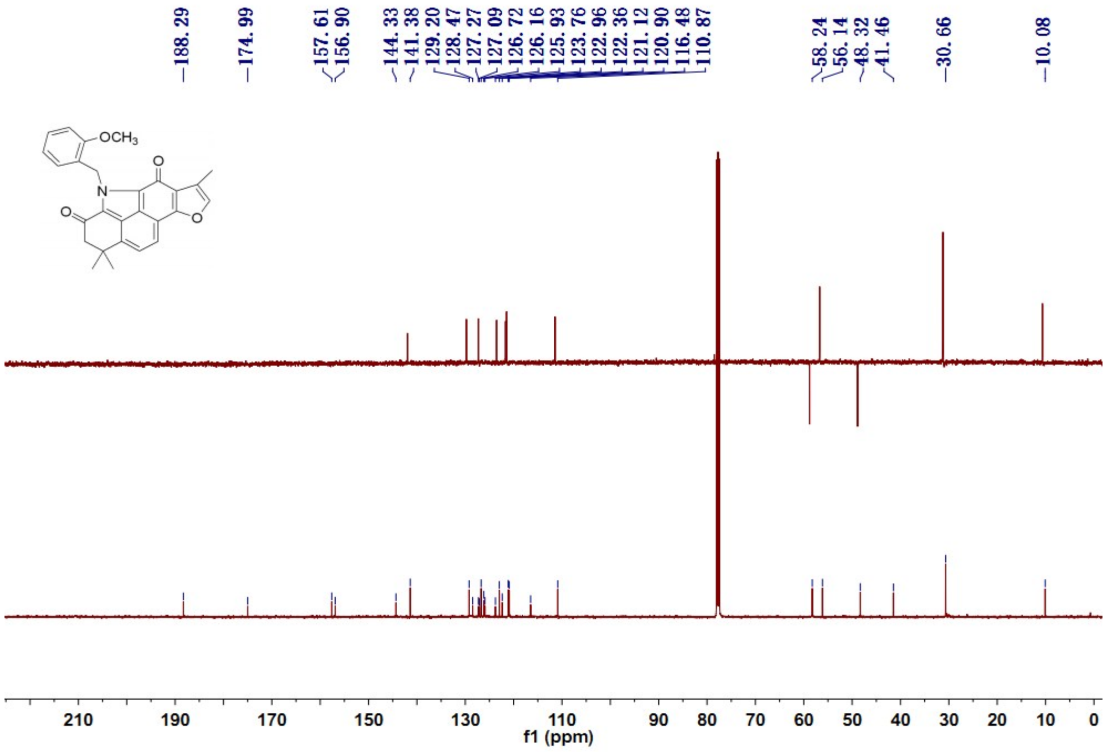

15g:^{1}H NMR

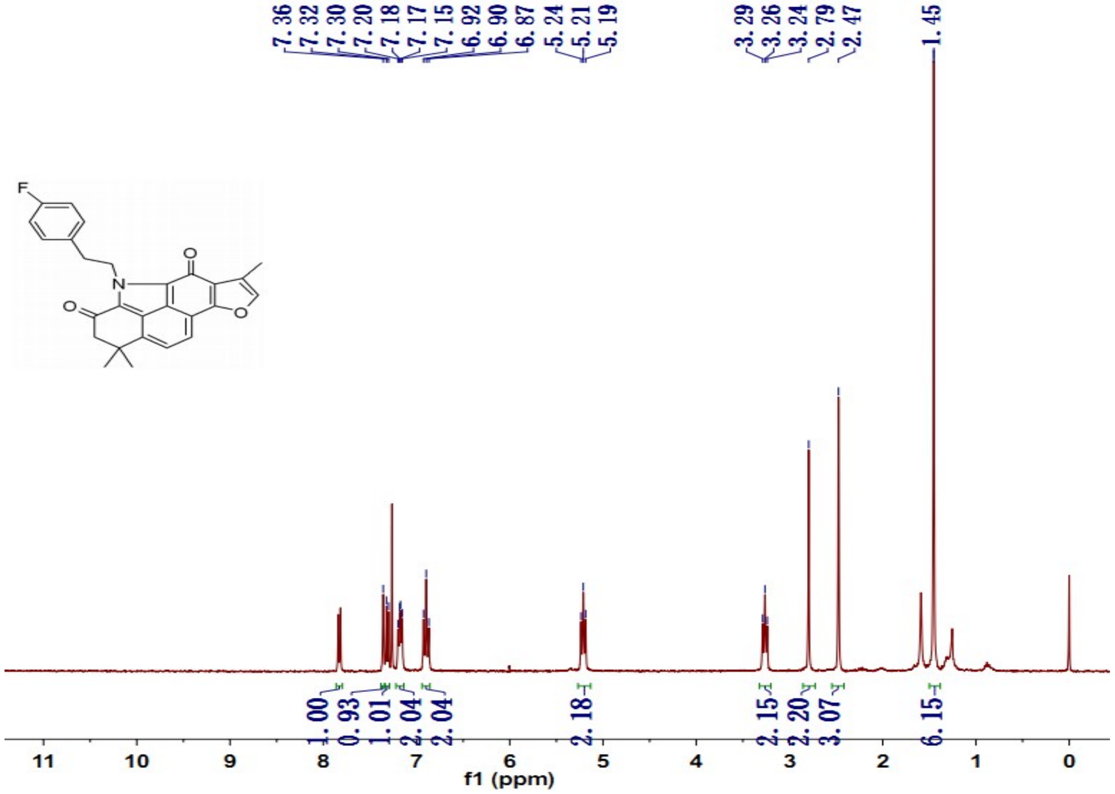

15g: ^{13}C NMR

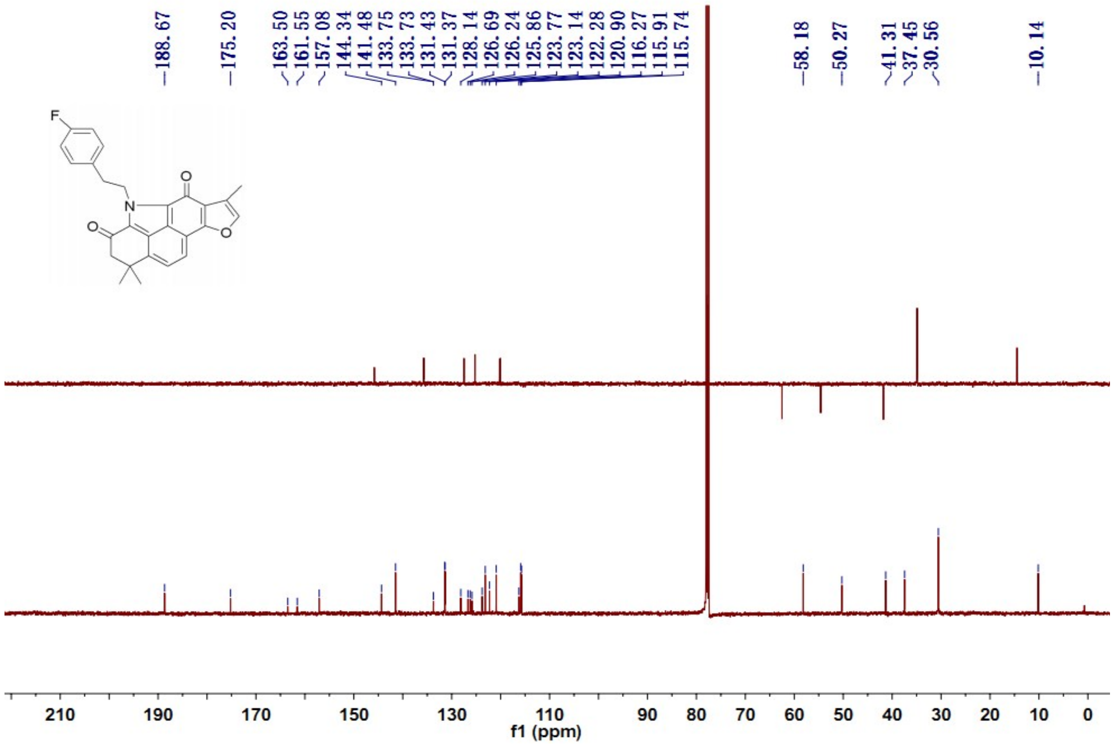

15h:^{1}H NMR

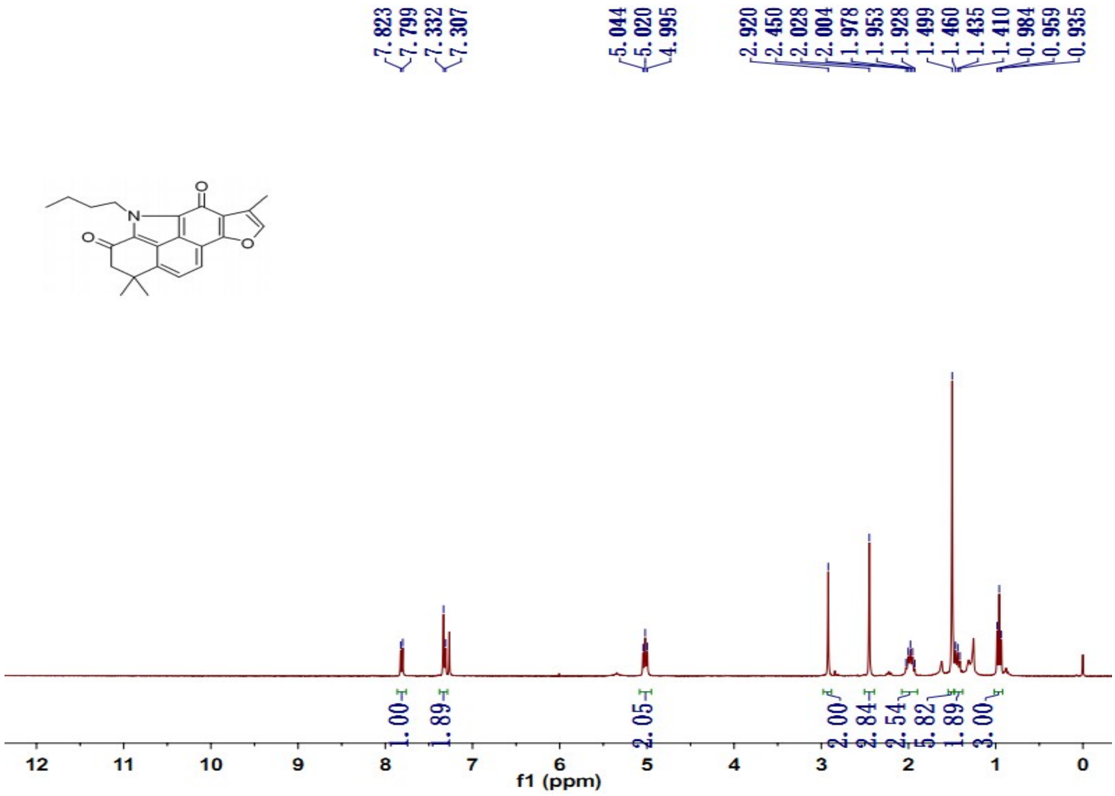

15h: ^{13}C NMR

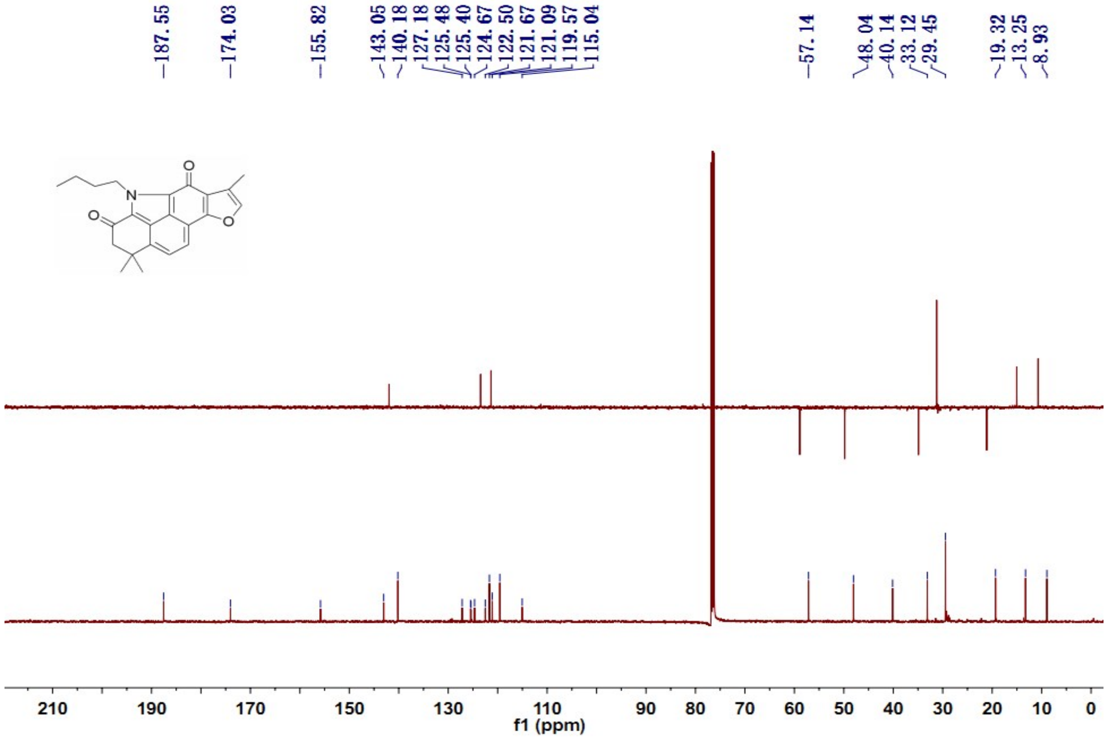

15i:^{1}H NMR

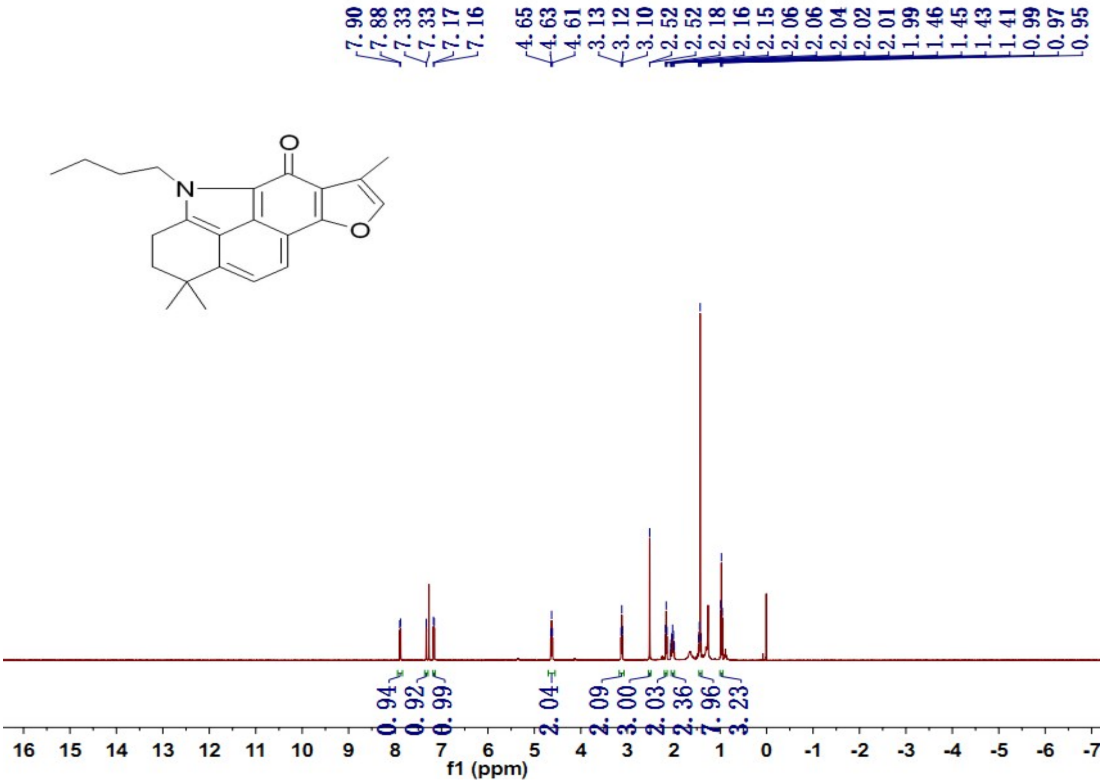

15i: ^{13}C NMR

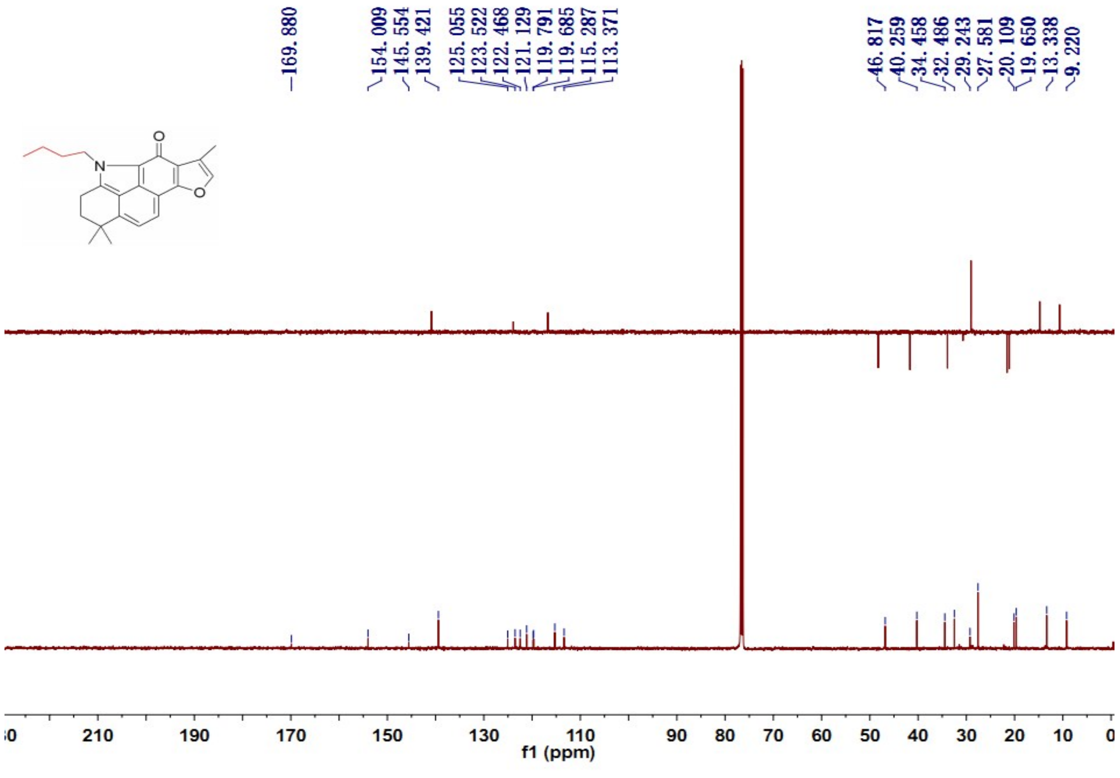

15j: ^{1}H NMR

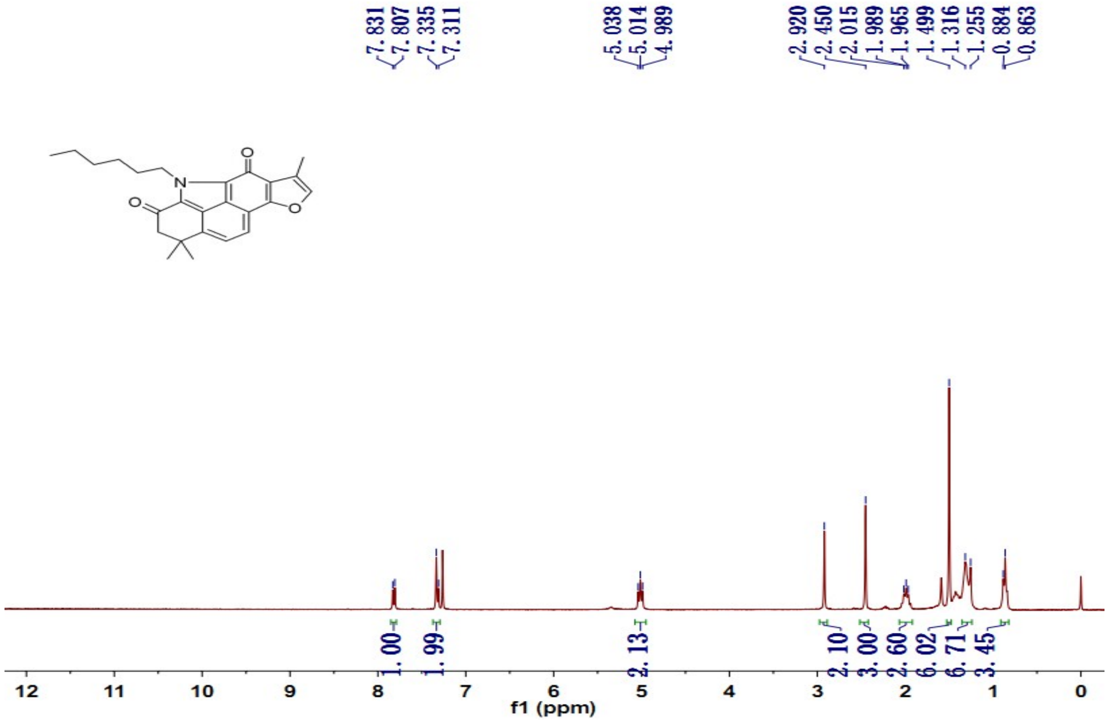

15j: ^{13}C NMR

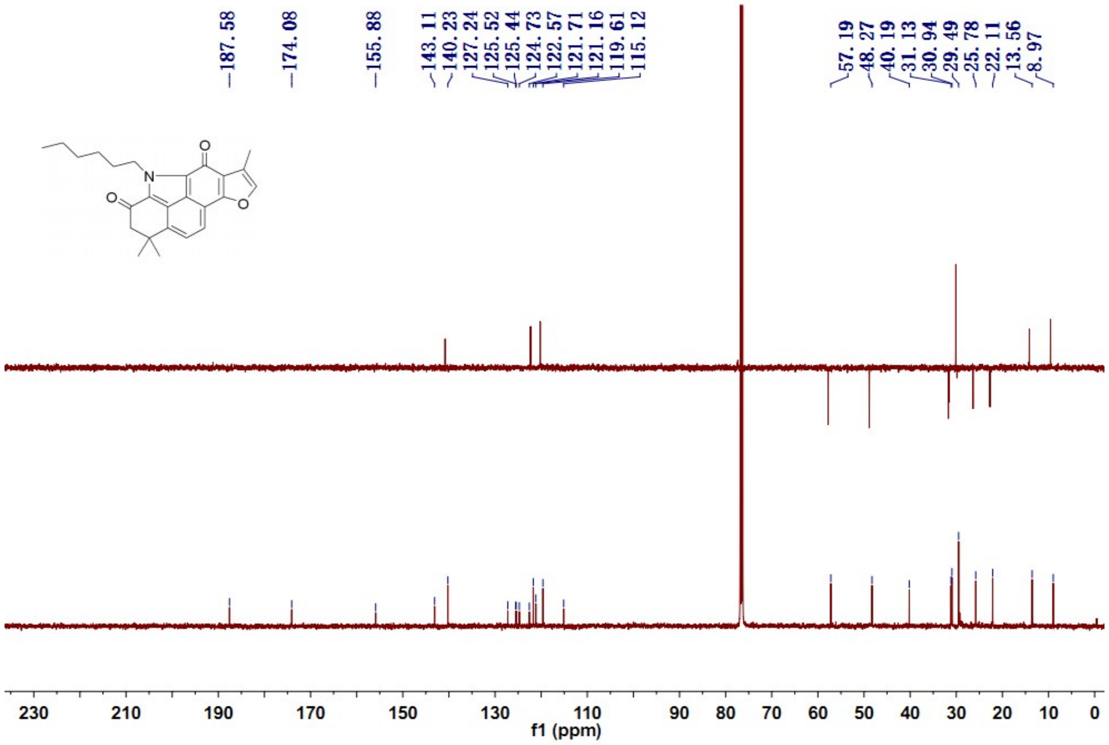

15k:^{1}H NMR

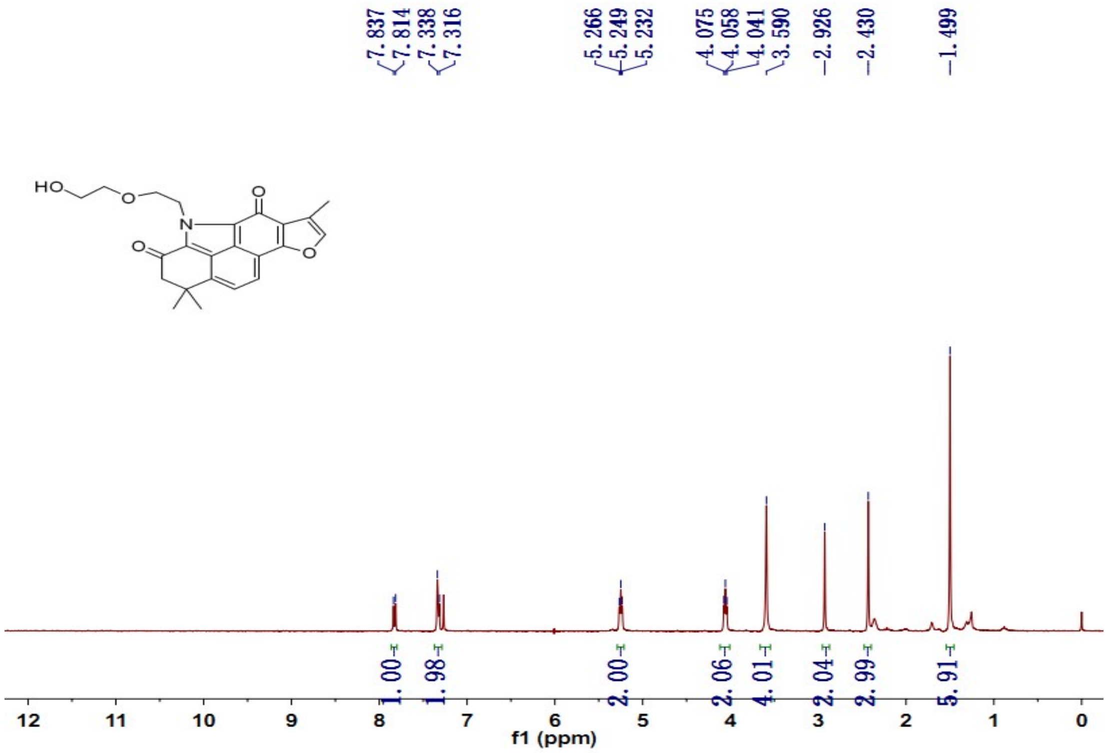

15k: ^{13}C NMR

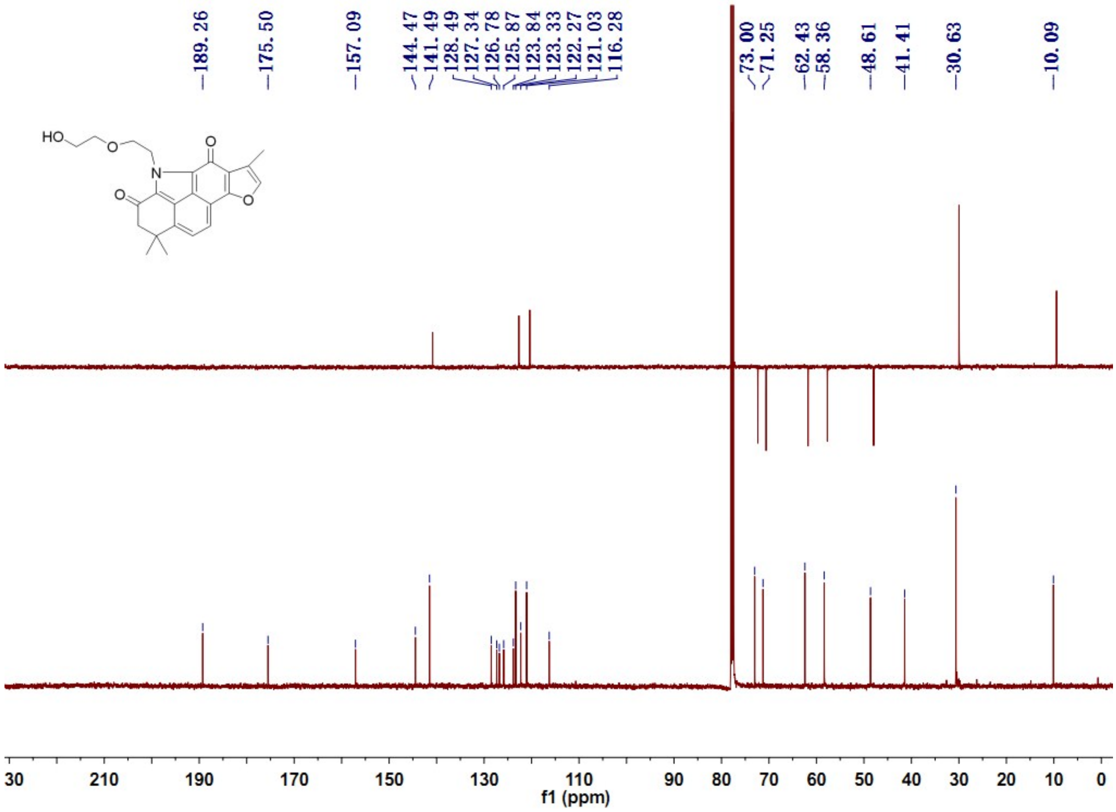

17a: ^{1}H NMR

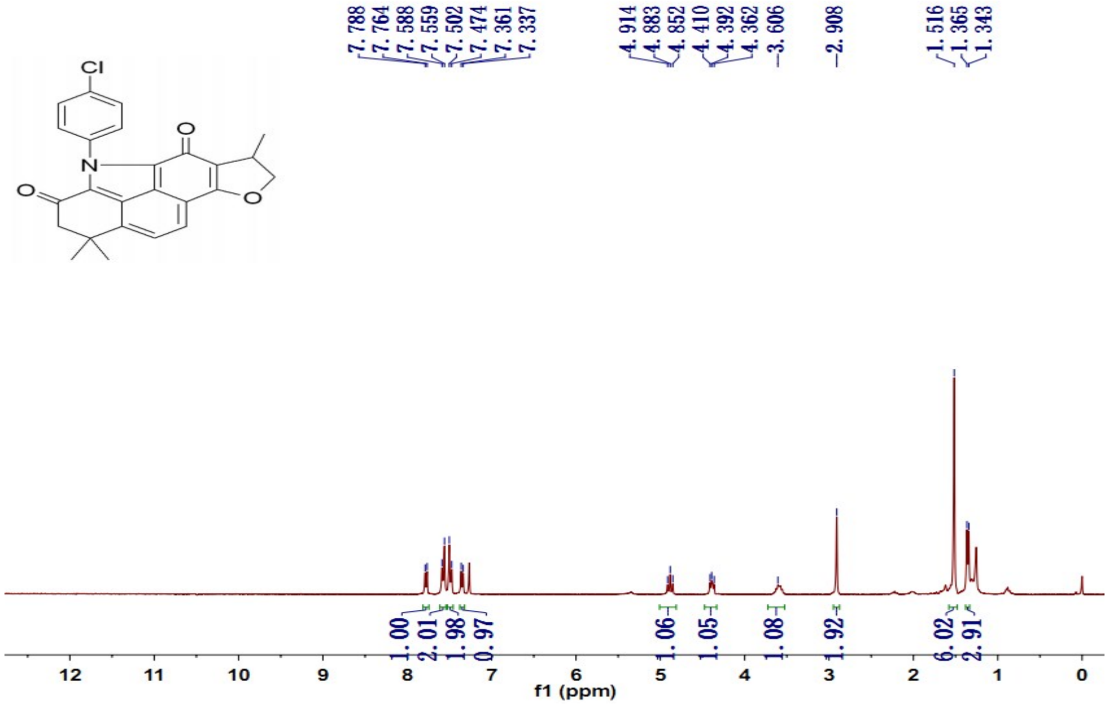

17a: ^{13}C NMR

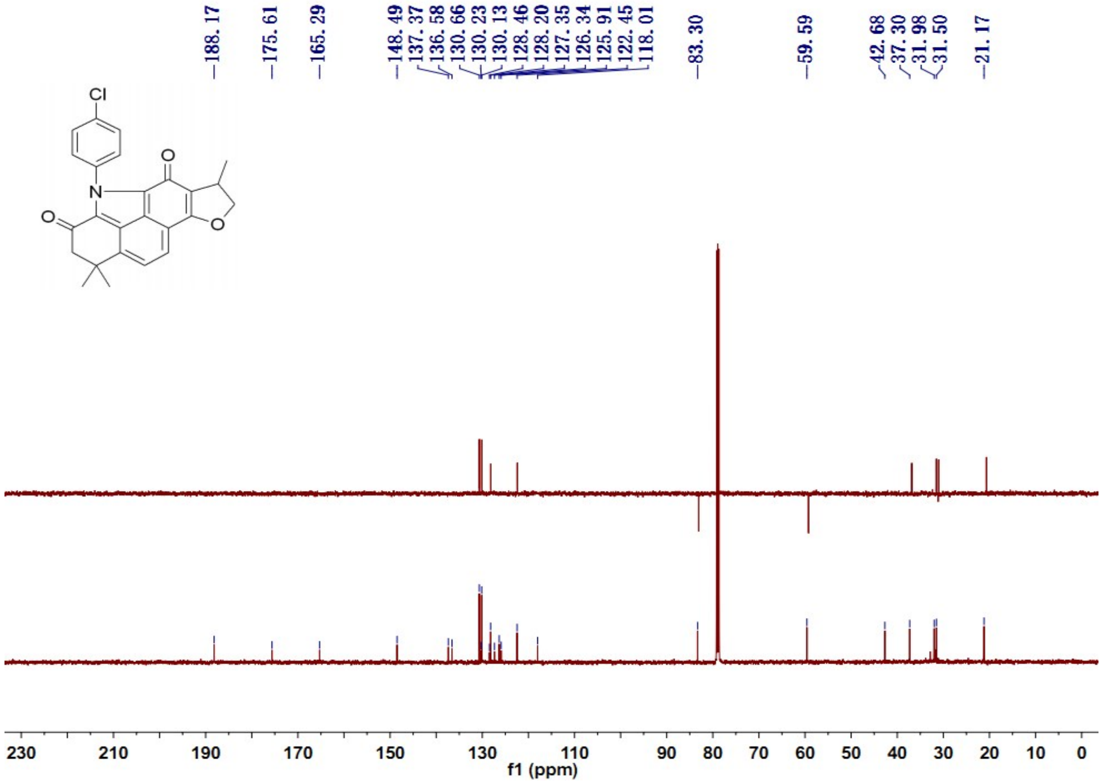

17b: ^{1}H NMR

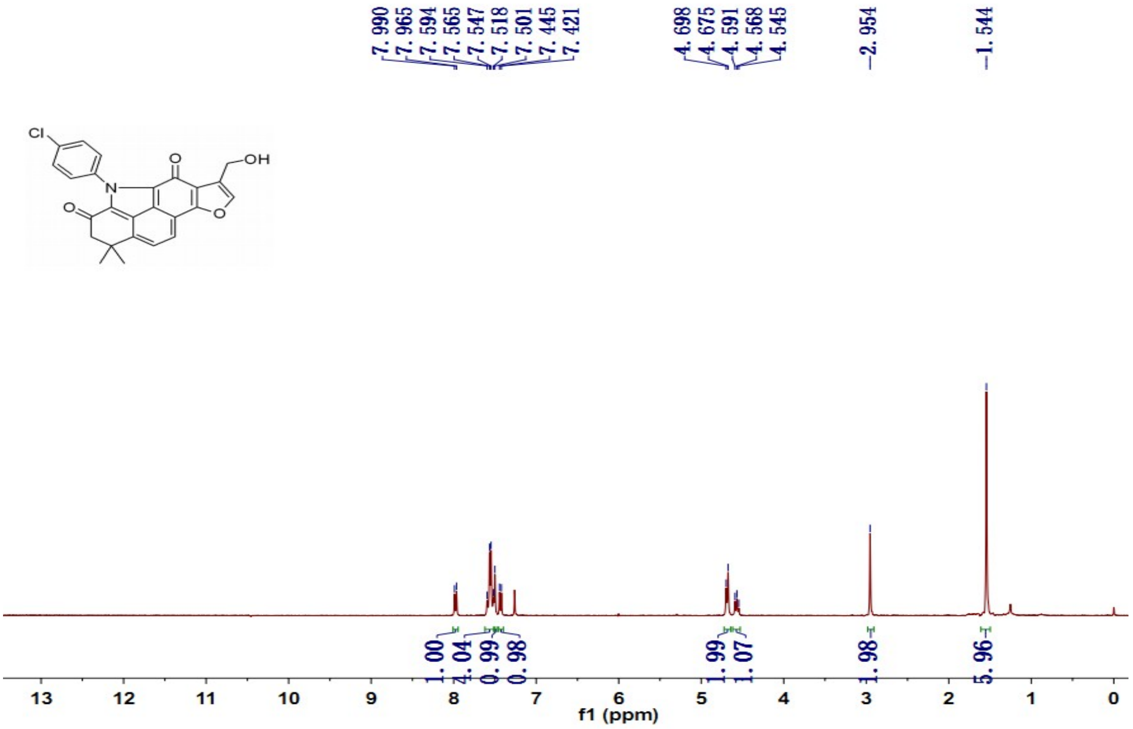

17b: ^{13}C NMR

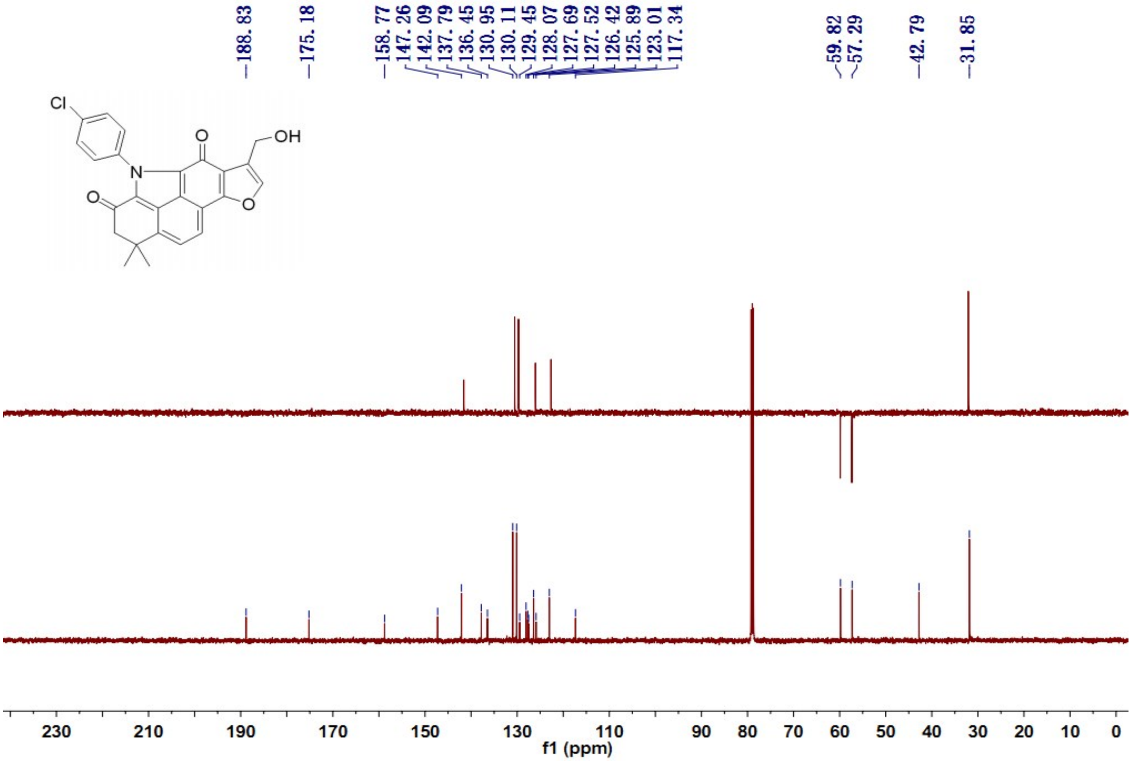

17c :¹H NMR

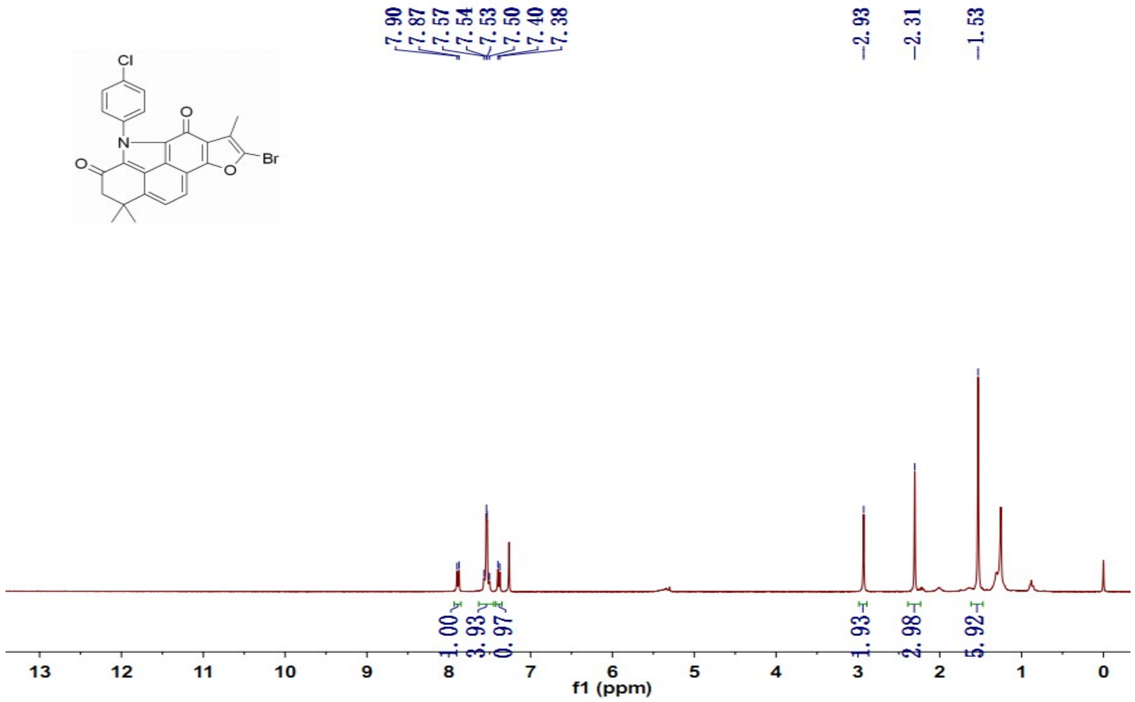

17c: ¹³C NMR

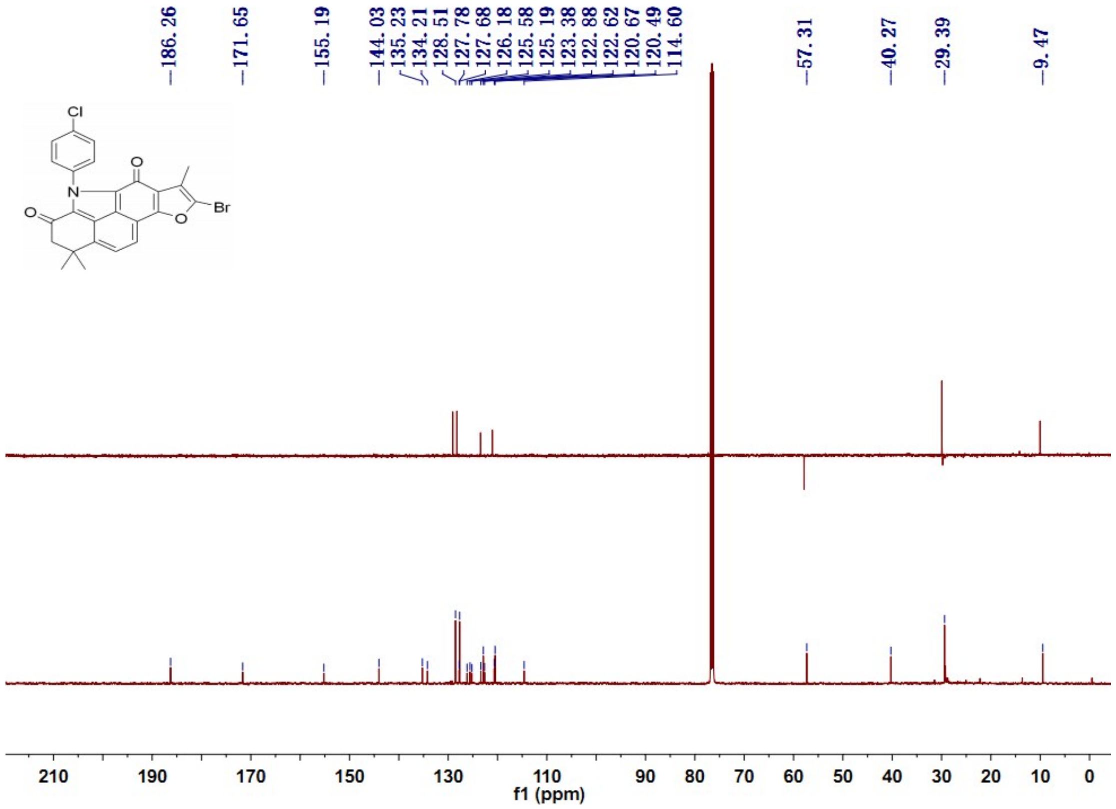

17d :^{1}H NMR

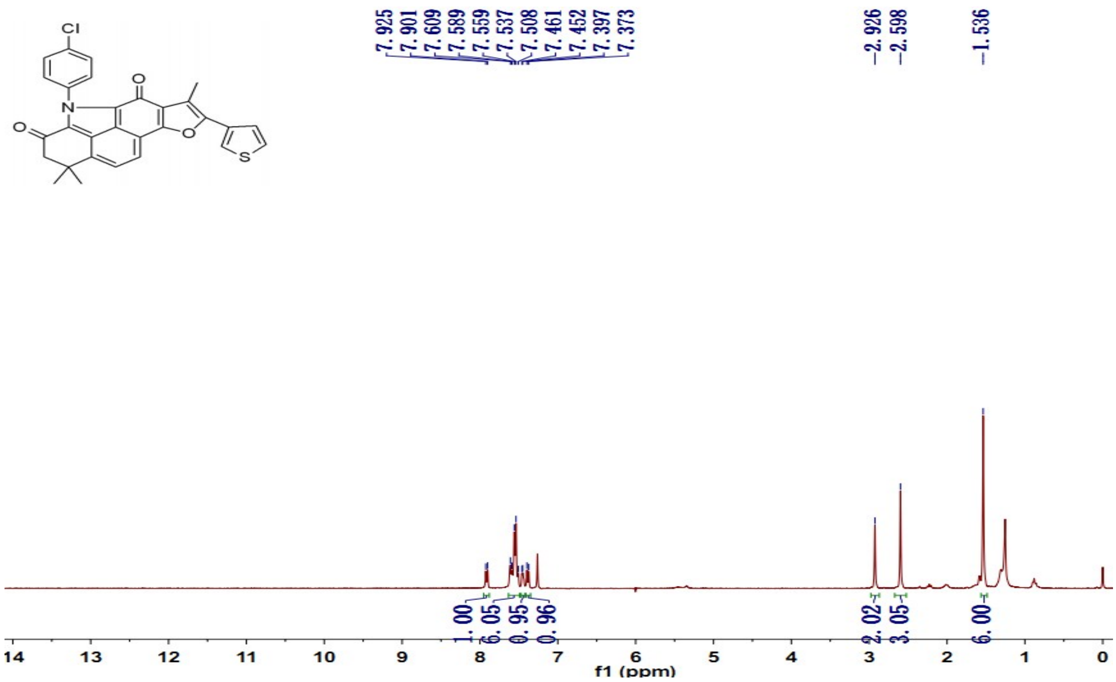

17d: ^{13}C NMR

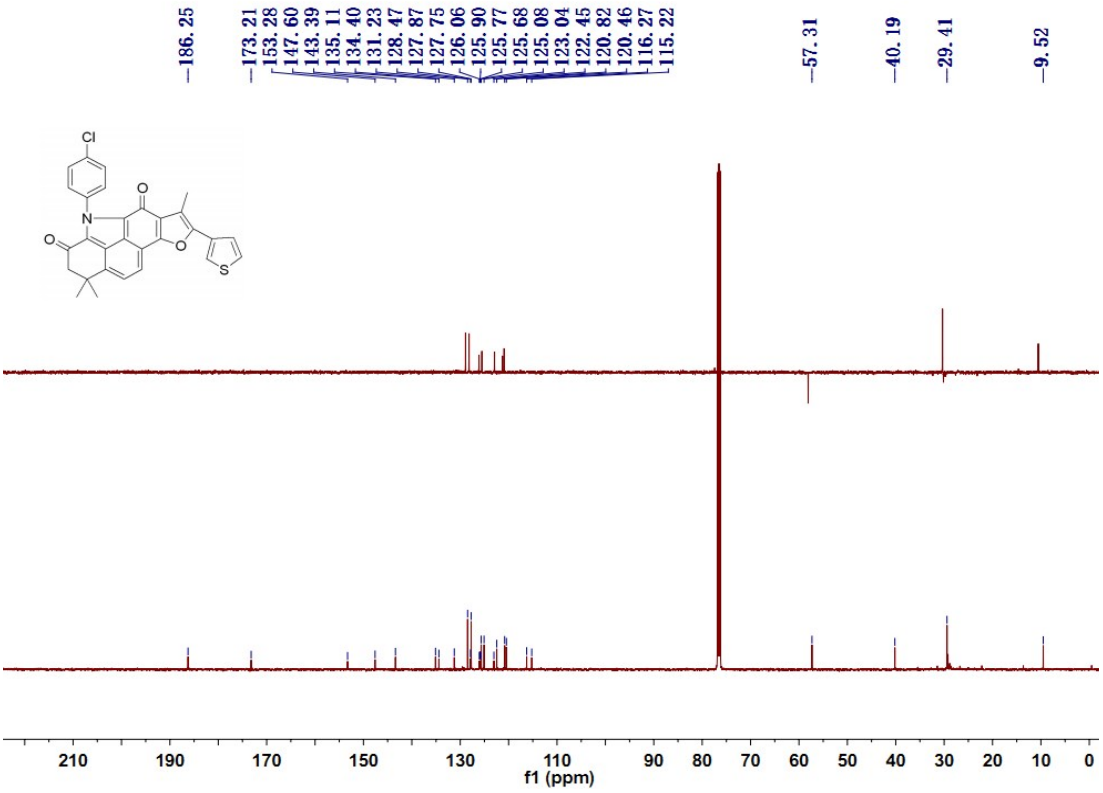

17e :^{1}H NMR

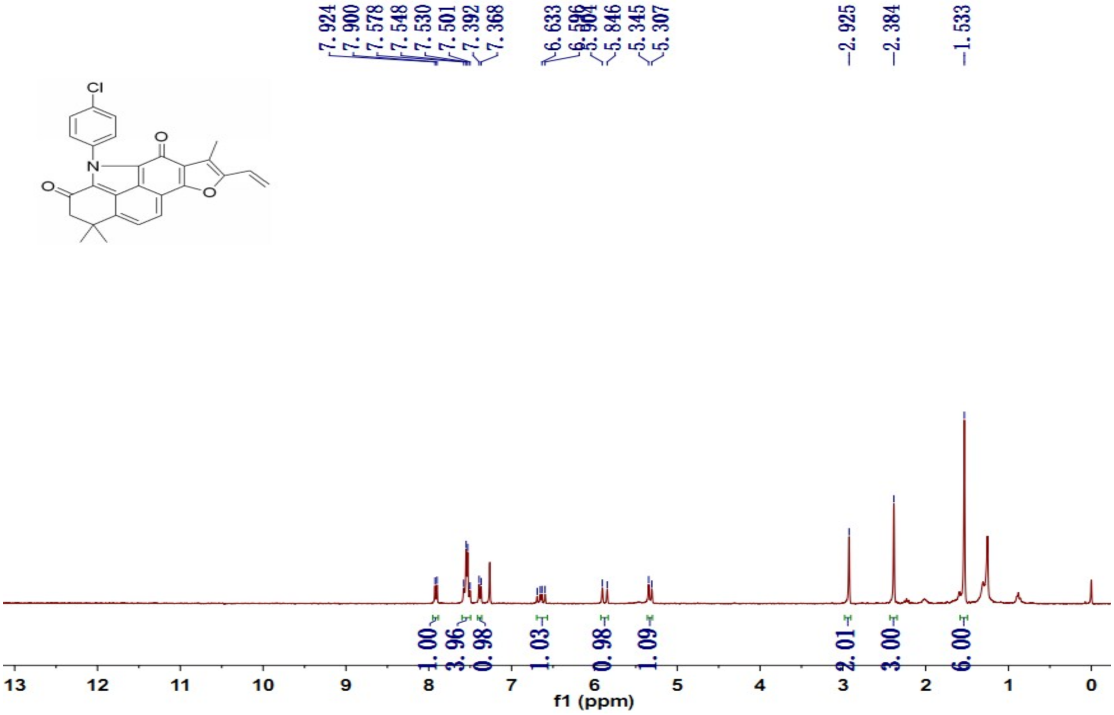

17e: ^{13}C NMR

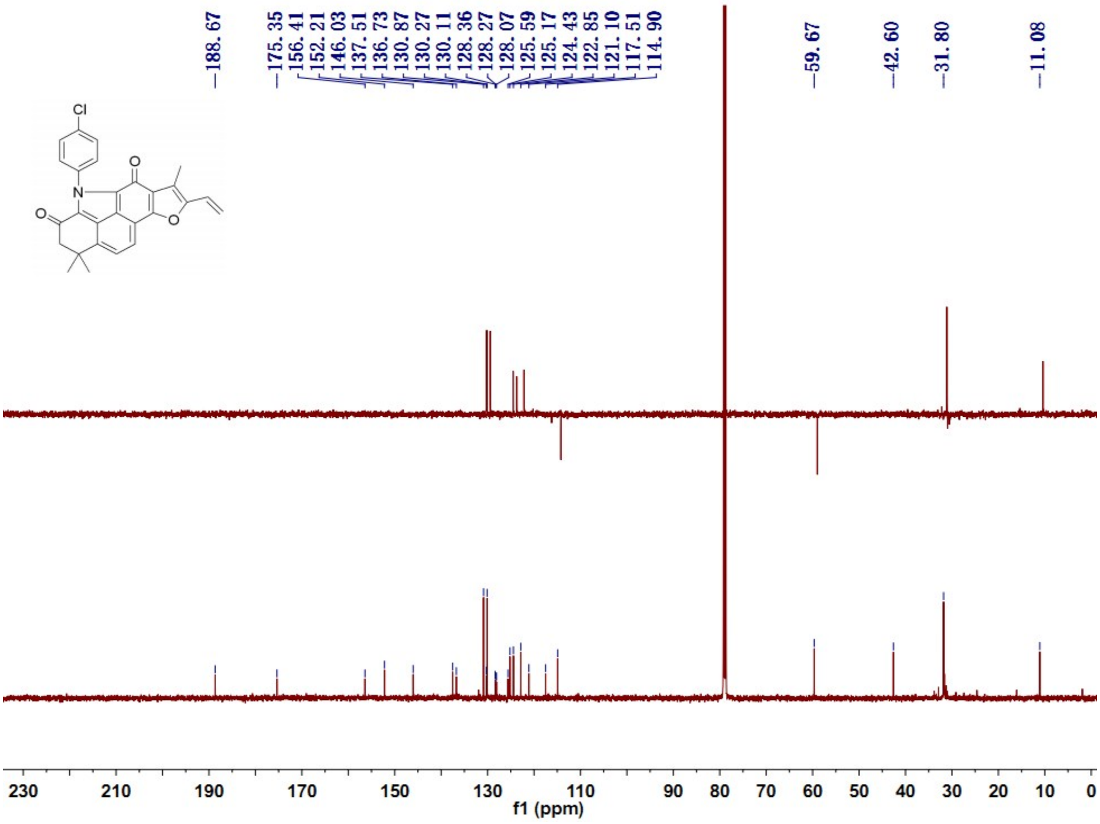

18 : ^{1}H NMR

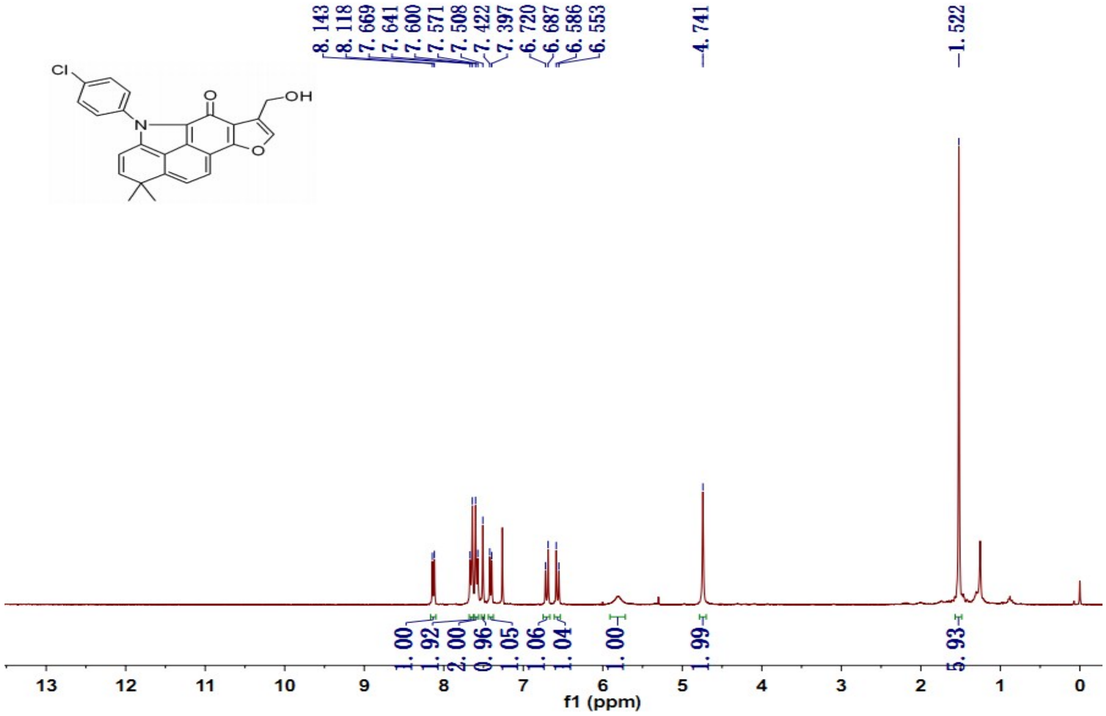

18: ^{13}C NMR

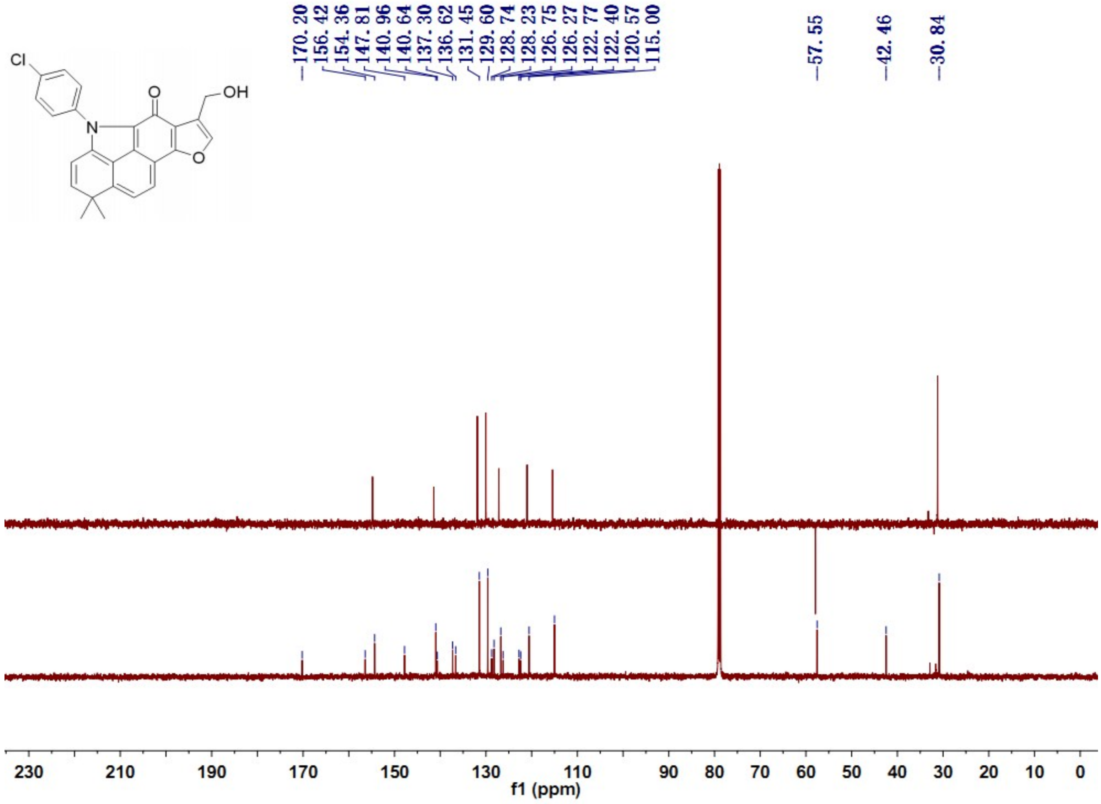

11a: ^{1}H NMR

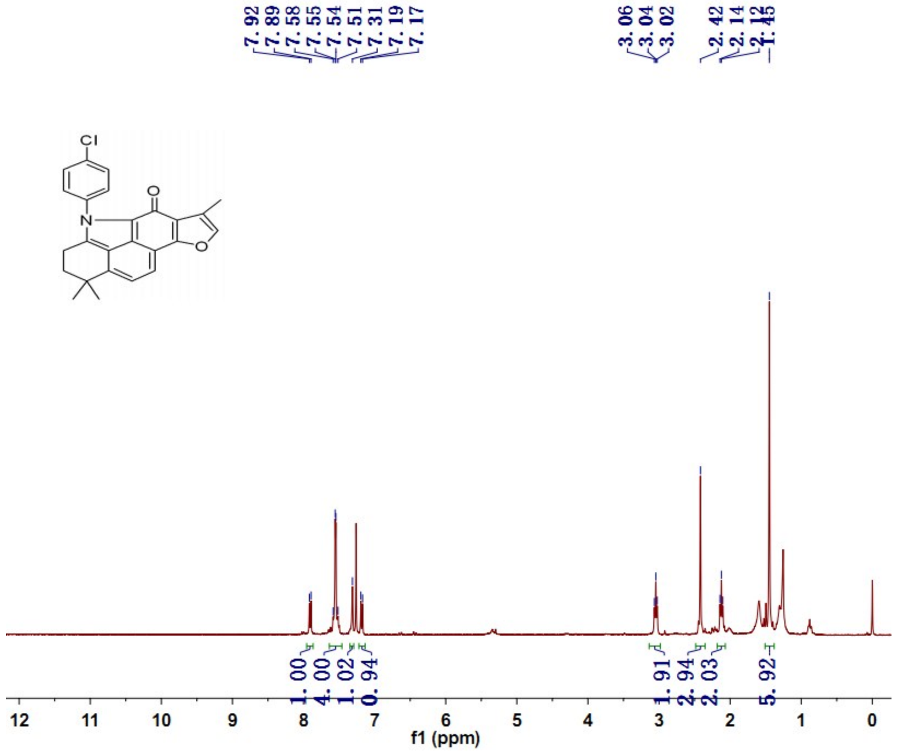

11a: ^{13}C NMR

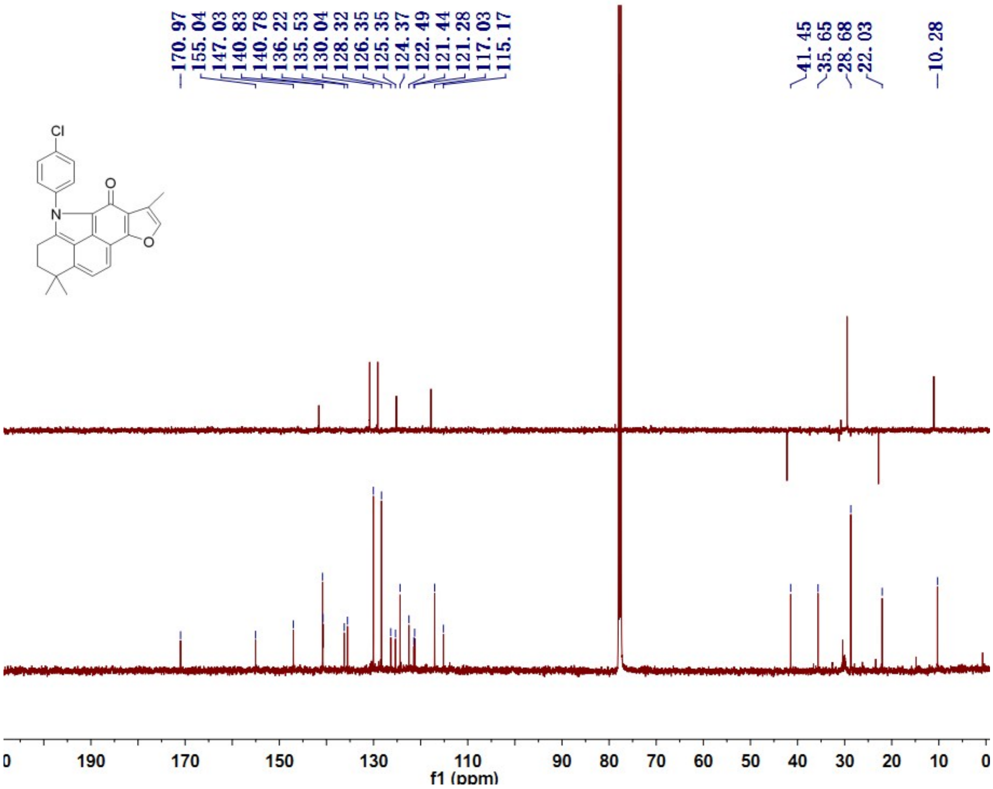

11b:^{1}H NMR

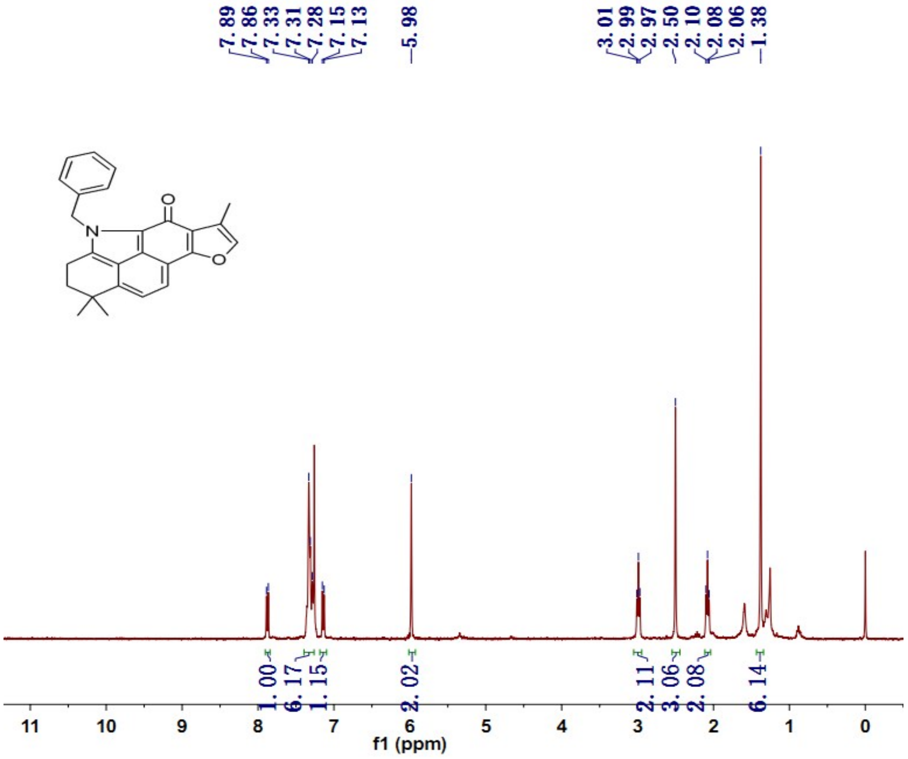

11b: ^{13}C NMR

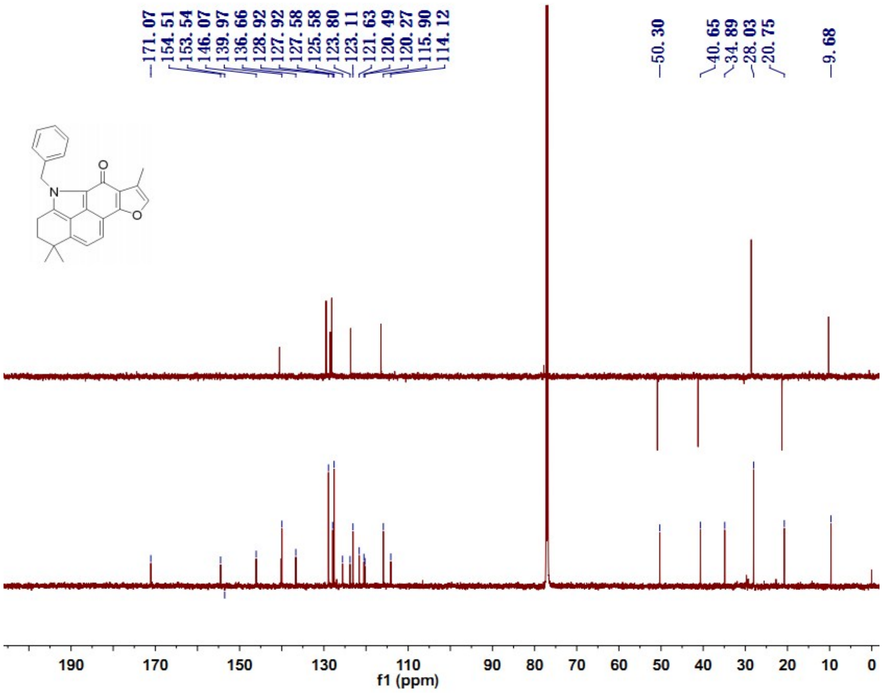

Supplement: Supplementary file 1 [file SC-010-C9SC00086K-s001.pdf]
